# Novel blood‐based tumor mutation algorithm and nomogram predict survival of immune checkpoint inhibitor in non‐small‐cell lung cancer: Results from two multicenter, randomized clinical trials

**DOI:** 10.1002/ctm2.53

**Published:** 2020-06-05

**Authors:** Yunfang Yu, Yongjian Chen, Anlin Li, Qiyun Ou, Qingjian Li, Yang Gu, Dagui Lin, Wenda Zhang, Jingshu Wang, Xudong Tang, Zhihua Li, Hai Hu, Herui Yao

**Affiliations:** ^1^ Guangdong Provincial Key Laboratory of Malignant Tumor Epigenetics and Gene Regulation Department of Medical Oncology Phase I Clinical Trial Centre Sun Yat‐sen Memorial Hospital Sun Yat‐sen University Guangzhou Guangdong China; ^2^ Department of Medical Oncology The Third Affiliated Hospital of Sun Yat‐sen University Guangzhou China; ^3^ Guangdong Medical University Zhanjiang Guangdong China; ^4^ State key laboratory of Oncology in South China Collaborative Innovation Center for Cancer Medicine Sun Yat‐sen University Cancer Center Guangzhou China

Dear Editor,

Our previous study has demonstrated non‐small‐cell lung cancer (NSCLC) patients with high tissue‐based tumor mutation burden (TMB) derived encouraging benefits from immune checkpoint inhibitor (ICI).^1^ The blood‐based TMB (bTMB) recently emerged as an encouraging non‐invasive approach to predict improvements in PFS of atezolizumab over docetaxel among previously treated NSCLC patients, but it failed to reproducibly stratify patients into groups with different OS.^2^ The ctDNA maximum somatic allele frequency (MSAF) has been demonstrated affects the concordance between bTMB and tissue‐based TMB,^2^, ^3^ suggesting MSAF might potentially provide additional predictive value for the bTMB. Herein, we first performed individual patient data meta‐analysis using OAK and POPLAR randomized trial data^4^, ^5^ to assess the predictive value of adding MSAF to bTMB (bTMB‐MSAF algorithm) in identifying NSCLC patients who could benefit from atezolizumab over docetaxel. Moreover, we comprehensively investigated the blood‐based mutational landscape of NSCLC patients and developed nomogram considering oncogenic and clinicopathological variables to predict survival of ICI patients. Full methods are described in the Supporting Information.

In the whole intention‐to‐treat and EGFR wild‐type patients, compared with docetaxel, atezolizumab resulted in significantly longer OS (HR 0.72, 95% CI, 0.62‐0.83; *P *< .001; HR 0.67, 95% CI, 0.57‐0.78; *P *< .001; Figure 1A,B), but not PFS (Figures S2 and S3). EGFR mutant patients did not show significant difference in OS or PFS between two treatments (Figure S4). More clinicopathological subgroups are presented in Supporting Information Result S1 and Figures S5 and S6.

**FIGURE 1:**
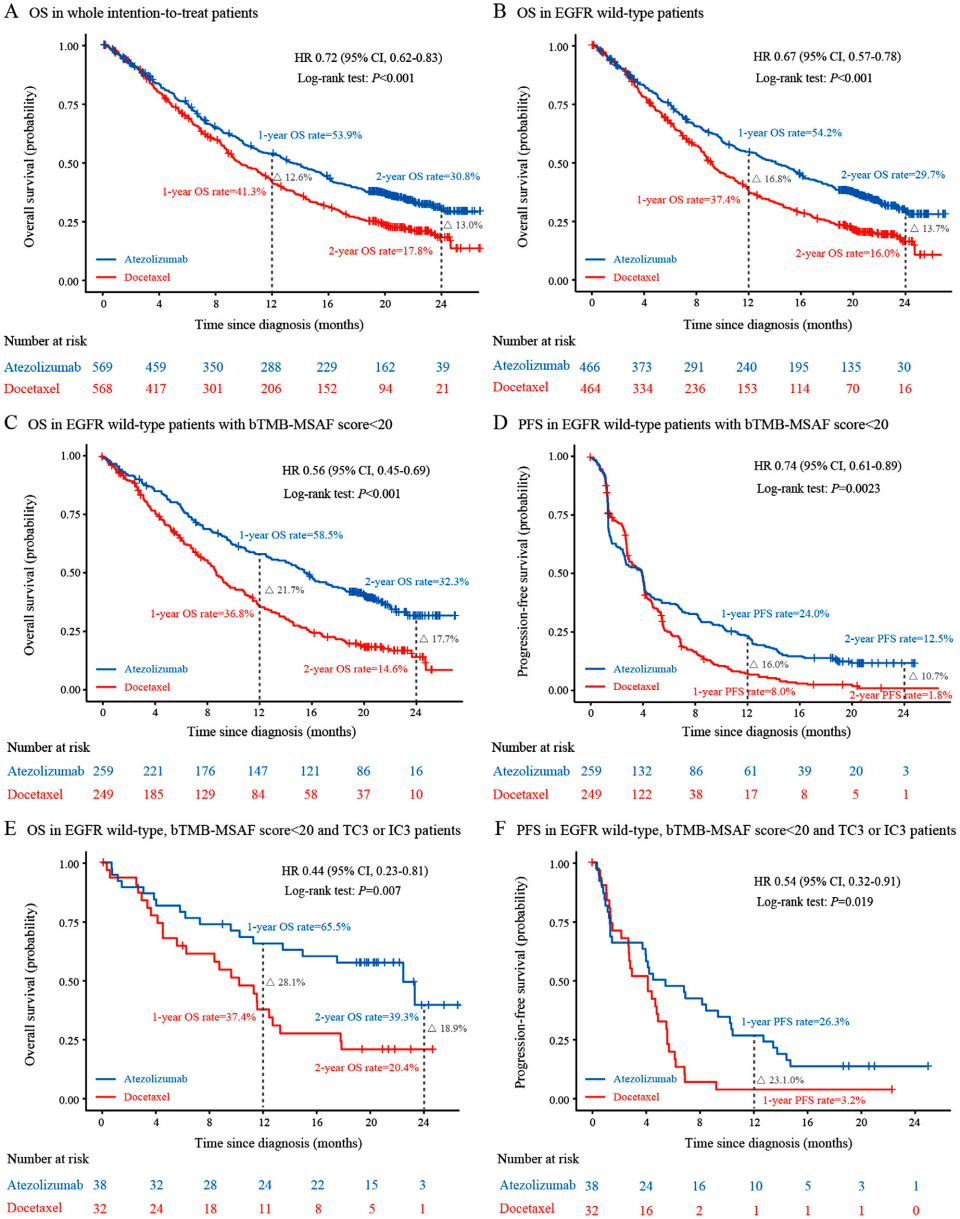
Survival analysis stratified by treatment. A, OS in the whole intention‐to‐treat patients. B, OS in EGFR wild‐type patients. C and D, OS and PFS in EGFR wild‐type patients with a bTMB‐MSAF score < 20, respectively. E and F, OS and PFS in EGFR wild‐type patients with a bTMB‐MSAF score < 20 and PD‐L1 expression of TC3 or IC3, respectively. HR, hazard ratio; CI, confidence interval; OS, overall survival; PFS, progression‐free survival; PD‐L1, programmed cell death ligand 1. TC3 or IC3 indicates that over 50% of tumor cells or over 10% of tumor infiltrating immune cells expressed PD‐L1

We found none of tested bTMB cut‐points could classify patients into groups with consistent differences in PFS and OS between atezolizumab and docetaxel in the EGFR wild‐type patients (Figure S7). Of note, a previously established bTMB threshold of 16^2^ even produced paradoxical results; patients with bTMB < 16 had significant improvement in OS (HR 0.68, 95% CI, 0.52‐0.88) but not in PFS (HR 0.99, 95% CI, 0.82‐1.19); patients with bTMB ≥ 16 had significant improvement in PFS (HR 0.58, 95% CI, 0.36‐0.93) but not in OS (HR 0.48, 95% CI, 0.23‐1.02; Figure S7).

We next performed similar analysis at various MSAF cut‐points and found EGFR wild‐type patients with MSAF < 0.10 had improved OS (HR 0.62, 95% CI, 0.51‐0.75) and PFS (HR 0.83, 95% CI, 0.70‐0.98) when treated with atezolizumab compared with docetaxel, whereas patients with MSAF ≥ 0.10 showed no difference in OS or PFS (Figures S8‐S11). Additionally, a low MSAF significantly predict favorable OS and PFS among each of entire patients and atezolizumab‐treated patients (Figure S9). None of the tested bTMB or MSAF cut‐points could classify EGFR mutant patients into groups with different PFS or OS two treatments (Figures S10 and S11).

We further determined a novel bTMB‐MSAF algorithm outperforming bTMB in predicting survival with atezolizumab versus docetaxel. Patients with lower versus higher bTMB‐MSAF scores had significantly better OS and PFS in entire patients and separate OAK and POPLAR cohorts (See details in Supporting Information Result 2 and Figures S12 and S13). The OS HR was more strongly correlated with the bTMB‐MSAF score (*R* ^2^ = 0.90) than with the bTMB (*R* ^2^ = 0.68) or with the MSAF (*R* ^2^ = 0.78) (Figure S14). The threshold of <20 gave the optimal clinical relevance, at which the lowest HRs for OS and PFS was identified (Figure S15).

EGFR wild‐type patients with a bTMB‐MSAF score < 20 had improved both OS (HR 0.56, 95% CI, 0.45‐0.69; *P *< .001) and PFS (HR 0.74, 95% CI, 0.61‐0.89; *P* = .0023) when treated with atezolizumab compared with docetaxel (Figure 1C,D). Clinicopathological variables were consistent between treatment arms below the bTMB‐MSAF score < 20 cut‐point (Table S4). Moreover, the EGFR wild‐type patients who concurrently had PD‐L1 expressing on over 50% tumor cells or over 10% of tumor‐infiltrating immune cells (TC3 or IC3) had the greatest benefits for OS (HR 0.44, 95% CI 0.23‐0.81; *P* = .007) and PFS (HR 0.54, 95% CI 0.32‐0.91; *P* = .019; Figure 1E,F; see details in Supporting Information Result 3, Figures S16 and S17, and Table S5). However, EGFR wild‐type patients with bTMB‐MSAF score ≥ 20 and EGFR mutant patients of any threshold showed no difference in OS or PFS two treatments (Figures S18 and S19).

We finally explored blood‐based mutational landscape associated with ICI efficacy in NSCLC (see details in Supporting Information Result 4, Figures S20‐22, and Table S6). The blood mutation status of TP53, KEAP1, and ATM were associated with OS following ICI and were incorporated with race, sex, histology, Eastern Cooperative Oncology Group Performance Status, sum of the longest diameter, number of metastasis sites, PD‐L1 expression, and treatment response information were used to develop clinicopathologic‐genomic nomogram, which was well predictive of atezolizumab efficacy (AUC = 0.855, 0.813, 0.860 for 1‐, 2‐, 3‐year OS; Figure 2A and Supporting Information Table S9 and had high clinical usefulness (Figure 2B). Full procedure of building and validating the nomogram was described in Supporting Information Result 5, Figures S23‐S36, and Tables S6‐S11.

**FIGURE 2:**
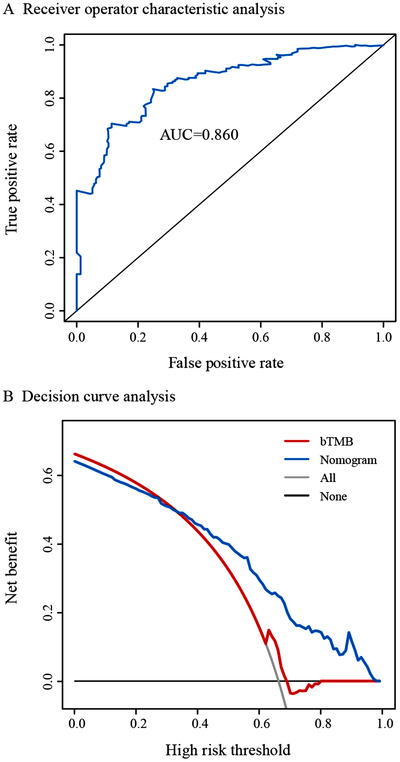
Clinicopathologic‐genomic nomogram to predict the survival of patients undergoing atezolizumab. A, Receiver operating characteristic curves correlating the nomogram with 3‐year overall survival of atezolizumab‐treated patients. B, Decision curve analysis comparing the clinically predictive usefulness between the nomogram and the bTMB

Based on the findings above, we recommend ICI as preferred treatment for EGFR wild‐type patients with a bTMB‐MSAF score < 20, especially those in the TC3 or IC3 subgroup. Our bTMB‐MSAF score markedly enhances the degree of correlation with OS compared with bTMB (*R* ^2^ = 0.90 vs 0.68), indicating 22% predictive information was additionally provided by adding MSAF.

This study is the first to provide evidence on the use of blood‐based genomic alterations as predictors of ICI efficacy, and we suggest KEAP1, TP53, and ATM be screened for mutations. The mechanisms for TP53 and KEAP1 mutations might be analogous with their functions in tissues^1^, ^6^ and the clinical relevance of ATM mutation might be explained by the its role in driving microsatellite instability.^7^ More molecular signature such as epigenomics using lncRNAs has been identified as predictor of cancer ICI efficacy.^8^ A main limitation of this study was that due to a lack of available data, we were unable to evaluate predictive ability of multi‐omics biomarkers.

In conclusion, a novel bTMB‐MSAF algorithm was established by adding MSAF to bTMB, which could precisely identify NSCLC subsets deriving OS and PFS benefits from atezolizumab over docetaxel, and could synergize with PD‐L1 expression. We suggest that considering blood oncogenic alterations and clinicopathological to establish the blood‐based genomic nomogram could aid in selecting candidates to receive ICI.

## AUTHOR CONTRIBUTIONS

Y.Y.Y., Y.J.C., A.L.L., Q.Y.O., X.D.T., Z.H.L., H.H., and H.R.Y. jointly designed the study. Y.Y.Y., Y.J.C., A.L.L., and Q.Y.O. drafted of the manuscript. X.D.T., Z.H.L., H.H., and H.R.Y. provided supervision. All authors contributed to the data collection, data analysis and interpretation, manuscript revision, and approval of the final version.

## FUNDING INFORMATION

This study was supported by grants from the National Science and Technology Major Project (2020ZX09201021), the Medical artificial intelligence project of Sun Yat‐Sen Memorial Hospital (YXRGZN201902), the National Natural Science Foundation of China (81572596, 81972471, U1601223), the Natural Science Foundation of Guangdong Province (2017A030313828), the Guangzhou Science and Technology Major Program (201704020131), the Guangdong Science and Technology Department (2017B030314026), the Special Funds for the Cultivation of Guangdong College Students’ Scientific and Technological Innovation (pdjh2019a0212), National Students’ Innovation and Entrepreneurship training program (201910571001), and Guangdong Medical University College Students’ Innovation Experiment Project (ZZZF001).

## AVAILABILITY OF DATA AND MATERIALS

The datasets generated during and/or analyzed during the current study are available from the corresponding author on reasonable request (yaoherui@mail.sysu.edu.cn).

## ETHICS APPROVAL AND CONSENT TO PARTICIPATE

The study protocol was approved by the ethics committee of the Sun Yat‐sen Memorial Hospital of Sun Yat‐sen University. The requirement for informed consent of study participants was waived because the human data were obtained from publicly available datasets.

## CONSENT FOR PUBLICATION

Not applicable.

## CONFLICT OF INTEREST

The authors declare that they have no competing interests.

## MEETING PRESENTATION

Preliminary results of this study were presented in part as a mini–oral presentation at the ESMO Asia 2019 Congress; November 22–24, 2019; Singapore.

## Supporting information

Supporting information
