# Supplementary material for: Novel blood‐based tumor mutation algorithm and nomogram predict survival of immune checkpoint inhibitor in non‐small‐cell lung cancer: Results from two multicenter, randomized clinical trials
Source: Clin Transl Med. 2020 Jun 5;10(2):e53. doi: 10.1002/ctm2.53 (PMC7403831; doi:10.1002/ctm2.53)
Supplement: Supplementary file 1 — Supporting information [file CTM2-10-e53-s001.doc]

**Supplement**

**Supplemental Methods**

**Supplemental Result S1**

**Supplemental Result S2**

**Supplemental Result S3**

**Supplemental Result S4**

**Supplemental Result S5**

**Supplemental References**

**Figure S1.** Study flowchart.

**Figure S2.** Progression-free survival analysis stratified by treatment in the whole intention-to-treat patients.

**Figure S3.** Progression-free survival analysis stratified by treatment in EGFR wild-type patients.

**Figure S4.** Survival analysis stratified by treatment in EGFR mutant patients.

**Figure S5.** Subgroup analysis of overall and progression-free survival in EGFR wild-type patients based on various clinicopathological variables.

**Figure S6.** Subgroup analysis of overall and progression-free survival in EGFR mutant patients based on various clinicopathological variables.

**Figure S7.** Survival analysis based on a range of cut-points of the blood-based tumor mutation burden in EGFR wild-type patients.

**Figure S8.** Survival analysis based on a range of cut-points of the ctDNA maximum

somatic allele frequency in EGFR wild-type patients.

**Figure S9.** Survival analysis stratified by ctDNA maximum somatic allele frequency.

**Figure S10.** Survival analysis based on a range of cut-points of the blood-based tumor mutation burden in EGFR mutant patients.

**Figure S11.** Survival analysis based on a range of cut-points of the ctDNA maximum

somatic allele frequency in EGFR mutant patients.

**Figure S12.** Identification of the optimal cut-points for risk stratification in the bTMB-MSAF algorithm.

**Figure S13.** Survival analysis stratified by bTMB-MSAF algorithm.

**Figure S14.** Correlation of the genomic signatures with overall survival in EGFR wild-type patients.

**Figure S15.** Survival analysis based on a range of cut-points of the bTMB-MSAF algorithm in EGFR wild-type patients.

**Figure S16.** Venn diagram of the EGFR wild-type population showing the overlap of patients with bTMB-MSAF algorithm<20 and patients in the TC3 or IC3 group.

**Figure S17.** The distribution of bTMB-MSAF algorithm in the EGFR wild-type patients in different PD-L1 expression subgroups.

**Figure S18.** Survival analysis stratified by treatment in EGFR wild-type patients with a bTMB-MSAF algorithm≥20.

**Figure S19.** Survival analysis based on a range of cut-points of the bTMB-MSAF algorithm in EGFR mutant patients.

**Figure S20.** Characterization of blood-based genomic alterations in advanced non-small-cell lung cancer.

**Figure S21.** Landscape of blood-based genomic alterations in atezolizumab-treated EGFR wild-type or mutant patients.

**Figure S22.** Correlation of genomic mutations with blood-based tumor mutation burden in EGFR wild-type patients.

**Figure S23.** Nomogram A to predict the survival of patients undergoing atezolizumab.

**Figure S24.** Identification of the optimal cut-points for risk stratification in the nomograms.

**Figure S25.** Overall survival analysis based on risk stratification using nomogram A.

**Figure S26.** Calibration curves for the nomogram A model to predict overall survival of atezolizumab-treated patients.

**Figure S27.** Decision curve analysis of nomogram A.

**Figure S28.** Nomogram B to predict the survival of patients undergoing atezolizumab.

**Figure S29.** Overall survival analysis based on risk stratification using the nomogram B.

**Figure S30.** Calibration curves for the nomogram B to predict overall survival of atezolizumab-treated patients.

**Figure S31.** Decision curve analysis for the internal validation of the nomograms B and C.

**Figure S32.** Overall survival analysis stratified by treatment response in the combined population of the POPLAR and OAK cohorts.

**Figure S33.** Nomogram C to predict the survival of patients undergoing atezolizumab.

**Figure S34.** Overall survival analysis based on risk stratification using the nomogram C.

**Figure S35.** Overall survival analysis based on risk stratification using nomogram C in EGFR wild-type patients with a bTMB-MSAF algorithm <20 or ≥20.

**Figure S36.** Calibration curves for the nomogram C to predict overall survival of atezolizumab-treated patients.

**Table S1.** Clinical characteristics of EGFR wild-type patients in the OAK and POPLAR trials.

**Table S2.** Clinical characteristics of EGFR mutant patients in the OAK and POPLAR trials.

**Table S3.** Clinical characteristics of the EGFR wild-type and EGFR mutant subgroups across treatment arms.

**Table S4.** Clinical characteristics of EGFR wild-type patients below the bTMB-MSAF algorithm<20 cut-point.

**Table S5.** Summary of overall survival analyses of overlapping subsets between bTMB-MSAF algorithm and PD-L1 expression stratified by EGFR mutation status.

**Table S6.** Association of clinicopathological factors with survival of atezolizumab-treated patients.

**Table S7.** Summary of overall survival analysis of atezolizumab-treated patients with oncogenic driver mutation versus patients with corresponding wild-type tumor.

**Table S8.** Summary of Concordance-index of the predictors.

**Table S9.** Summary of receiver operator characteristics analyses in the nomograms.

**Table S10**. Concordance-index for the internal validation of nomograms B and C.

**Table S11.** Summary of receiver operator characteristics analyses in the internal validation of nomograms B and C.

**Supplemental Methods**

***Study design***

We performed a registry-based pooled analysis of intention-to-treat individual patient data based on 1,137 patients from two multicenter, open-label, randomized controlled trials phase II POPLAR (287 patients; NCT01903993, May 8, 2015, data cutoff) and phase III OAK (850 patients; NCT02008227, July 7, 2016, data cutoff) [1,2], according to the PRISMA-IPD statement, CONSORT statement, and TRIPOD guideline. Patient characteristics were well balanced between the atezolizumab arm (OAK, *N*=425; POPLAR, *N*=144) and the docetaxel arm (OAK, *N*=425; POPLAR, *N*=143) in each of EGFR wild-type and mutant patients (Tables S1-S3). The study protocol was approved by the ethics committee of the Sun Yat-sen Memorial Hospital of Sun Yat-sen University.

***Participants***

Treatment and characteristics of patients have been previously reported [1,2]. In brief, patients were randomly assigned (1:1) to intravenously receive either atezolizumab 1200 mg or docetaxel 75 mg/m² every 3 weeks. Eligible patients were at least 18 years of age; had histologically or cytologically documented advanced or metastatic (stage IIIB or IV) NSCLC with squamous or non-squamous histology; had measurable disease per Response Evaluation Criteria in Solid Tumors (RECIST) version 1.1; had previously once or twice received platinum-based combination chemotherapy; and had Eastern Cooperative Oncology Group Performance Status (ECOG-PS) 0 or 1. Previous tyrosine kinase inhibitor therapy was additionally required for patients with EGFR or ALK mutations in the OAK trial. Patients were excluded if had they previously received CD137 agonists, CTLA4 inhibitors, or PD-L1/PD-1 inhibitors and if they had untreated or active brain metastases.

PD-L1 expression was assessed centrally and prospectively in tumor specimens before enrollment. TC0 and IC0 indicates that less than 1% of tumor cells and less than 1% of tumor infiltrating immune cells expressed PD-L1; TC0/1/2 and IC0/1/2 indicates that less than 50% of tumor cells and less than 10% of tumor infiltrating immune cells expressed PD-L1; TC1/2/3 or IC1/2/3 indicates that over 1% of tumor cells or over 1% of tumor infiltrating immune cells expressed PD-L1; TC3 or IC3 indicates that over 50% of tumor cells or over 10% of tumor infiltrating immune cells expressed PD-L1 [1].

The blood-based tumor mutation burden (bTMB) was defined as the somatic mutations in plasma. All nonsynonymous mutations including missense, nonsense, and splice site were equally considered. The ctDNA maximum somatic allele frequency (MSAF) was defined as the highest allele frequency of the detected somatic variants, which was also an estimate of the tumor content. Assays of the bTMB and the MSAF have been described in detail in a previous publication [3]. In brief, blood samples were received at baseline and was stored as frozen plasma at –80 °C for further analyses. Plasma was purified through thawing and centrifugation and then was utilized for cell-free DNA extraction (cfDNA). cfDNA fragments between 100 and 700 base pairs underwent a protocol to realize maximal retention and were used to determine the cfDNA concentration. A total of 20–100ng purified cfDNA was used for library construction, which aimed to maximize the library yield and complexity. Both ends of each input duplex of cfDNA fragment was ligated with a set of specific, designed fragment-level indexed adaptors. Ligated sequencing libraries underwent PCR amplification, and samples yielding 500–2,000ng of sequencing library were next processed with hybrid capture. bTMB was determined using the FDA-approved FoundationOne CDx NGS TMB assay described in Frampton et al [4] and a 394-gene bait set described in Chalmers et al [5]. After analyzing SNVs with≥0.5% frequency and removing germline variants and oncogenic driver mutations, bTMB and the MSAF were finally identified. The accuracy and precision of bTMB and the MSAF was analytically validation.

***Procedures***

We applied a two-stage random effect meta-analysis of individual patient data to compare the efficacy of atezolizumab and docetaxel. After removing ALK mutant patients, prespecified analyses were performed on the basis of stratification by EGFR mutation status and subgroup analyses by bTMB, sex, age, race, ECOG-PS, histology, smoking status, prior courses of chemotherapy, sum of the longest tumor diameter, number of metastasis sites, KRAS mutation status, and PD-L1 expression. Patients in each of EGFR wild-type and mutant groups were further analyzed by the bTMB, MSAF, and bTMB-MSAF algorithm according to a range of cut-points. We used the nomogram approach to provide clinically applicable tools that incorporated blood-based mutated genes and clinicopathological variables to predict survival probability of patients in the atezolizumab group at enrollment and further added treatment response data to refine the survival estimates after initiation of atezolizumab treatment. We assessed the predictive effects of the bTMB, MSAF and clinicopathologic-genomic nomogram for atezolizumab based on Cox regression model. The overview of study design is shown in Figure S1. The primary endpoint was OS, and the secondary endpoint was PFS.

***Statistical analysis***

Median PFS and OS and survival curves were estimated by the Kaplan-Meier method and the treatment effects were assessed with the log-rank test. Time-to-event data were calculated by pooling hazard ratios (HRs)and corresponding 95% confidence interval (CIs) with the Cox regression model, and the pooled dichotomous data estimates were expressed as relative risks with their 95% CIs. We compared categorical variable using χ2 or Fisher’s exact test and compared continuous variable using t test or Wilcoxon rank sum test in two-group comparison. The coefficient of determination (R^2^) of a linear regression model was used to estimate potential correlations. The optimal cutoff value for continuous variable was generated by the R package survminer. The frequencies of altered genes were visualized through plotting the oncoprint with the R package ComplexHeatmap. We used the rms package of R to build nomograms. Predictors were determined by univariable Cox regression analysis. The performance of the nomogram was graphically and quantitively assessed by calibration plots and the concordance index (C-index), respectively. Using the R package survivalROC, we generated receiver operator characteristic (ROC) curves and calculated the areas under the curves to evaluate the predictive accuracy of the signature and to assess its sensitivity and specificity [6]. Additionally, decision curve analysis (DCA) was performed to assess the clinical utility of the prediction model by quantifying the net benefits when different threshold probabilities were considered [7]. For all analyses, *P*-values less than 0.05 were considered statistically significant. Statistical analyses were performed using R (version 3.6.0) and SPSS (version 22.0).

**Supplemental Result S1**

The OS benefit of atezolizumab over docetaxel in EGFR wild-type patients were generally consistent across subgroups, including PD-L1 expression subgroups, with the greatest improvement in the TC3 or IC3 subgroup (HR 0.45, 95% CI 0.28–0.71), followed by the TC0 and IC0 subgroup (HR 0.64, 95% CI 0.49–0.84), TC1/2/3 or IC1/2/3 subgroup (HR 0.72, 95% CI 0.57–0.92), and TC0/1/2 and IC0/1/2 subgroup (HR 0.74, 95% CI 0.61–0.90). Consistent results were also observed in squamous patients (HR 0.68, 95% CI 0.52–0.89) non-squamous patients (HR 0.66, 95% CI 0.55–0.80), patients who never smoked (HR 0.59, 95% CI 0.38–0.92), current or former smokers (HR 0.68, 95% CI 0.58–0.80), and KRAS-negative patients (HR 0.64, 95% CI 0.53–0.77). With respect to PFS, a significant improvement was only observed in the TC3 or IC3 patients (HR 0.67, 95% CI 0.45–0.99), squamous patients (HR 0.73, 95% CI 0.56–0.95), current or previous smoking status (HR 0.84, 95% CI 0.72–0.98), and KRAS-negative patients (HR 0.83, 95% CI 0.70–0.99). Full results of the subgroup analyses are presented in Figure S5. No significant difference in OS or PFS was observed across any clinicopathological subgroup of EGFR mutant patients (Figure S6).

**Supplemental Result S2**

We did a Cox regression analysis of PFS based on the MSAF and bTMB data of the whole ALK wild-type intention-to-treat patients, which generated weighted coefficients for bTMB (−0.004341) and for MSAF (2.626817). We integrated the bTMB and MSAF to construct a novel bTMB-MSAF algorithm by using these coefficients, which was calculated as follows: Algorithm = (−0.004341 × bTMB + 2.626817 × MSAF) ×100. The algorithm was multiplied by 100, which aimed to further enhance the applicability and generality for clinical decision making.

We used the survminer package to obtain an optimal cutoff value (17.02) to separate EGFR wild-type patients into the low-score and high-score groups (Figure S12). Patients with low bTMB-MSAF algorithms were associated with significantly better OS (HR 0.57, 95% CI 0.48–0.68; *P*<0.001) and PFS (HR 0.70, 95% CI 0.60–0.82; *P*<0.001) compared with patients with high bTMB-MSAF algorithms (Figure S13A-B). Considering that there is no other separate NSCLC immunotherapy cohort having available blood-based genomic data, we conducted subsequent internal validation using ALK wild-type patients in OAK cohort and POPLAR cohort separately to substantiate the stability of the bTMB-MSAF algorithm. In the OAK group, patients with low bTMB-MSAF algorithms significantly improved OS (HR 0.56, 95% CI 0.46–0.68; *P*<0.001) and PFS (HR 0.69, 95% CI 0.57–0.82; *P*<0.001) compared with patients with high bTMB-MSAF algorithms (Figure S13C-D). Consistently, the significant differences in OS (HR 0.61, 95% CI 0.44–0.85; *P*=0.003) and PFS (HR 0.73, 95% CI 0.54–0.99; *P*=0.045) were also confirmed in the POPLAR group (Figure S13E-F).

**Supplemental Result S3**

Furthermore, we conducted subgroup analysis by PD-L1 expression. Among the 508 EGFR wild-type patients with a bTMB-MSAF algorithm<20, only a small proportion (70 [13.8%]; Figure S16) concurrently had a high PD-L1 expression that PD-L1 expressed on over 50% tumor cells (TCs) or over 10% of tumor-infiltrating immune cells (ICs) (TC3 or IC3). Additionally, the distribution of the bTMB-MSAF algorithm was similar between the TC3 or IC3 group and the TC0/1/2 and IC0/1/2 group (less than 50% of TCs and less than 10% of ICs expressed PD-L1) (*P*=0.6; Figure S17). These findings demonstrated the predictive value of the bTMB-MSAF algorithm was independent of PD-L1 expression.

Moreover, at bTMB-MSAF algorithm<20, OS was significantly better with atezolizumab than with docetaxel across all categories of PD-L1 expression (Table S5). Patients scored as TC3 or IC3 gained the greatest benefits for OS (HR 0.44, 95% CI 0.23–0.81; *P*=0.007) and PFS (HR 0.54, 95% CI 0.32–0.91; *P*=0.019). However, none of the tested overlapping subsets between bTMB-MSAF algorithm and PD-L1 expression could classify atezolizumab- versus docetaxel-treated EGFR mutant patients into groups with different PFS or OS (Table S5).

**Supplemental Result S4**

A landscape of 20 most frequently mutated genes was identified in the whole intention-to-treat patients (Figure S20A), among which the 20 most frequently mutated genes that occurred in ≥10% of patients included TP53 (50%), LRP1B (31%), DNMT3A (23%), SPTA1 (18%), FAT3 (18%), KEAP1 (14%), NF1 (13%), MLL2 (12%), STAG2 (12%), FAT1 (11%), TSC1 (11%), MLL3 (11%), ATM (10%), SMARCA4 (9%), EPHA6 (9%), PTPRD (9%), KRAS (9%), TET2 (8%), and PREX2 (8%). These genes displayed similar mutation pattern in the atezolizumab-treated patients (Figure S20B), among which EGFR wild-type patients showed a higher frequency of mutations in DNMT3A (22% versus 14%), NF1 (12% versus 7%), and TSC1 (10% versus 5%) compared with EGFR mutant patients, but a lower frequency of mutations in TP53 (50% versus 57%) and FAT1 (14% versus 19%) (Figure S21). Oncogenes whose mutation status was significantly associated with OS benefits of atezolizumab included TP53 (*P*=0.002), KEAP1 (*P*<0.001), and ATM (*P*=0.038) (Figure S20C-E and Table S6). Additionally, the bTMB was higher among atezolizumab-treated patients with TP53 (*P*<0.001), KEAP1 (*P*<0.001), and ATM (*P*=0.014) mutations compared with patients with corresponding wild-type tumors (Figure S22).

**Supplemental Result S5**

A clinicopathologic-genomic nomogram A was constructed in the OAK cohort to predict individual survival of atezolizumab-treated patients (Figure S23), taking into consideration race, sex, histology, Eastern Cooperative Oncology Group Performance Status, sum of the longest diameter, number of metastasis sites, and the mutation status of TP53, KEAP1, and ATM (Tables S6-S7). Using the optimal cut-point (4.70) for risk stratification in nomogram A (Figure S24A), a significant difference in OS was identified between patients classified as high risk and low risk in the OAK cohort (HR 0.37, 95% CI 0.28–0.49; *P*<0.001), the POPLAR cohort (HR 0.37, 95% CI 0.18–0.66; *P*<0.001), and two cohorts combined (HR 0.42, 95% CI 0.33–0.54; *P*<0.001) (Figure S25). The calibration plots for 1- or 2-year OS were predicted well in the OAK cohort (C-index, 0.650), the POPLAR cohort (C-index, 0.669), and two cohorts combined (C-index, 0.646) (Figure S26 and Table S8). The receiver-operator characteristic analysis indicated the effectiveness of nomogram A in predicting 1-, 2-, and 3-year OS in the OAK cohort (AUC=0.694, 0.721, 0.733, respectively), the POPLAR cohort (AUC=0.693, 0.726, 0.711, respectively), and two cohorts combined (AUC=0.684, 0.696, 0.714, respectively) (Table S9). Nomogram A displayed better clinical predictive usefulness than bTMB in the OAK cohort, the POPLAR cohort, and two cohorts combined in decision curve analysis (DCA) (Figures S27).

Since PD-L1 expression was an important immune-related biomarker and was significantly associated with OS with atezolizumab (Table S6); we therefore added PD-L1 expression to nomogram A in the OAK cohort, resulting in nomogram B (Figure S28), which showed an improved OS for patients with low-risk scores versus high-risk scores with the optimal cut-point (3.35) (HR 0.38, 95% CI 0.29–0.50; *P*<0.001; Figures S24B and S29A), and displayed a tendency towards increasing C-index (0.651; Figure S30A and Table S8), AUC value (0.701, 0.738, 0.756 for 1-, 2-, 3-year OS, respectively; Table S9) and clinical predictive usefulness.

Considering POPLAR cohort did not have available PD-L1 information for the validation of nomogram B, we randomly assigned (1:1) atezolizumab-treated patients in OAK cohort into validation cohort 1 and validation cohort 2 to performed internal validation for nomogram B. We identified a significant difference in OS between patients classified as low risk versus high risk in the validation cohort 1 (HR 0.35, 95% CI 0.24–0.51; *P*<0.001; Figure S29B) and the validation cohort 2 (HR 0.42, 95% CI 0.28–0.64; *P*<0.001; Figure S29C). The calibration plots for 2-year OS were predicted well in the validation cohort 1 (C-index, 0.670) and the validation cohort 2 (C-index, 0.630) (Figure S30B-C and Table S10). The ROC analysis indicated the effectiveness of nomogram B in predicting 1-, 2-, and 3-year OS in the validation cohort 1 (AUC= 0.736, 0.728, 0.778, respectively) and the validation cohort 2 (AUC=0.668, 0.746, 0.746, respectively) (Table S11). Nomogram B displayed better clinical predictive usefulness than bTMB in the validation cohort 1 and validation cohort 2 in DCA (Figure S31).

In the pooled analysis of atezolizumab-treated patients in the OAK and POPLAR trials, a significant difference in OS was observed in patients with different treatment response status (complete response versus partial response versus stable disease versus progressive disease; HR 0.34, 95% CI 0.26–0.44; *P*=0.001; Figure S32). Given powerful predictive value of treatment response, we built a nomogram C in the OAK cohort by further adding treatment response data to nomogram B (Figure S33). Patients classified as low risk versus high risk had markedly longer OS using the optimal cut-point (5.79) (HR 0.19, 95% CI 0.13–0.29; *P*<0.001; Figures S24C and S34A), a finding that was consistently pronounced in EGFR wild-type patients with a bTMB-MSAF algorithm<20 (HR 0.20, 95% CI 0.13–0.32; *P*<0.001) or≥20 (HR 0.21, 95% CI 0.10–0.48; *P*<0.001) (Figure S35). Nomogram C displayed a tendency towards increasing C-index (0.651; Figure S36A and Table S8)

Likewise, we performed internal validation for nomogram C. We identified a significant difference in OS between patients classified as low risk versus high risk in the validation cohort 1 (HR 0.27, 95% CI 0.16–0.45; *P*<0.001; Figure S34B) and the validation cohort 2 (HR 0.13, 95% CI 0.07–0.24; *P*<0.001; Figure S34C). The calibration plots for 2-year OS were predicted well in the validation cohort 1 (C-index, 0.724) and the validation cohort 2 (C-index, 0.771) (Figure S36B-C and Table S10). The ROC analysis indicated the effectiveness of nomogram C in predicting 1-, 2-, and 3-year OS in the validation cohort 1 (AUC= 0.835, 0.757, 0.869, respectively) and the validation cohort 2 (AUC=0.882, 0.874, 0.874, respectively) (Table S11). Nomogram C displayed better clinical predictive usefulness than bTMB and nomogram B in the validation cohort 1 and validation cohort 2 in DCA (Figure S31).

**Supplemental References**

1. [Fehrenbacher L](https://www.ncbi.nlm.nih.gov/pubmed/?term=Fehrenbacher%20L%5BAuthor%5D&cauthor=true&cauthor_uid=26970723), [Spira A](https://www.ncbi.nlm.nih.gov/pubmed/?term=Spira%20A%5BAuthor%5D&cauthor=true&cauthor_uid=26970723), [Ballinger M](https://www.ncbi.nlm.nih.gov/pubmed/?term=Ballinger%20M%5BAuthor%5D&cauthor=true&cauthor_uid=26970723) et al. Atezolizumab versus docetaxel for patients with previously treated non-small-cell lung cancer (POPLAR): a multicentre, open-label, phase 2 randomised controlled trial. Lancet 2016;387(10030):1837-46.
2. [Rittmeyer A](https://www.ncbi.nlm.nih.gov/pubmed/?term=Rittmeyer%20A%5BAuthor%5D&cauthor=true&cauthor_uid=27979383), [Barlesi F](https://www.ncbi.nlm.nih.gov/pubmed/?term=Barlesi%20F%5BAuthor%5D&cauthor=true&cauthor_uid=27979383), [Waterkamp D](https://www.ncbi.nlm.nih.gov/pubmed/?term=Waterkamp%20D%5BAuthor%5D&cauthor=true&cauthor_uid=27979383) et al. Atezolizumab versus docetaxel in patients with previously treated non-small-cell lung cancer (OAK): a phase 3, open-label, multicentre randomised controlled trial. Lancet 2017;389(10066):255-265.
3. [Gandara DR](https://www.ncbi.nlm.nih.gov/pubmed/?term=Gandara%20DR%5BAuthor%5D&cauthor=true&cauthor_uid=30082870), [Paul SM](https://www.ncbi.nlm.nih.gov/pubmed/?term=Paul%20SM%5BAuthor%5D&cauthor=true&cauthor_uid=30082870), [Kowanetz M](https://www.ncbi.nlm.nih.gov/pubmed/?term=Kowanetz%20M%5BAuthor%5D&cauthor=true&cauthor_uid=30082870) et al. Blood-based tumor mutational burden as a predictor of clinical benefit in non-small-cell lung cancer patients treated with atezolizumab. [Nat Med](https://www.ncbi.nlm.nih.gov/pubmed/?term=Blood-based+tumor+mutational+burden+as+a+predictor+of+clinical+benef) 2018;24(9):1441-1448.
4. Frampton GM, Fichtenholtz A, Otto GA et al. Development and validation of a clinical cancer genomic profling test based on massively parallel DNA sequencing. Nat Biotechnol 2013;31:1023–1031.
5. Chalmers ZR, Connelly CF, Fabrizio D et al. Analysis of 100,000 human cancer genomes reveals the landscape of tumor mutational burden. Genome Med 2017;9:34.
6. Heagerty PJ, Lumley T, Pepe MS. Time-dependent ROC curves for censored survival data and a diagnostic marker. Biometrics 2000;56:337–44.
7. Kerr KF, Brown MD, Zhu K et al. Assessing the clinical impact of risk prediction models with decision curves: guidance for correct interpretation and appropriate use. J Clin Oncol 2016;34:2534–40.

**Figure S1.** Study flowchart.

**
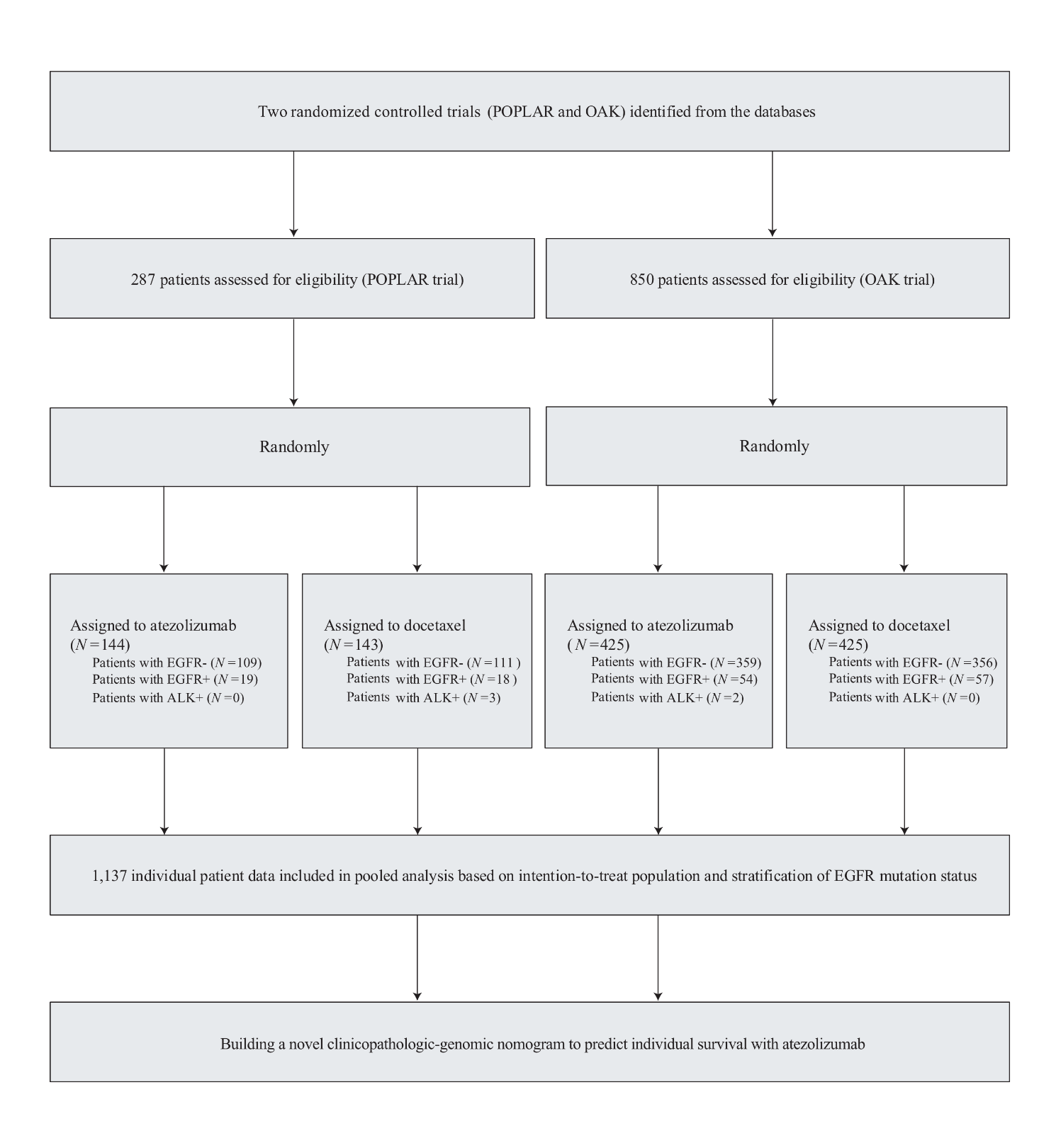
**

**Figure S2.** Progression-free survival analysis stratified by treatment in the whole intention-to-treat patients.


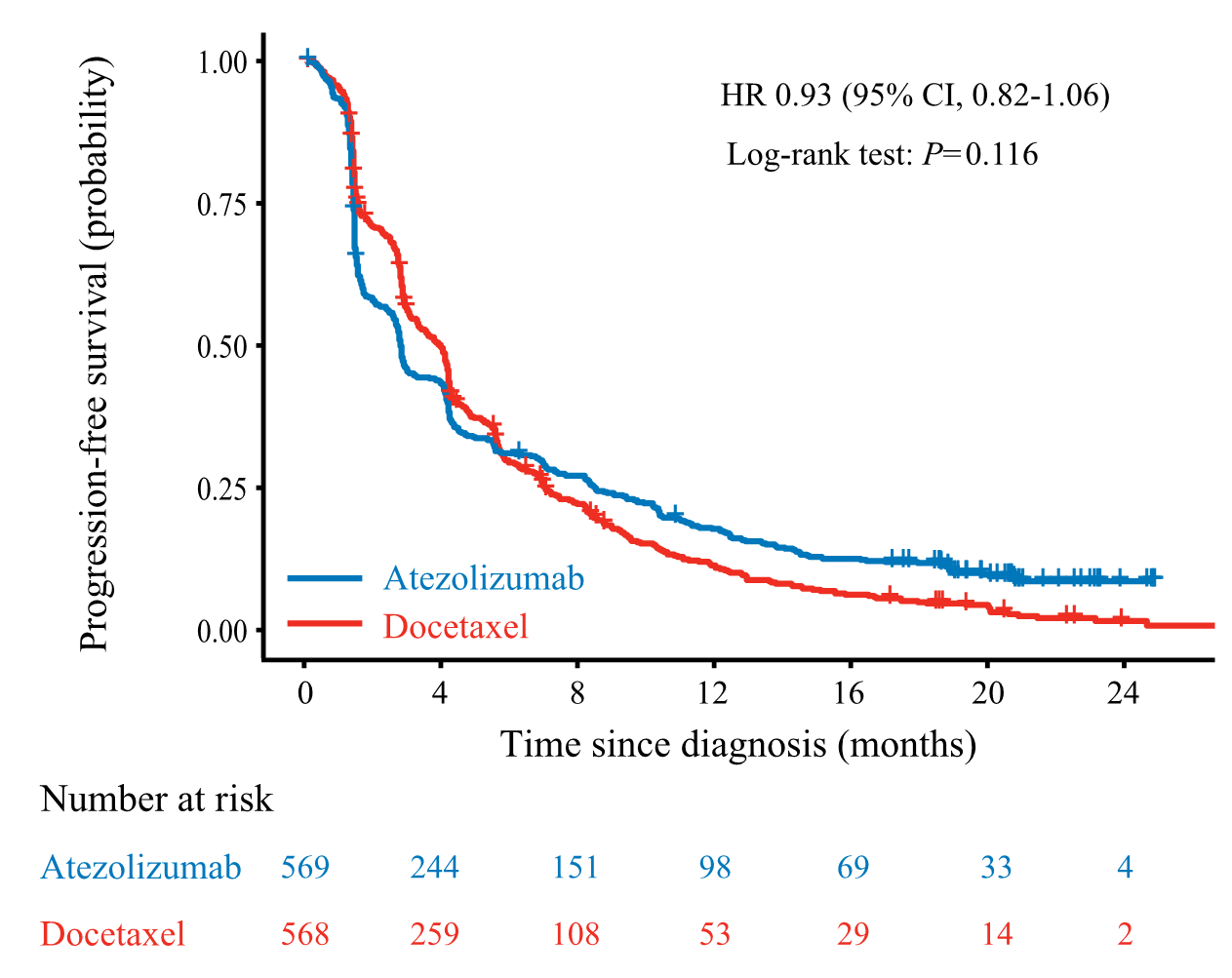


HR, hazard ratio; CI, confidence interval.

**Figure S3.** Progression-free survival analysis stratified by treatment in EGFR wild-type patients.


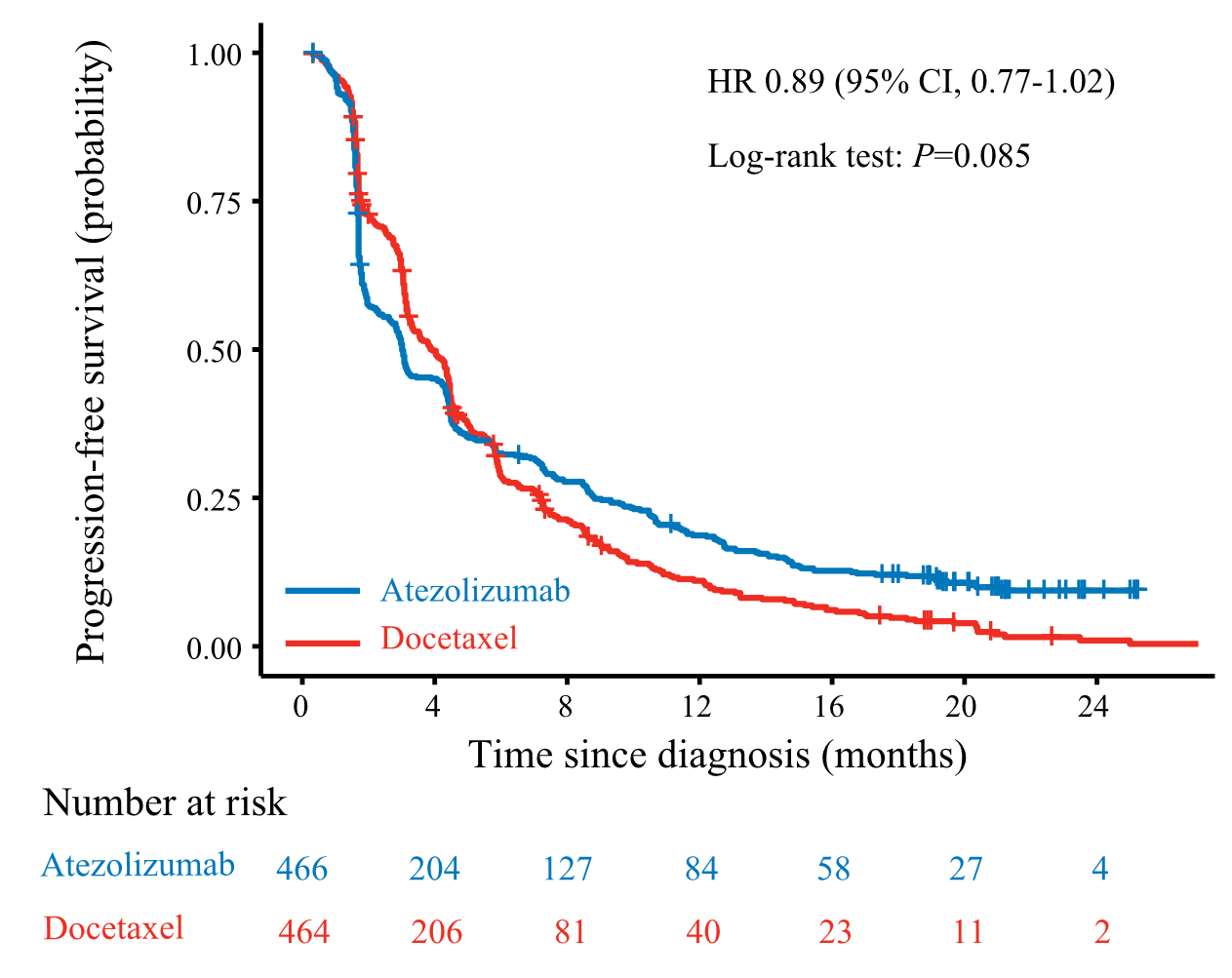


HR, hazard ratio; CI, confidence interval.

**Figure S4.** Survival analysis stratified by treatment in EGFR mutant patients.


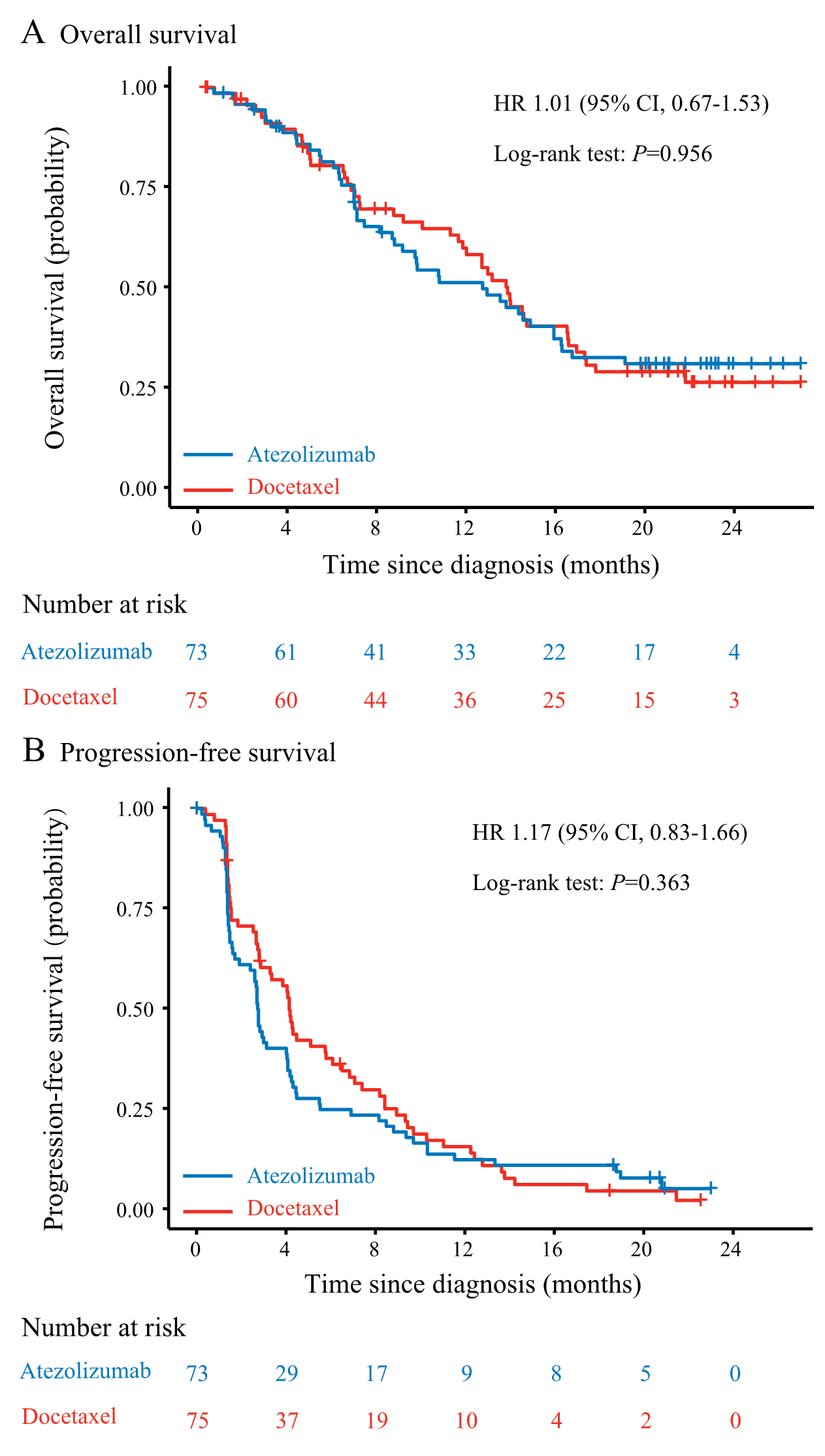


(A) Overall survival. (B) Progression-free survival. HR, hazard ratio; CI, confidence interval

**Figure S5.** Subgroup analysis of overall and progression-free survival in EGFR wild-type patients based on various clinicopathological variables.


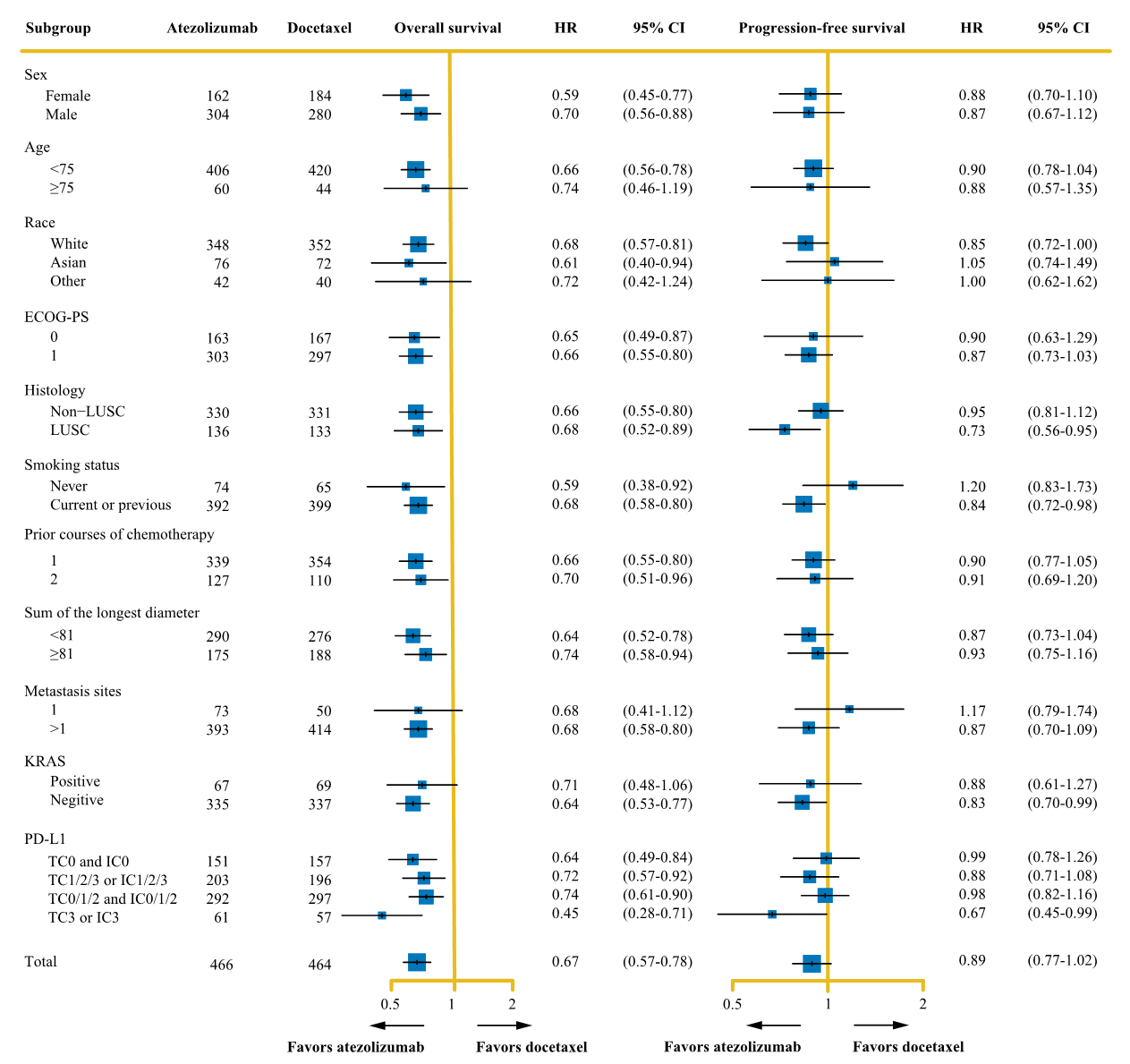


HR, hazard ratio; CI, confidence interval; ECOG-PS, Eastern Cooperative Oncology Group Performance Status; LUSC, lung squamous cell carcinoma; PD-L1, programmed cell death ligand 1.

**Figure S6.** Subgroup analysis of overall and progression-free survival in EGFR mutant patients based on various clinicopathological variables.

**
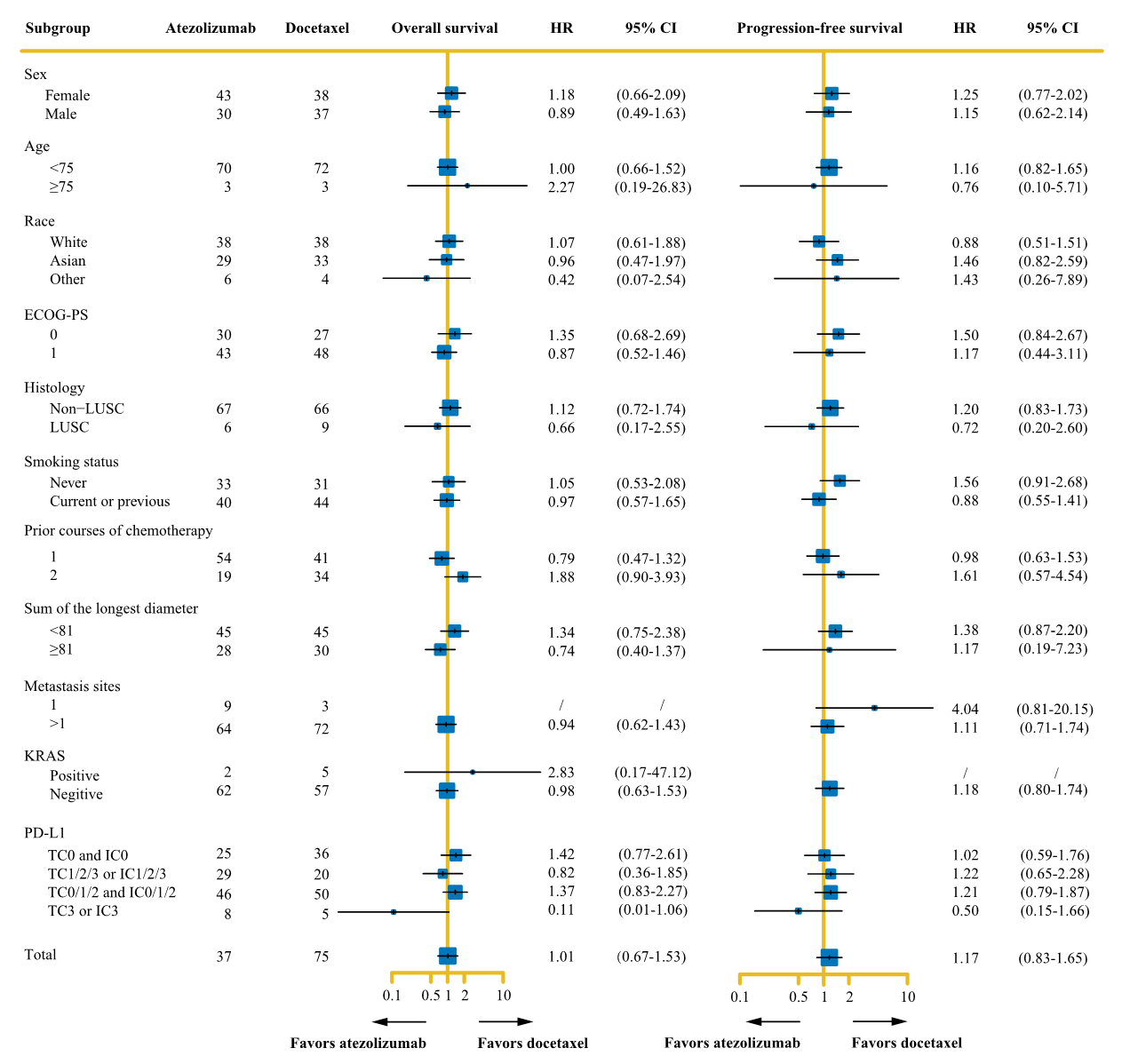
**

HR, hazard ratio; CI, confidence interval; ECOG-PS, Eastern Cooperative Oncology Group Performance Status; LUSC, lung squamous cell carcinoma; PD-L1, programmed cell death ligand 1.

**Figure S7.** Survival analysis based on a range of cut-points of the blood-based tumor mutation burden in EGFR wild-type patients.


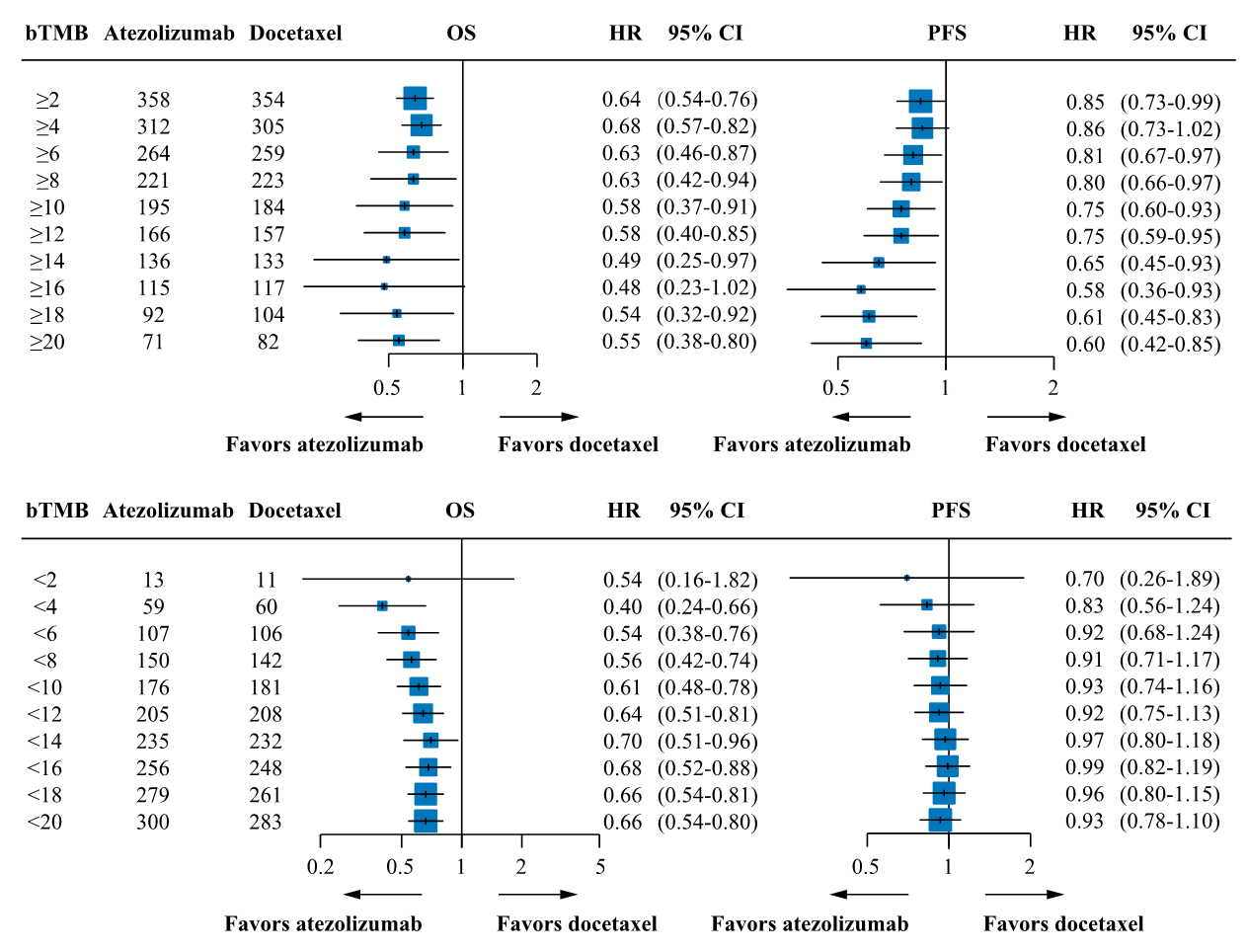


bTMB, blood-based tumor mutation burden; OS, overall survival; PFS, progression-free survival; HR, hazard ratio; CI, confidence interval.

**Figure S8.** Survival analysis based on a range of cut-points of the ctDNA maximum

somatic allele frequency in EGFR wild-type patients.

**
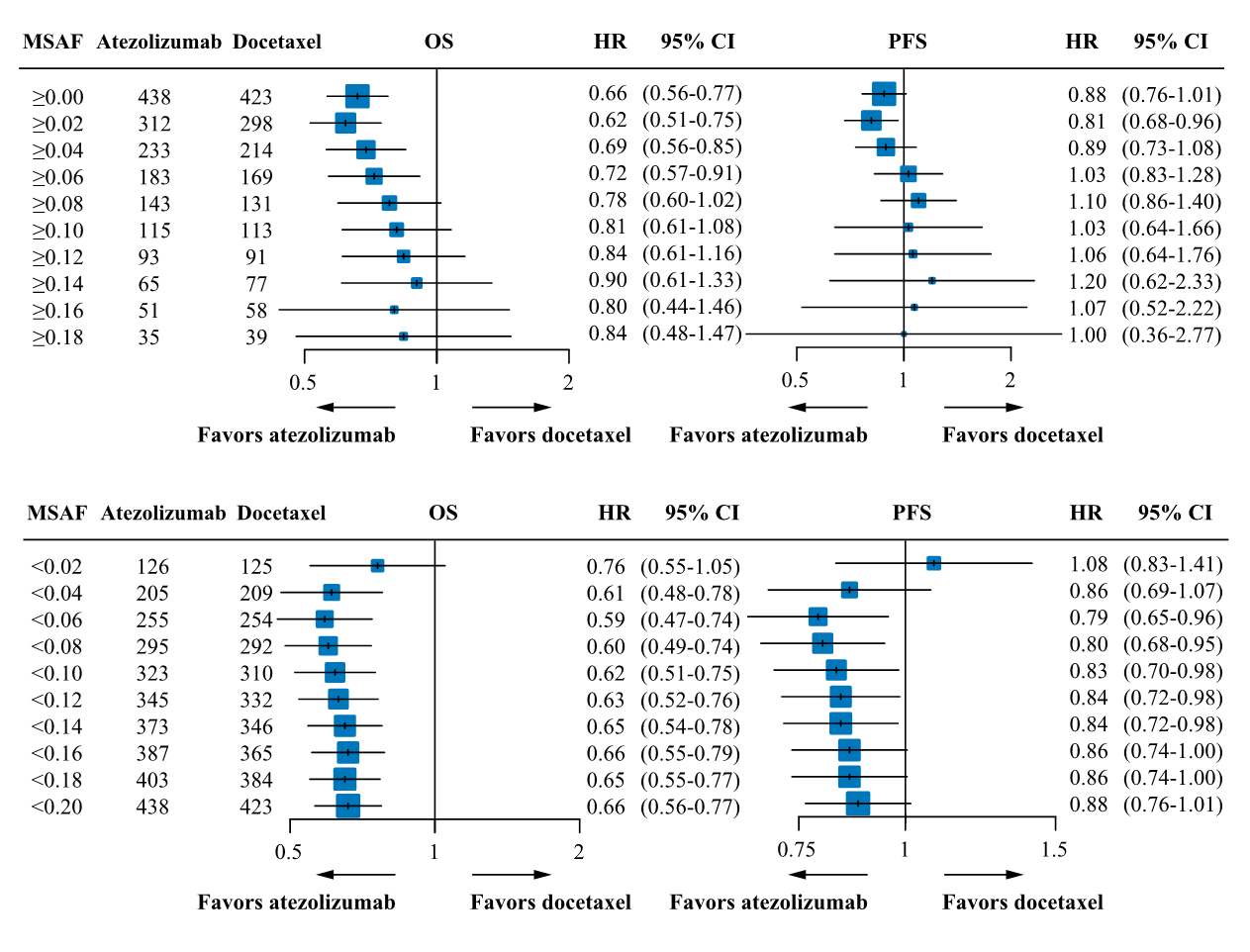
**

OS, overall survival; PFS, progression-free survival; HR, hazard ratio; CI, confidence interval. MSAF indicated ctDNA maximum somatic allele frequency.

**Figure S9.** Survival analysis stratified by ctDNA maximum somatic allele frequency.


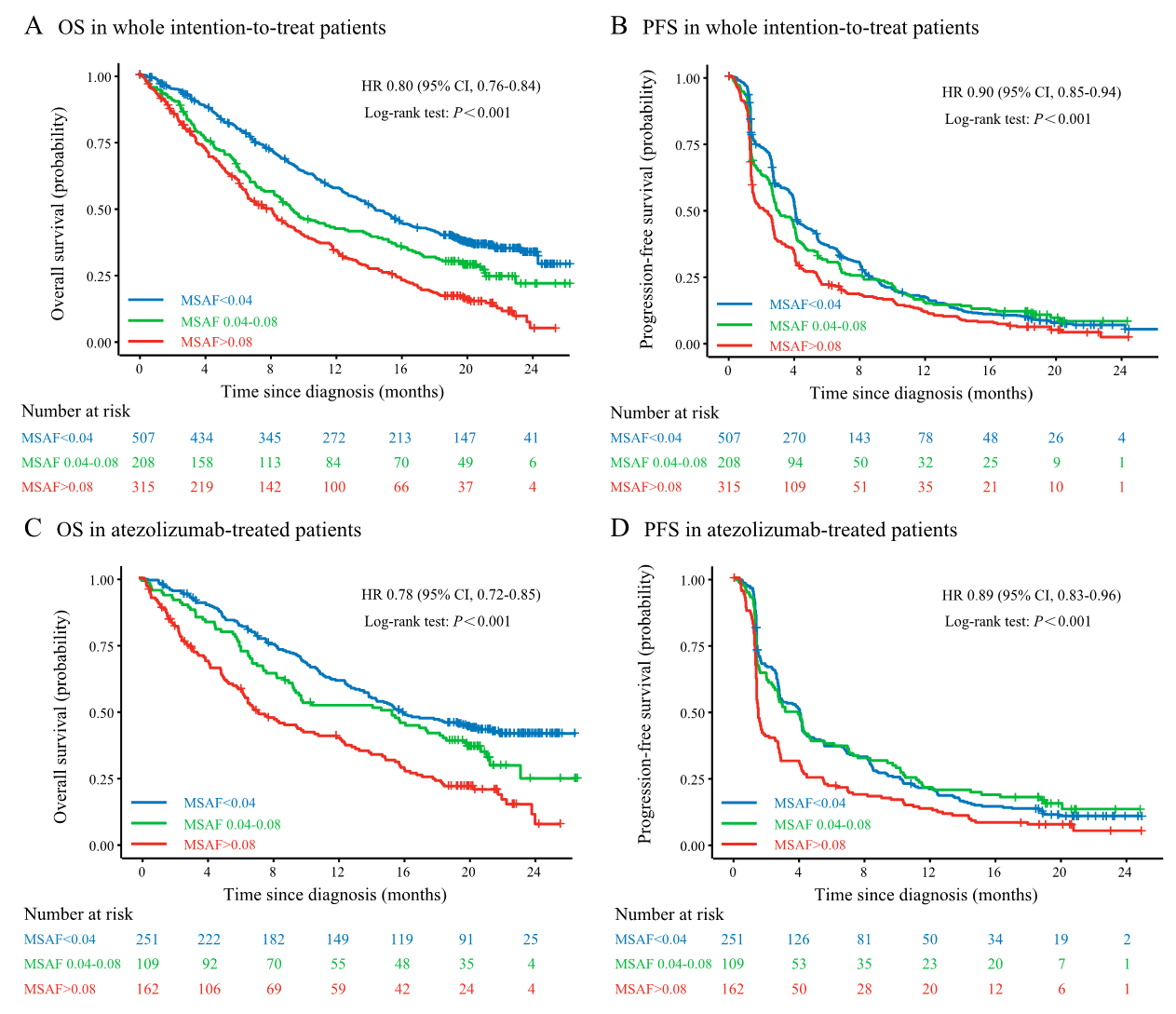


(A) and (B) Overall survival and progression-free survival in the whole intention-to-treat patients, respectively. (C) and (D) Overall survival and progression-free survival in the atezolizumab-treated patients, respectively. OS, overall survival; PFS, progression-free survival; HR, hazard ratio; CI, confidence interval. MSAF indicated ctDNA maximum somatic allele frequency.

**Figure S10.** Survival analysis based on a range of cut-points of the blood-based tumor mutation burden in EGFR mutant patients.


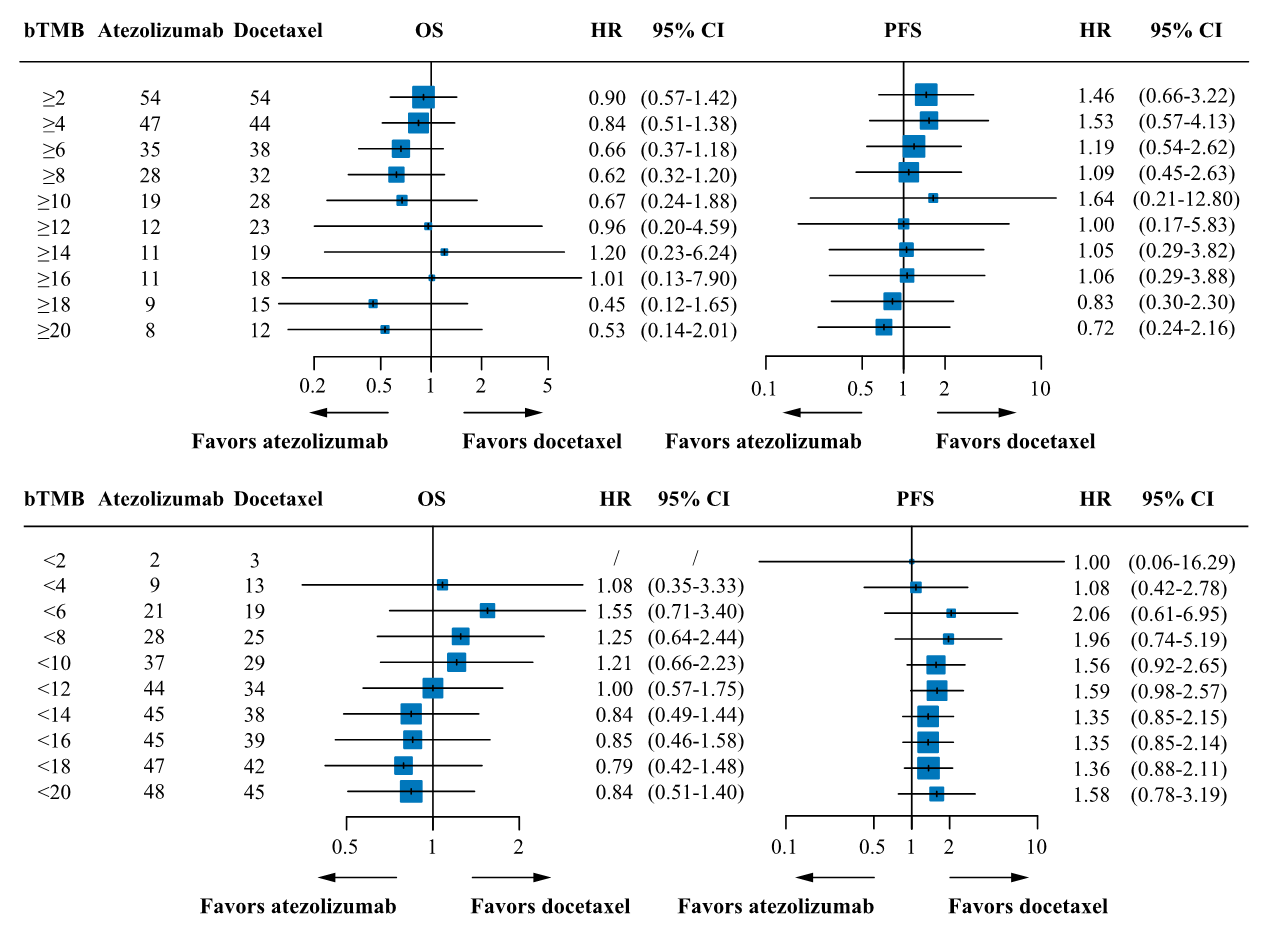


bTMB, blood-based tumor mutation burden. OS, overall survival; PFS, progression-free survival; HR, hazard ratio; CI, confidence interval.

**Figure S11.** Survival analysis based on a range of cut-points of the ctDNA maximum

somatic allele frequency in EGFR mutant patients.

**
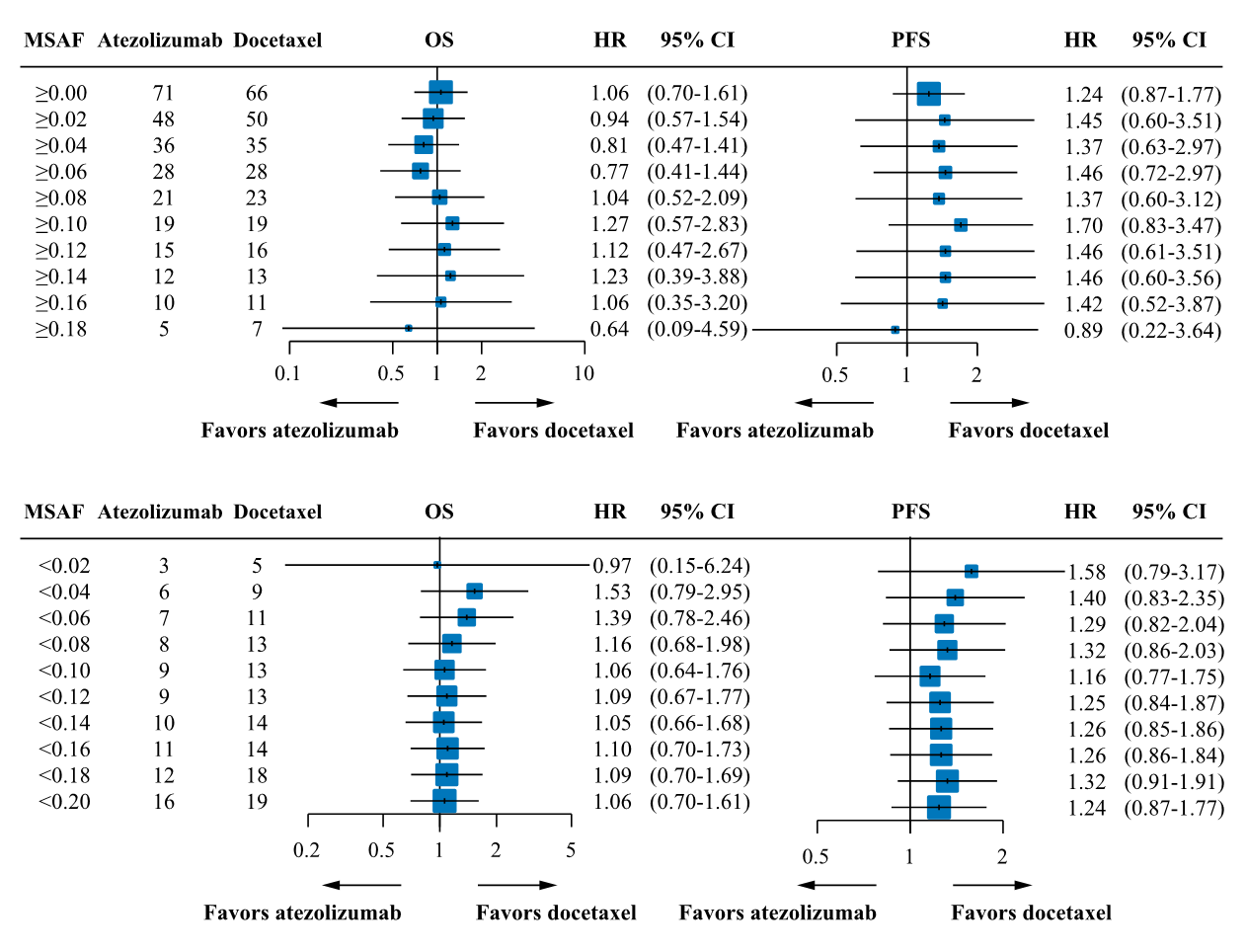
**

OS, overall survival; PFS, progression-free survival; HR, hazard ratio; CI, confidence interval. MSAF indicated ctDNA maximum somatic allele frequency.

**Figure S12.** Identification of the optimal cut-points for risk stratification in the bTMB-MSAF algorithm.

**
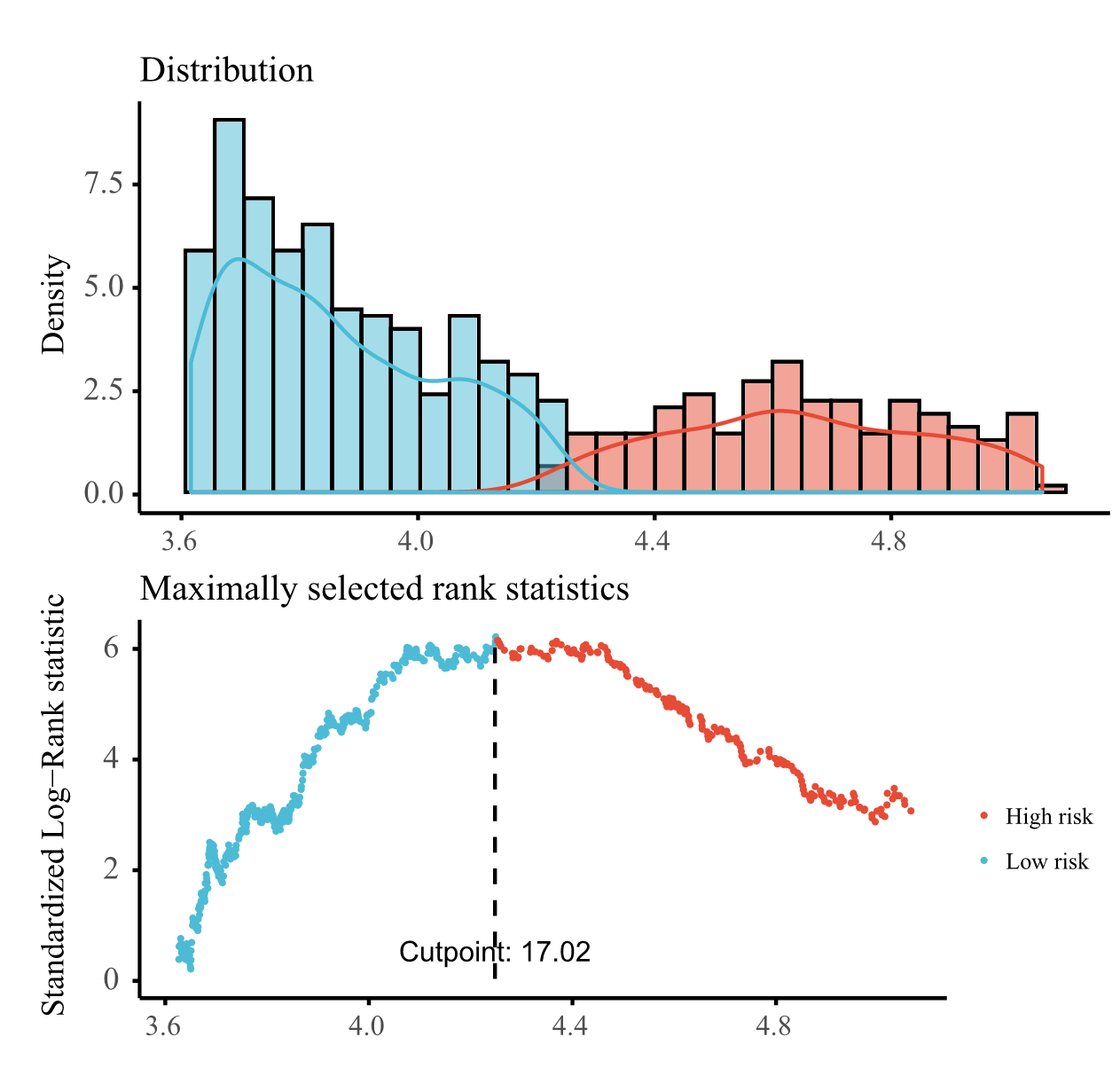
**

OS, overall survival; PFS, progression-free survival; HR, hazard ratio; CI, confidence interval.

**Figure S13.** Survival analysis stratified by bTMB-MSAF algorithm.


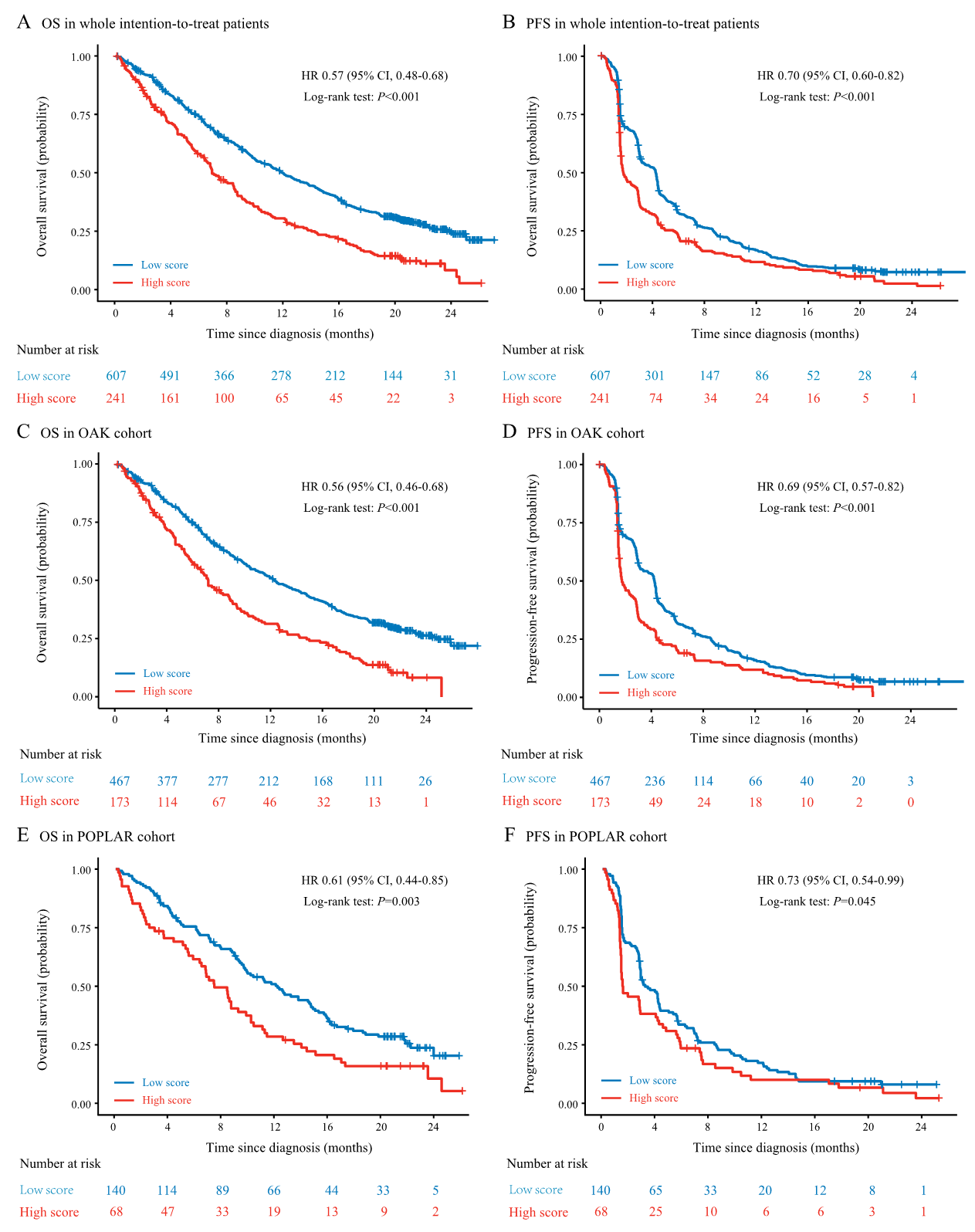


(A) and (B) showed the OS and PFS in whole intention-to-treat patients, respectively. (C) and (D) showed the internal validation of the OS and PFS in OAK cohort, respectively. (E) and (F) showed the internal validation of the OS and PFS in POPLAR cohort, respectively. OS, overall survival; PFS, progression-free survival; HR, hazard ratio; CI, confidence interval.

**Figure S14.** Correlation of the genomic signatures with overall survival in EGFR wild-type patients.


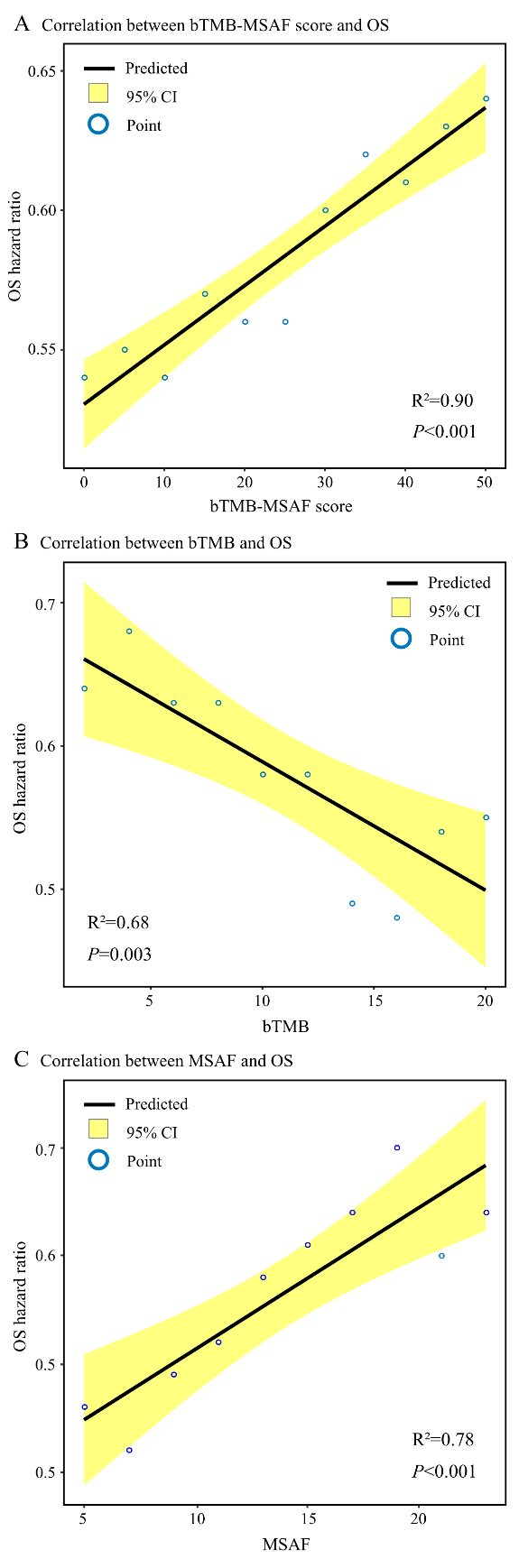


(A) bTMB-MSAF algorithm, (B) Blood-based tumor mutation burden. (C) ctDNA maximum somatic allele frequency. CI, confidence interval; OS, overall survival. bTMB indicates blood-based tumor mutation burden. MSAF indicated ctDNA maximum somatic allele frequency.

**Figure S15.** Survival analysis based on a range of cut-points of the bTMB-MSAF algorithm in EGFR wild-type patients.

**
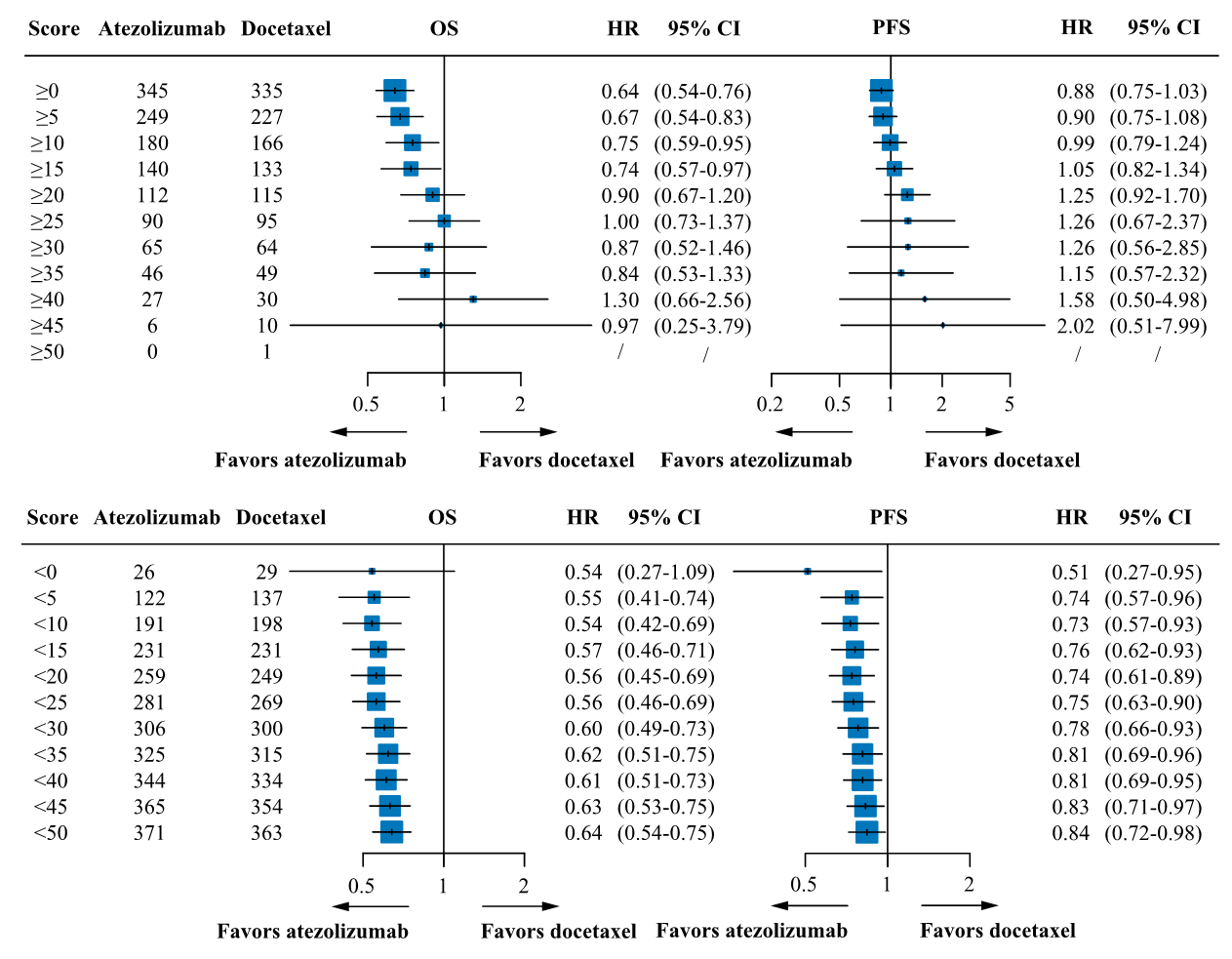
**

Several cut-points could successfully classify patients into groups with consistently distinct OS and PFS benefits of atezolizumab versus docetaxel, including 20, 25, 30, 35, 40, and 45, which showed a monotonical association with increased HRs for OS and PFS. The threshold of <20 gave the optimal clinical relevance, at which the lowest HRs for OS and PFS was identified. OS, overall survival; PFS, progression-free survival; HR, hazard ratio; CI, confidence interval.

**Figure S16.** Venn diagram of the EGFR wild-type population showing the overlap of patients with bTMB-MSAF algorithm<20 and patients in the TC3 or IC3 group.


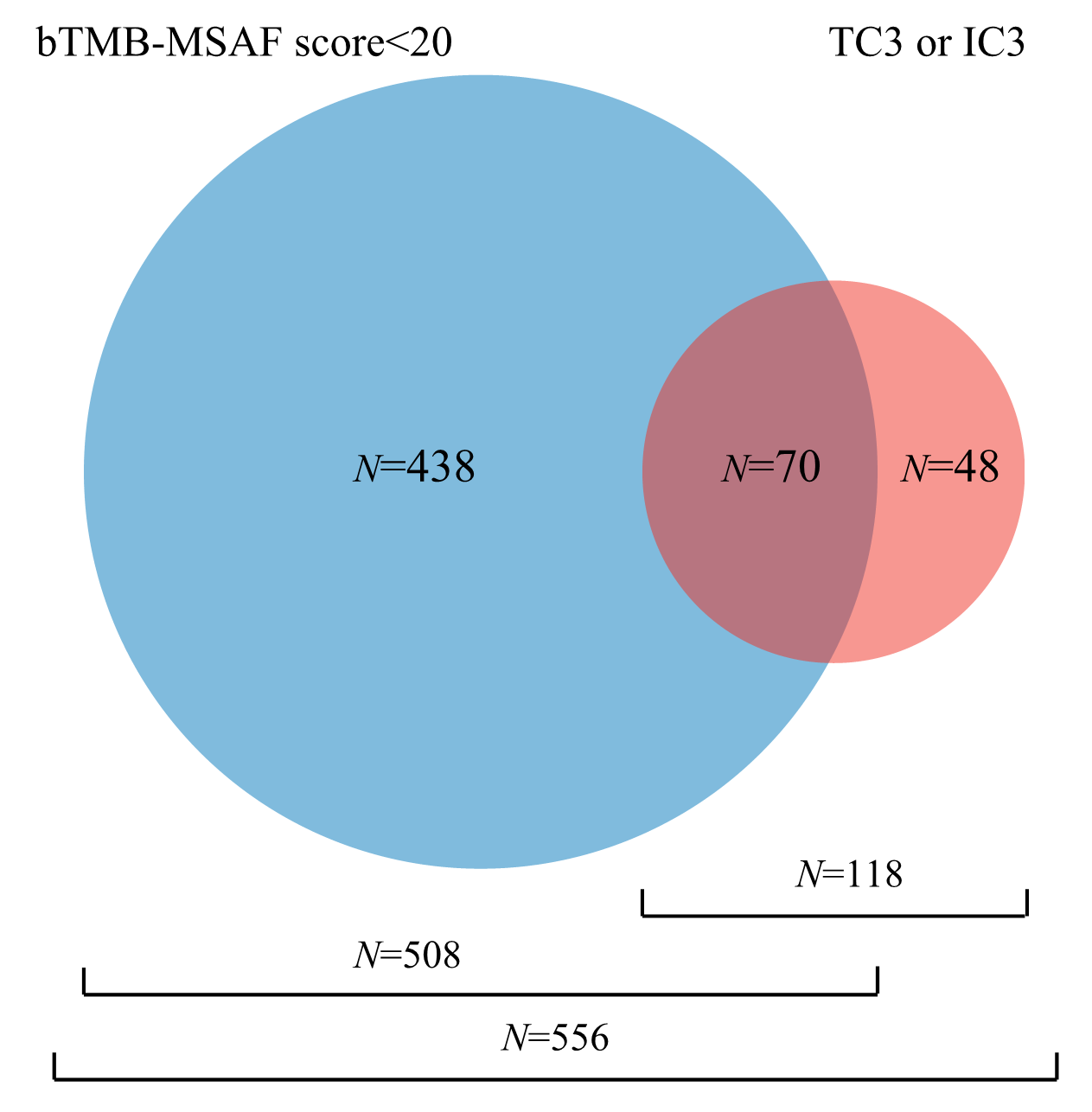


TC3 or IC3 indicates that over 50% of tumor cells or over 10% of tumor infiltrating immune cells expressed PD-L1.

**Figure S17.** The distribution of bTMB-MSAF algorithm in the EGFR wild-type patients in different PD-L1 expression subgroups.


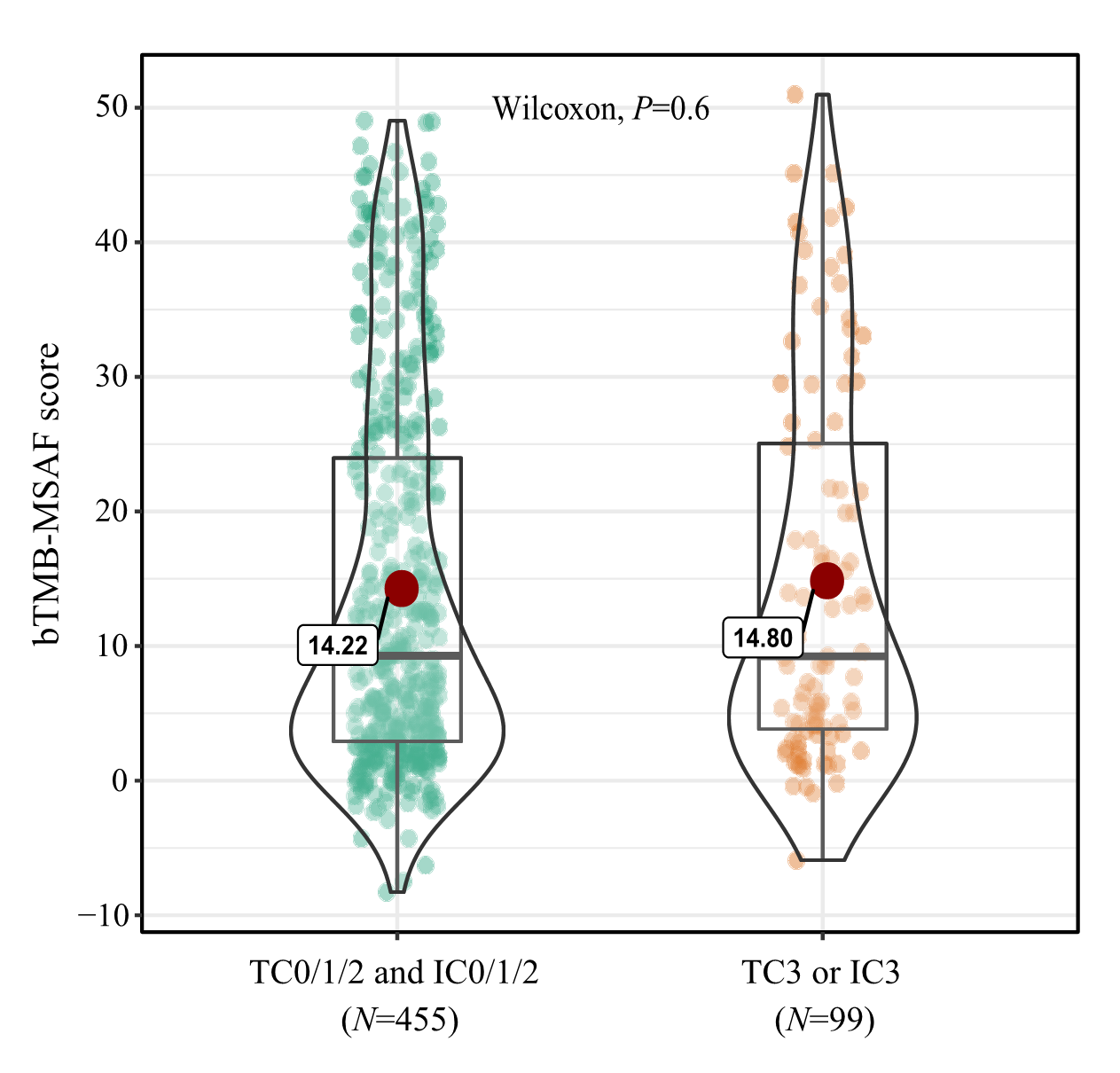


PD-L1, programmed cell death ligand 1. TC0/1/2 and IC0/1/2 indicates that less than 50% of tumor cells and less than 10% of tumor infiltrating immune cells expressed PD-L1, and TC3 or IC3 indicates that over 50% of tumor cells or over 10% of tumor infiltrating immune cells expressed PD-L1.

**Figure S18.** Survival analysis stratified by treatment in EGFR wild-type patients with a bTMB-MSAF algorithm≥20.


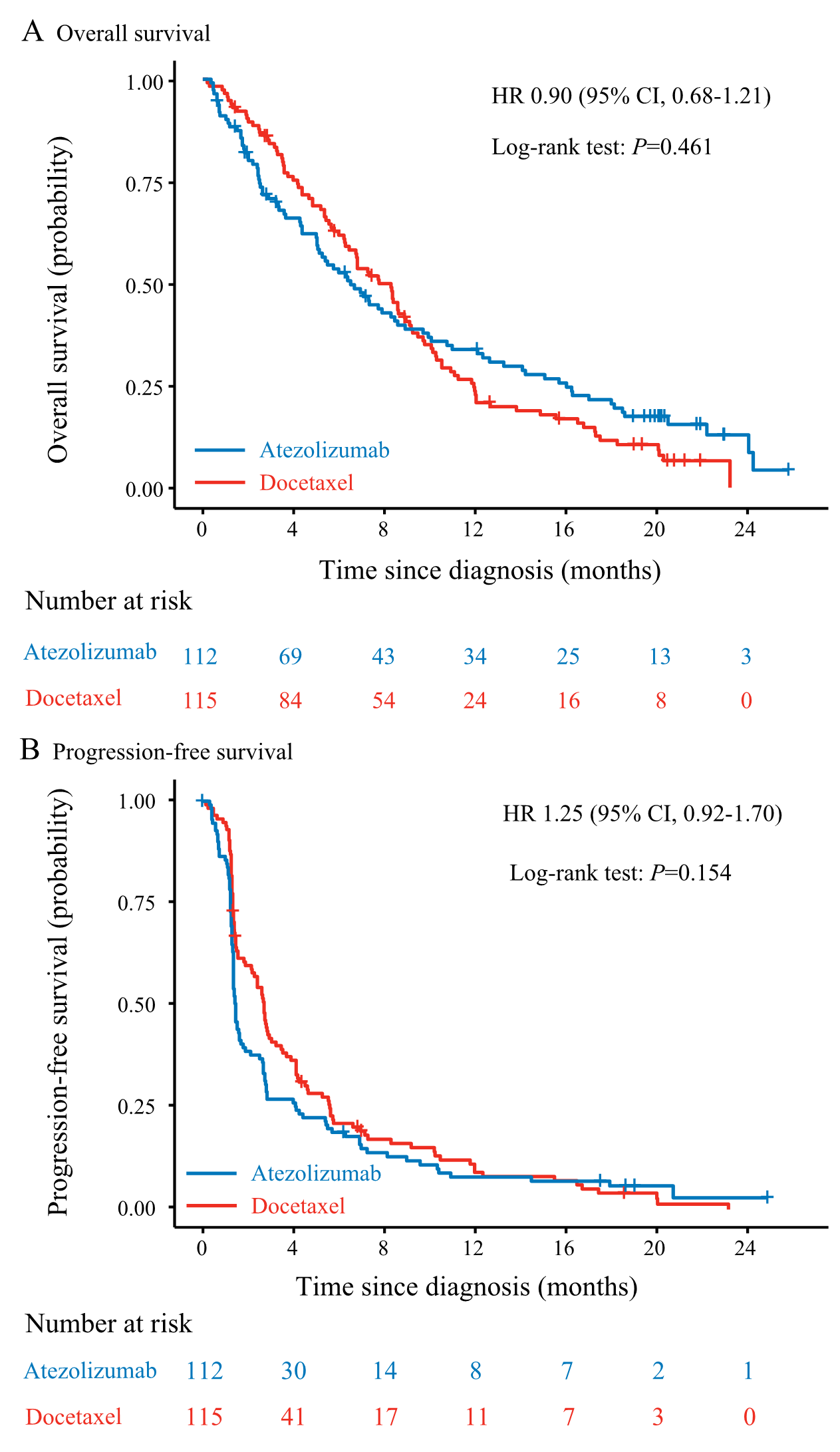


(A) Overall survival. (B) Progression-free survival. HR, hazard ratio; CI, confidence interval.

**Figure S19.** Survival analysis based on a range of cut-points of the bTMB-MSAF algorithm in EGFR mutant patients.


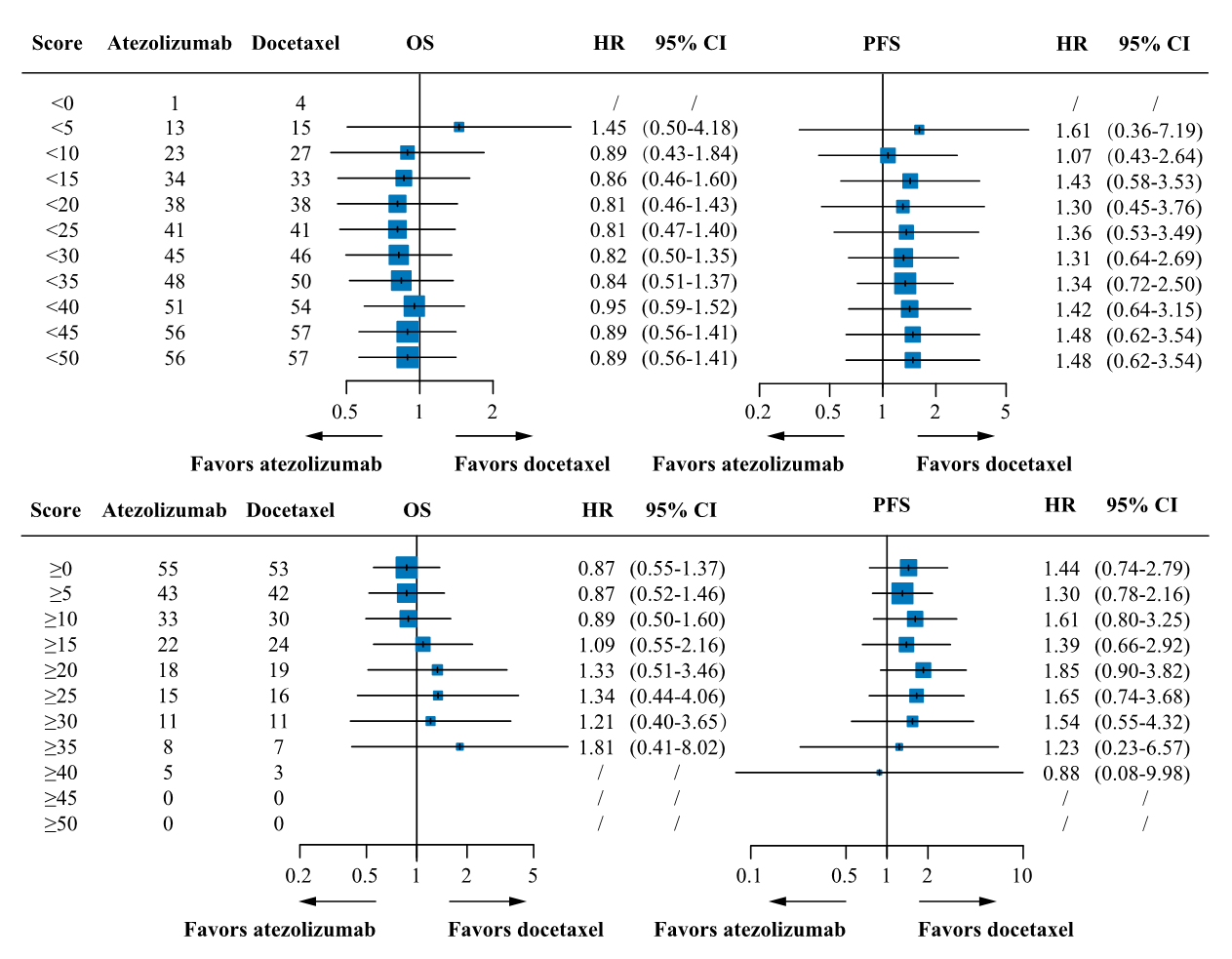


OS, overall survival; PFS, progression-free survival; HR, hazard ratio; CI, confidence interval.

**Figure S20.** Characterization of blood-based genomic alterations in advanced non-small-cell lung cancer.


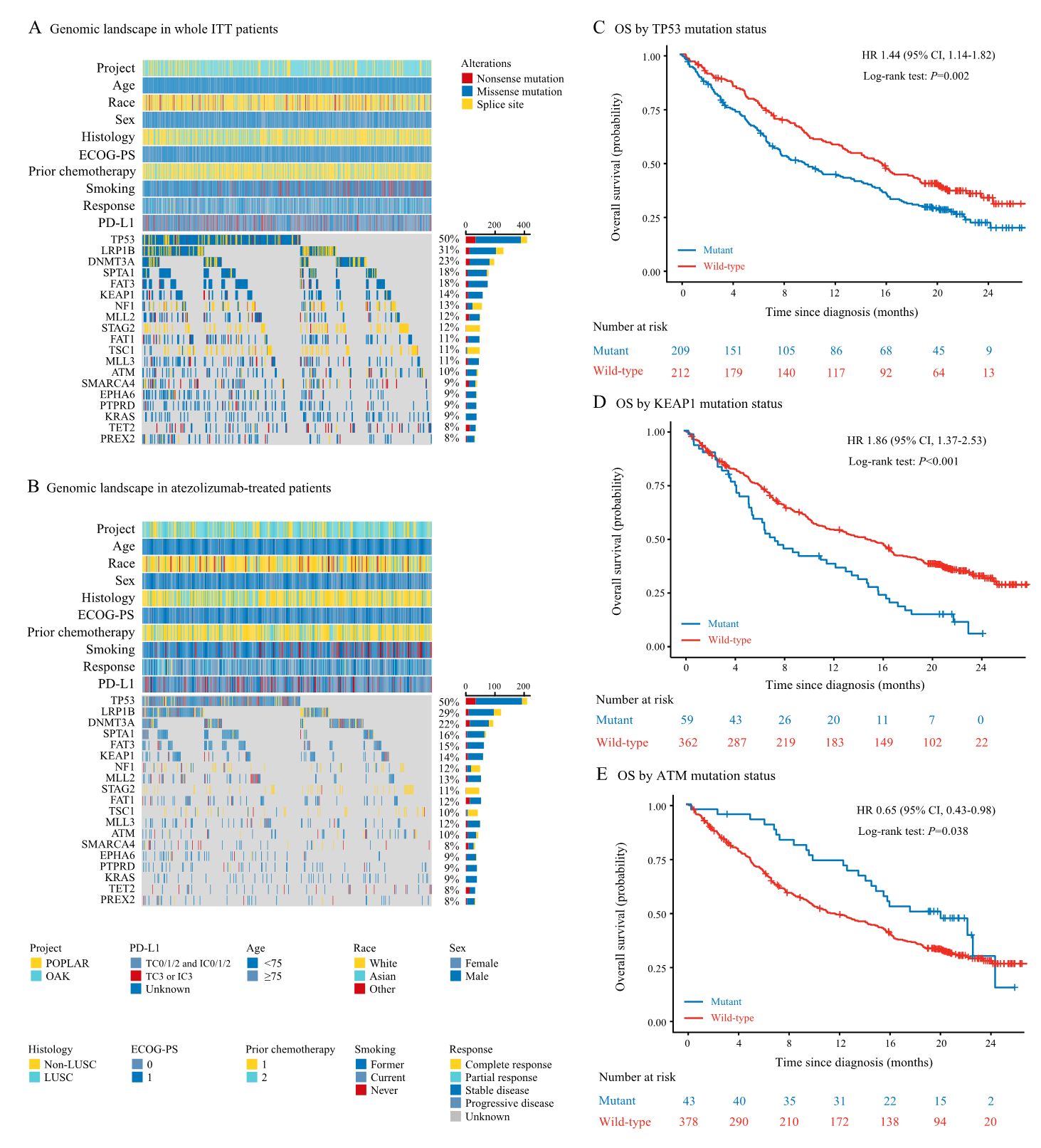


(A) and (B) Oncoprints depicting the landscape of blood-based genomic alterations in the whole intention-to-treat patients and atezolizumab-treated patients, respectively. (C)–(F) Overall survival analyses stratified by mutation status of TP53, KEAP1, and ATM, respectively. ECOG-PS, Eastern Cooperative Oncology Group Performance Status; PD-L1, programmed cell death ligand 1; LUSC, lung squamous cell carcinoma; HR, hazard ratio; CI, confidence interval; OS, overall survival. TC0/1/2 and IC0/1/2 indicates that less than 50% of tumor cells and less than 10% of tumor-infiltrating immune cells expressed PD-L1, and TC3 or IC3 indicates that over 50% of tumor cells or over 10% of tumor-infiltrating immune cells expressed PD-L1.

**Figure S21.** Landscape of blood-based genomic alterations in atezolizumab-treated EGFR wild-type or mutant patients.


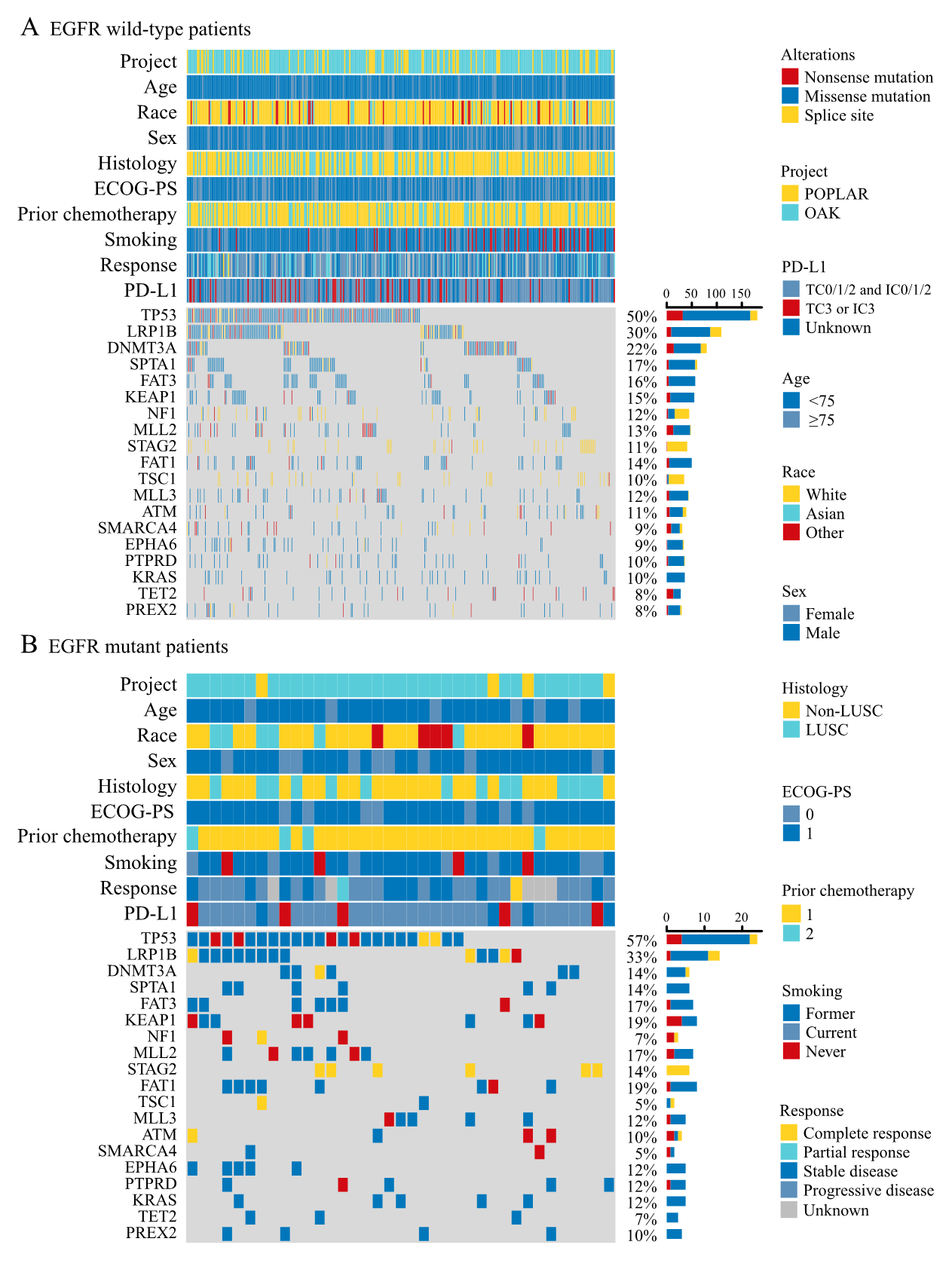


(A) EGFR wild-type. (B) EGFR mutant patients. ECOG-PS, Eastern Cooperative Oncology Group Performance Status; PD-L1, programmed cell death ligand 1; LUSC, lung squamous cell carcinoma. TC0/1/2 and IC0/1/2 indicates that less than 50% of tumor cells and less than 10% of tumor infiltrating immune cells expressed PD-L1, and TC3 or IC3 indicates that over 50% of tumor cells or over 10% of tumor infiltrating immune cells expressed PD-L1.

**Figure S22.** Correlation of genomic mutations with blood-based tumor mutation burden in EGFR wild-type patients.


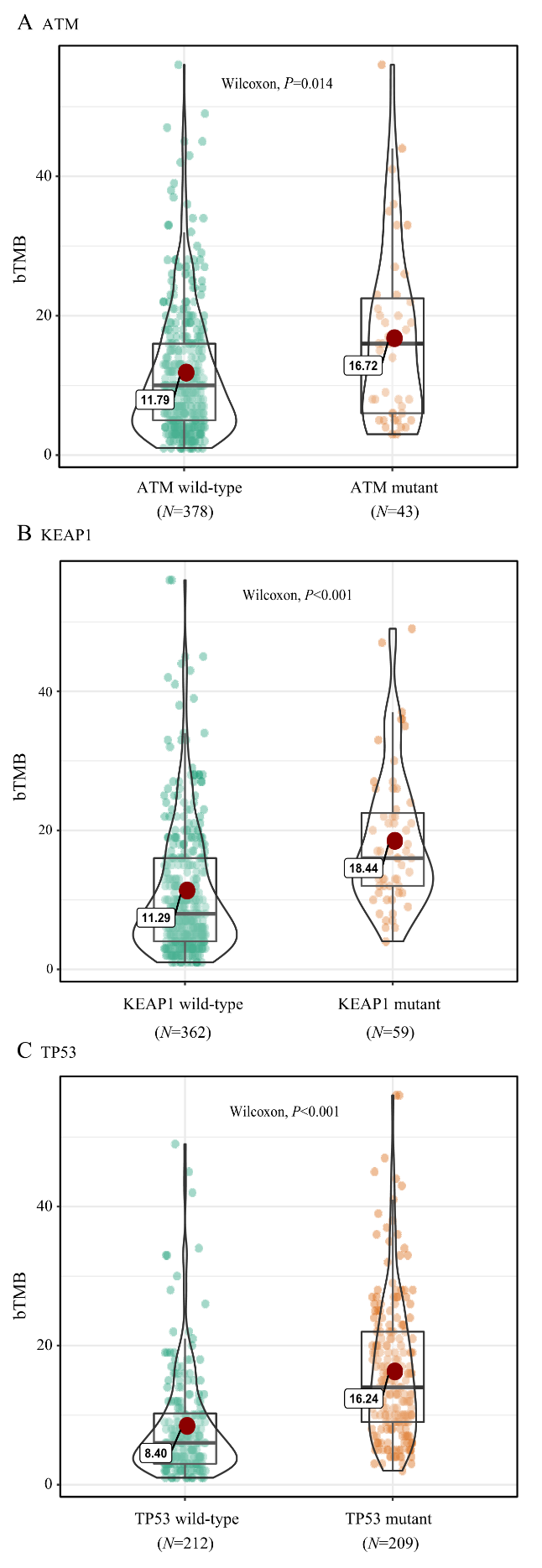


(A) ATM. (B) KEAP1. (C) TP53. bTMB indicates blood-based tumor mutation burden

**Figure S23.** Nomogram A to predict the survival of patients undergoing atezolizumab.

**
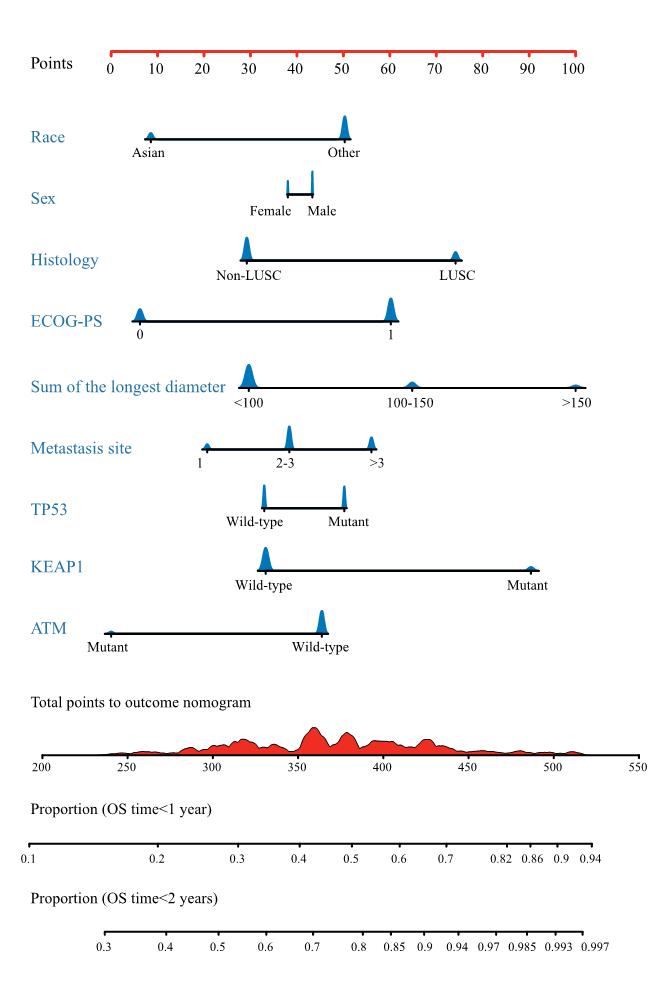
**

ECOG-PS, Eastern Cooperative Oncology Group Performance Status; LUSC, lung squamous cell carcinoma; OS, overall survival.

**Figure S24.** Identification of the optimal cut-points for risk stratification in the nomograms.


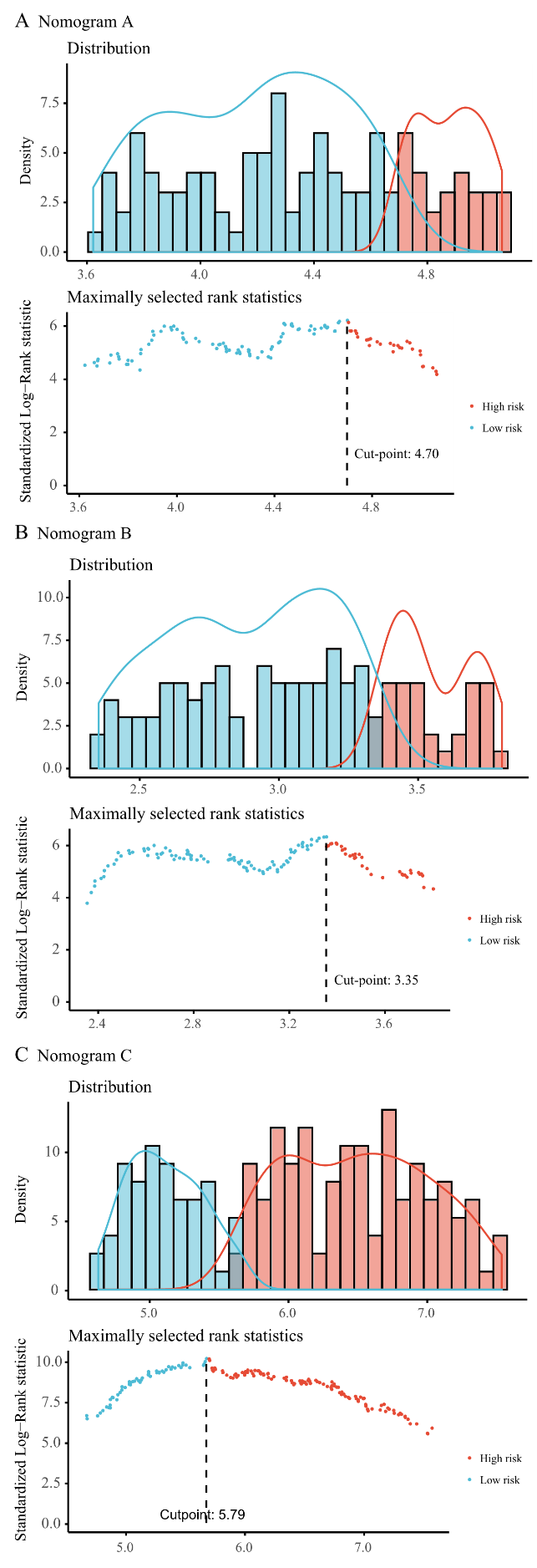


(A) Nomogram A. (B) Nomogram B. (C) Nomogram C.

**Figure S25.** Overall survival analysis based on risk stratification using nomogram A.


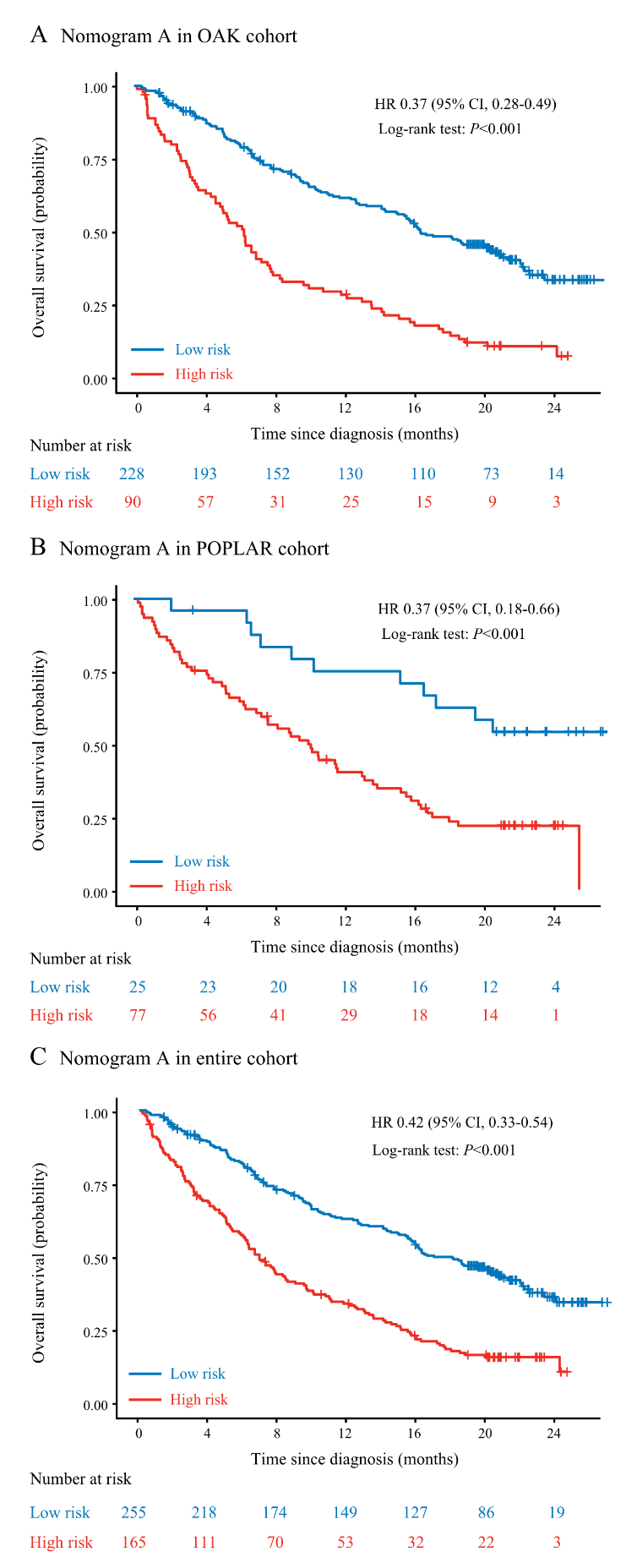


(A) OAK cohort. (B) POPLAR cohort. (C) The combined population of the POPLAR and OAK cohorts. HR, hazard ratio; CI, confidence interval.

**Figure S26.** Calibration curves for the nomogram A model to predict overall survival of atezolizumab-treated patients.


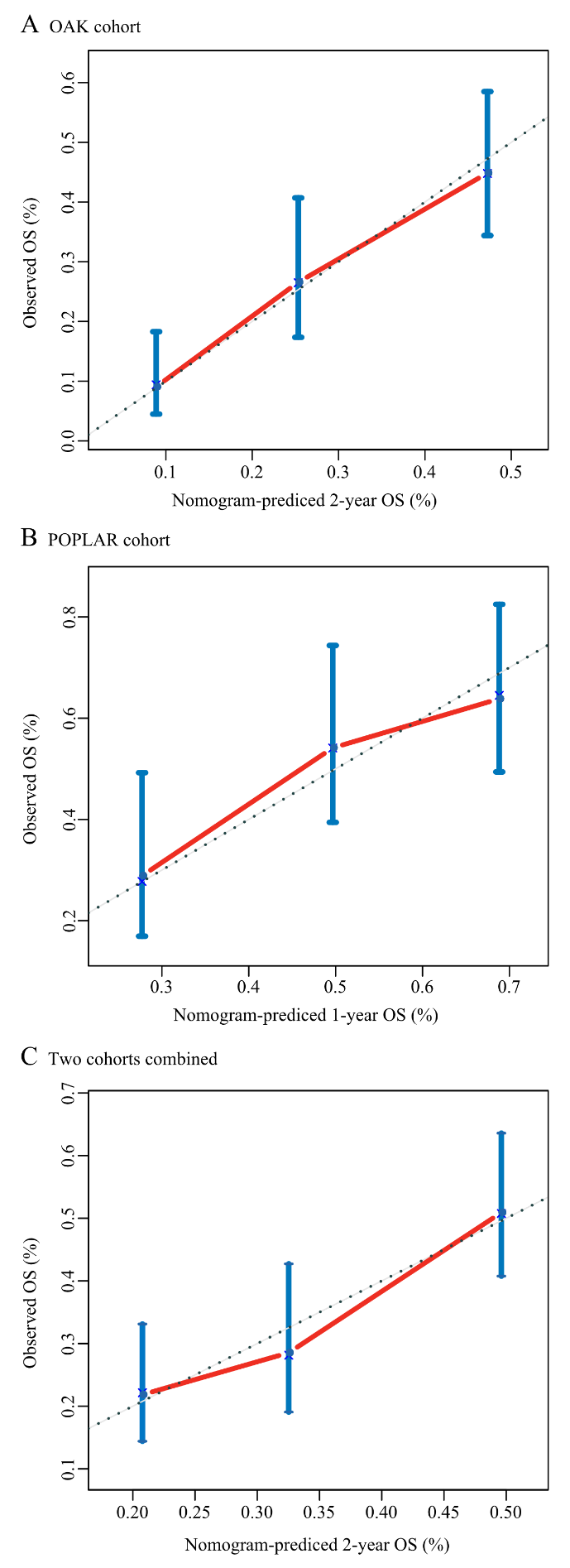


(A) OAK cohort. (B) POPLAR cohort. (C) The combined population of the POPLAR and OAK cohorts. OS, overall survival.

**Figure S27.** Decision curve analysis of nomogram A.


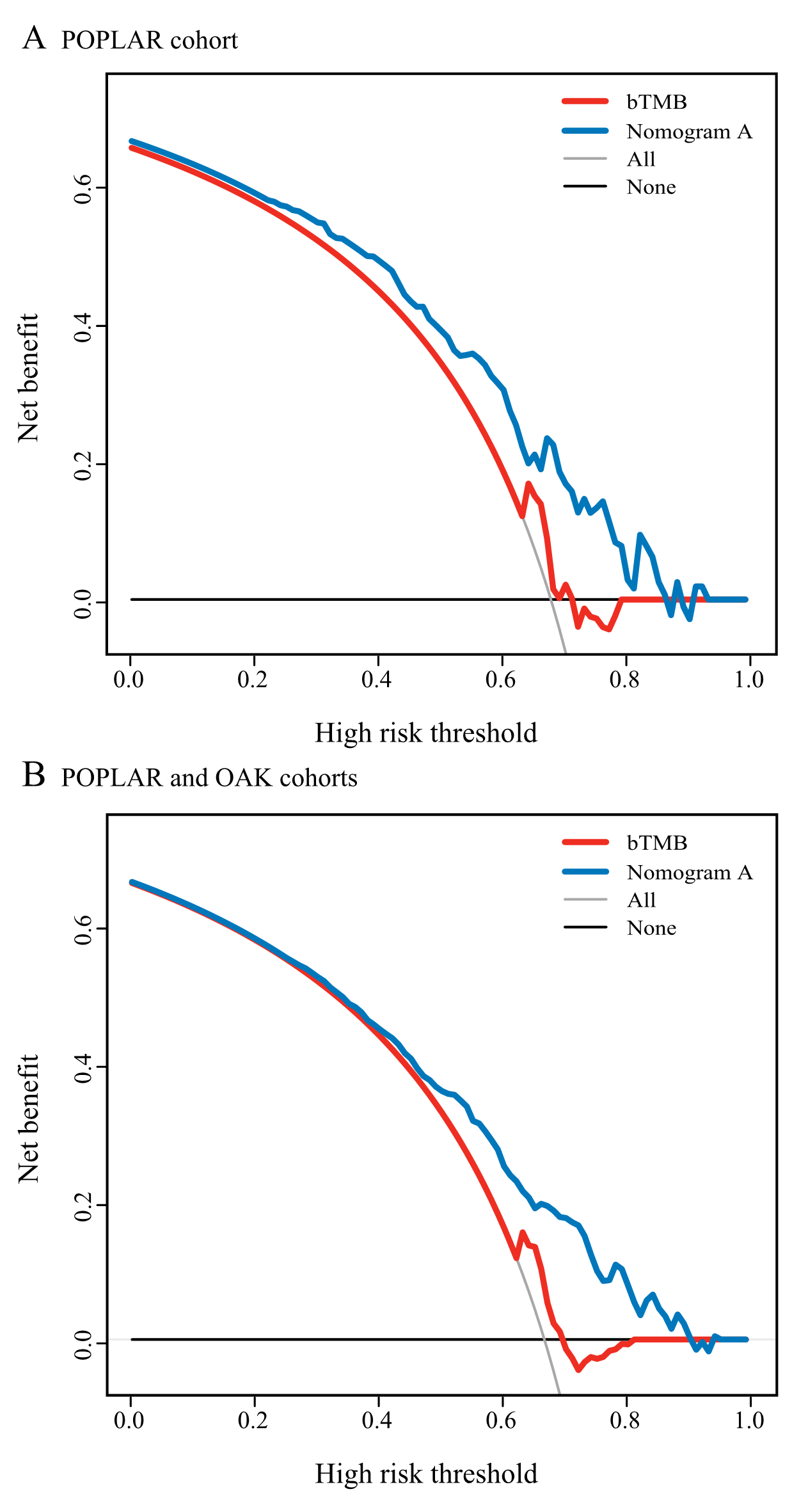


(A) POPLAR cohort. (B) The combined population of the POPLAR and OAK cohorts. bTMB indicated blood-based tumor mutation burden

**Figure S28.** Nomogram B to predict the survival of patients undergoing atezolizumab.


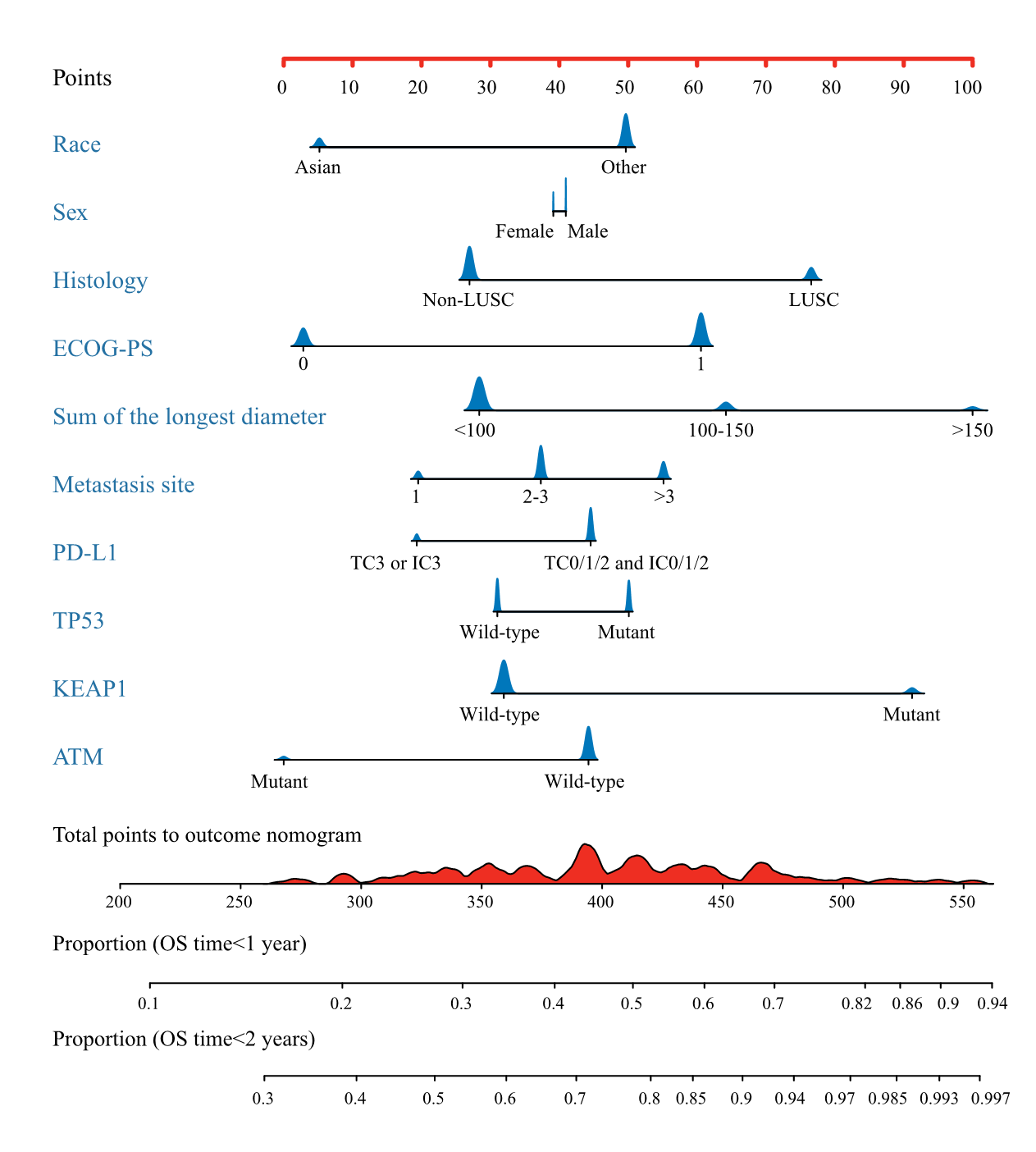


ECOG-PS, Eastern Cooperative Oncology Group Performance Status; LUSC, lung squamous cell carcinoma; PD-L1, programmed cell death ligand 1; OS, overall survival; TC0/1/2 and IC0/1/2 indicates that less than 50% of tumor cells and less than 10% of tumor-infiltrating immune cells expressed PD-L1, and TC3 or IC3 indicates that over 50% of tumor cells or over 10% of tumor-infiltrating immune cells expressed PD-L1.

**Figure S29.** Overall survival analysis based on risk stratification using the nomogram B.


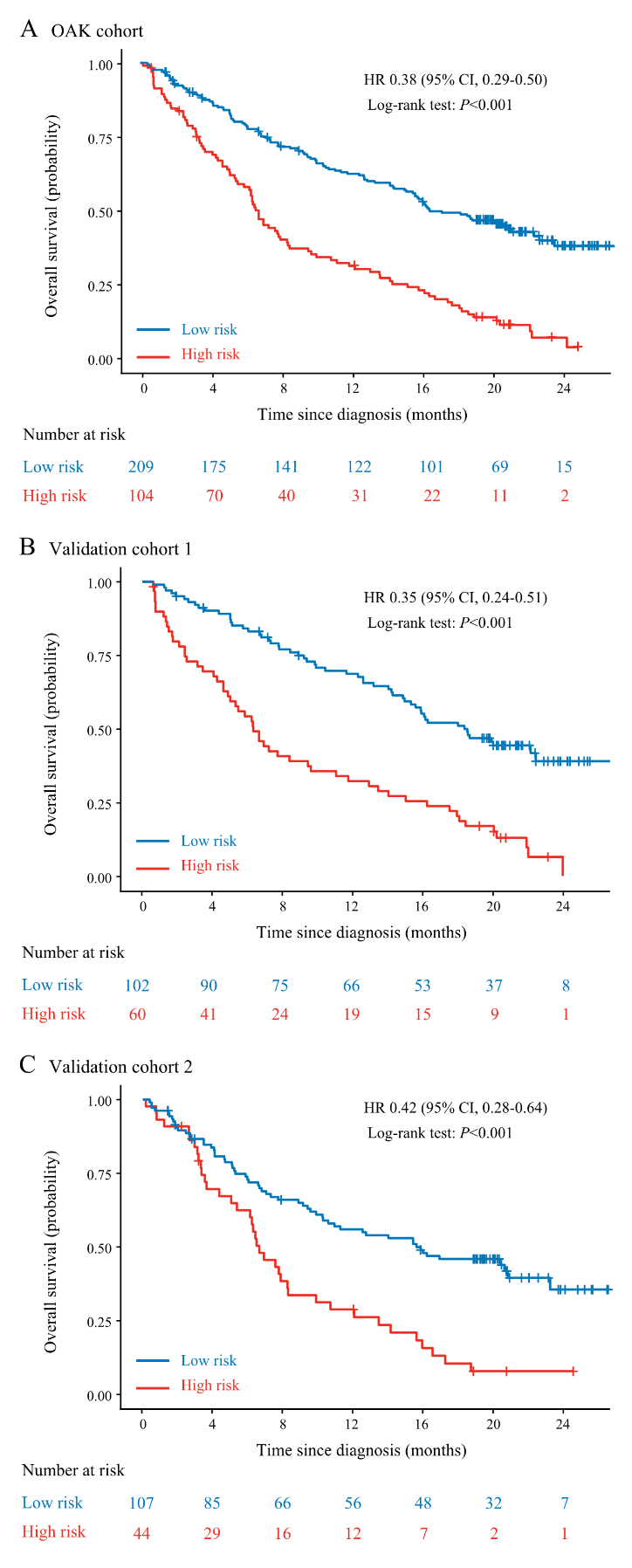


(A) OAK cohort. (B) Validation cohort 1. (C) Validation cohort 2. HR, hazard ratio; CI, confidence interval.

**Figure S30.** Calibration curves for the nomogram B to predict overall survival of atezolizumab-treated patients.

**
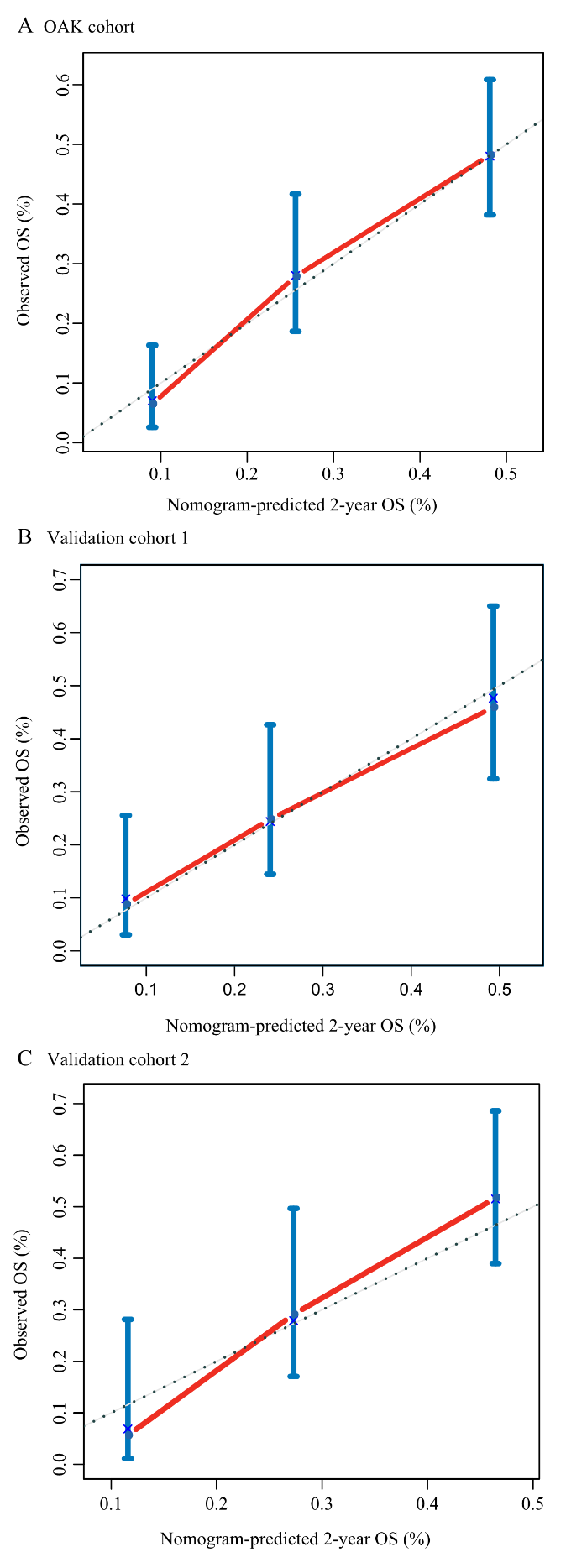
**

(A) OAK cohort. (B) Validation cohort 1. (C) Validation cohort 2. OS, overall survival.

**Figure S31.** Decision curve analysis for the internal validation of the nomograms B and C.


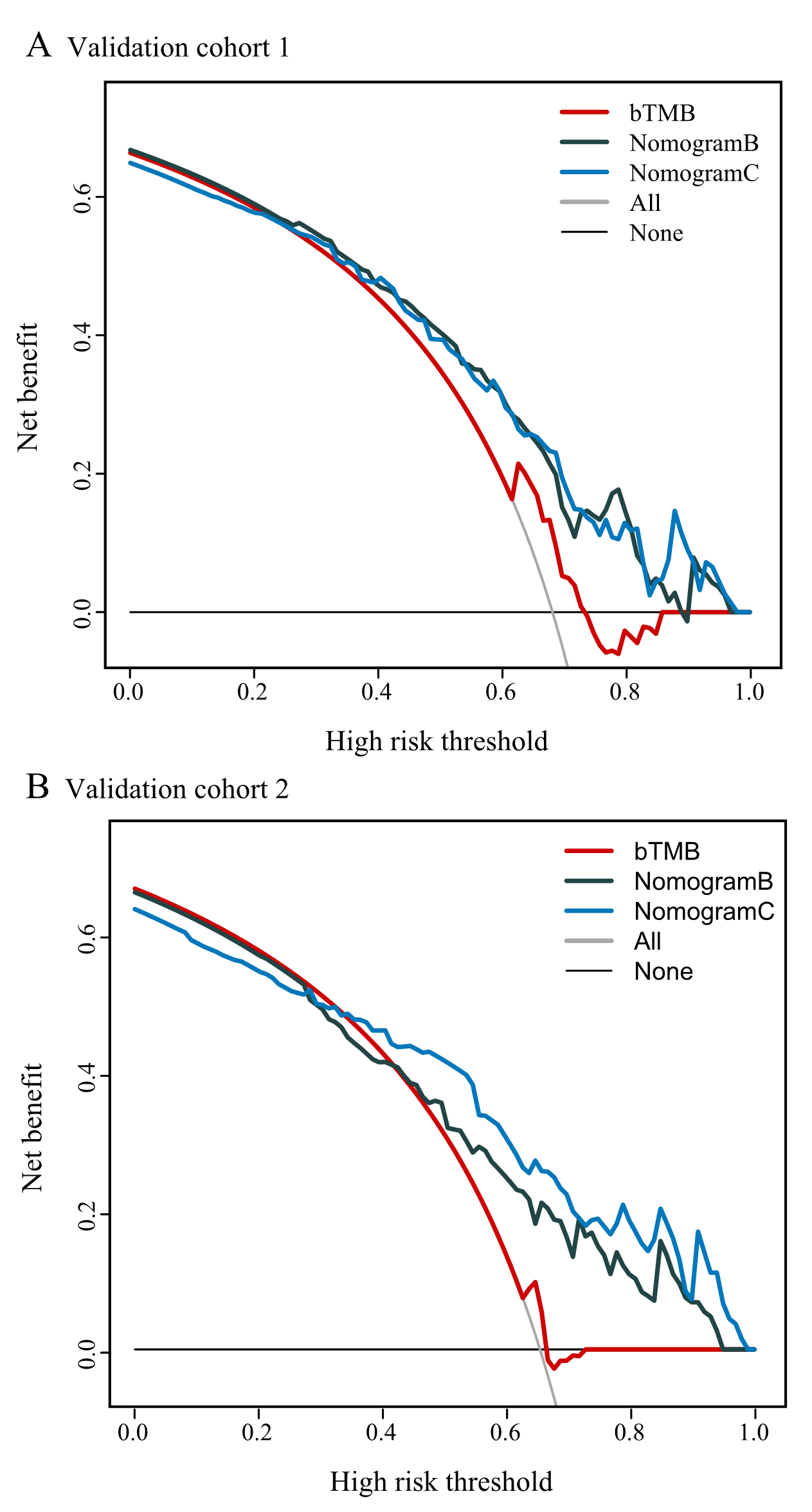


(A) Validation cohort 1. (B) Validation cohort 2. bTMB, blood-based tumor mutation burden.

**Figure S32.** Overall survival analysis stratified by treatment response in the combined population of the POPLAR and OAK cohorts.


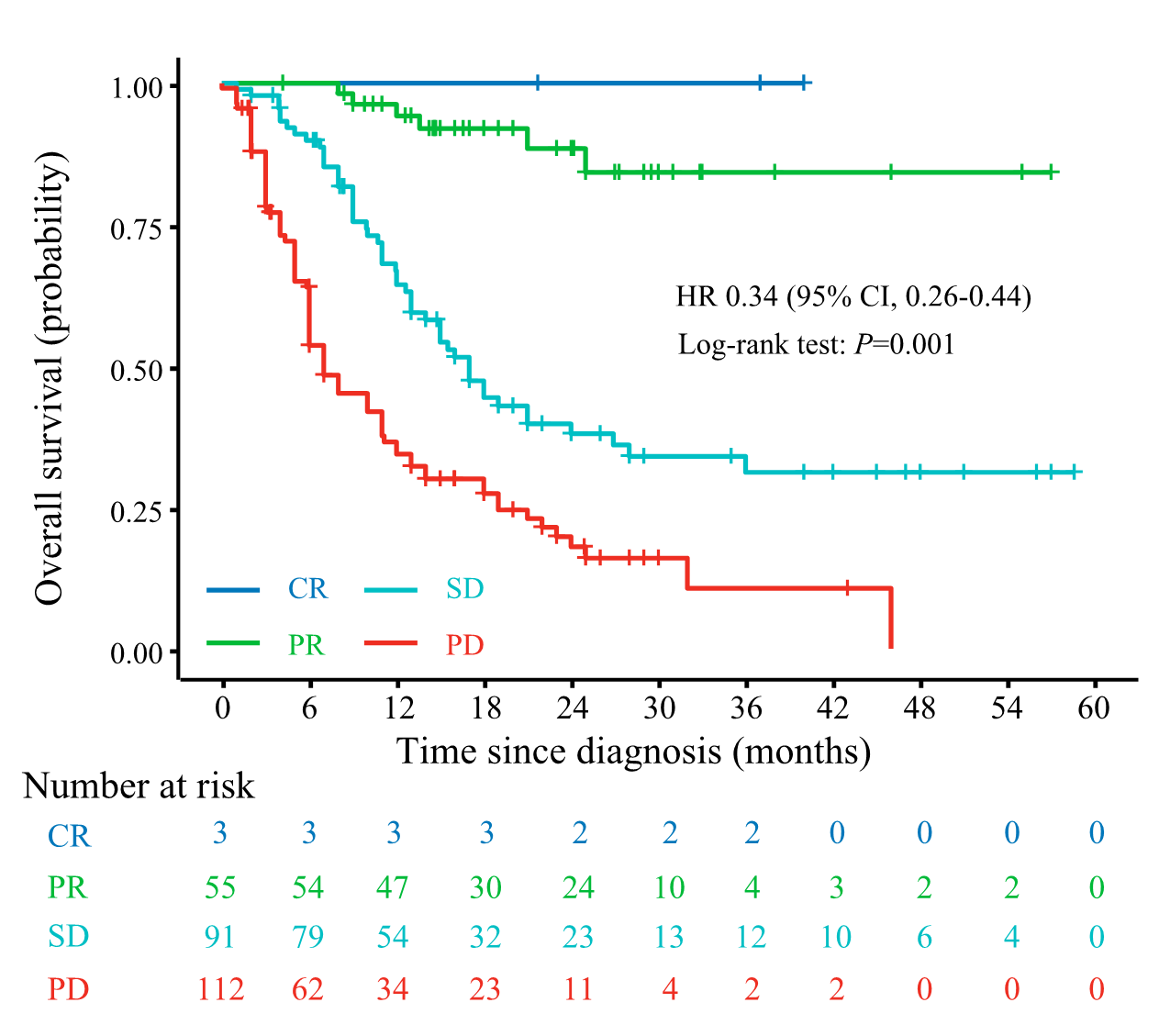


CR, complete response; PR, progressive response; SD, stable disease; PD, progressive disease; HR, hazard ratio; CI, confidence interval.

**Figure S33.** Nomogram C to predict the survival of patients undergoing atezolizumab.


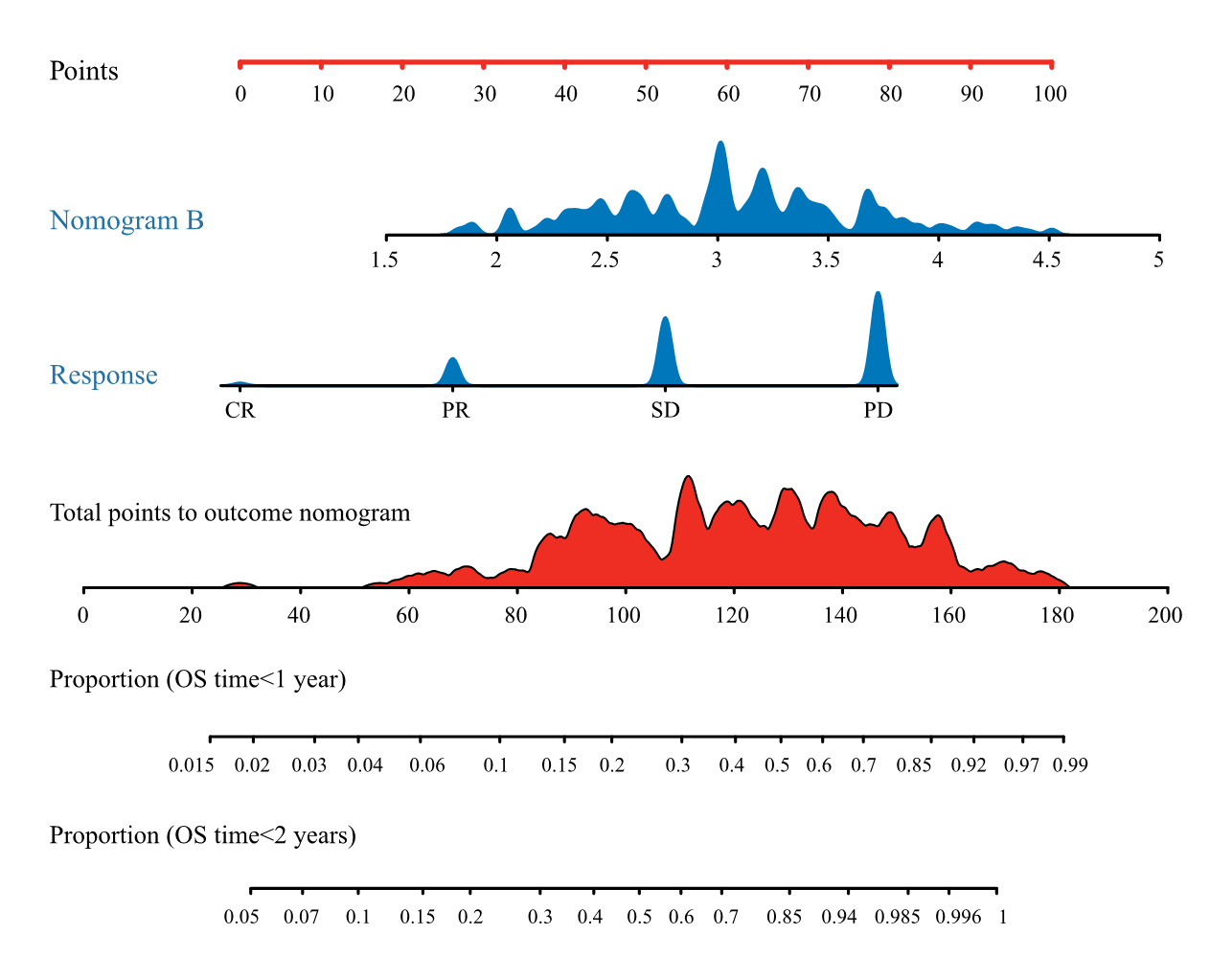


CR, complete response; PR, progressive response; SD, stable disease; PD, progressive disease; OS, overall survival.

**Figure S34.** Overall survival analysis based on risk stratification using the nomogram C.


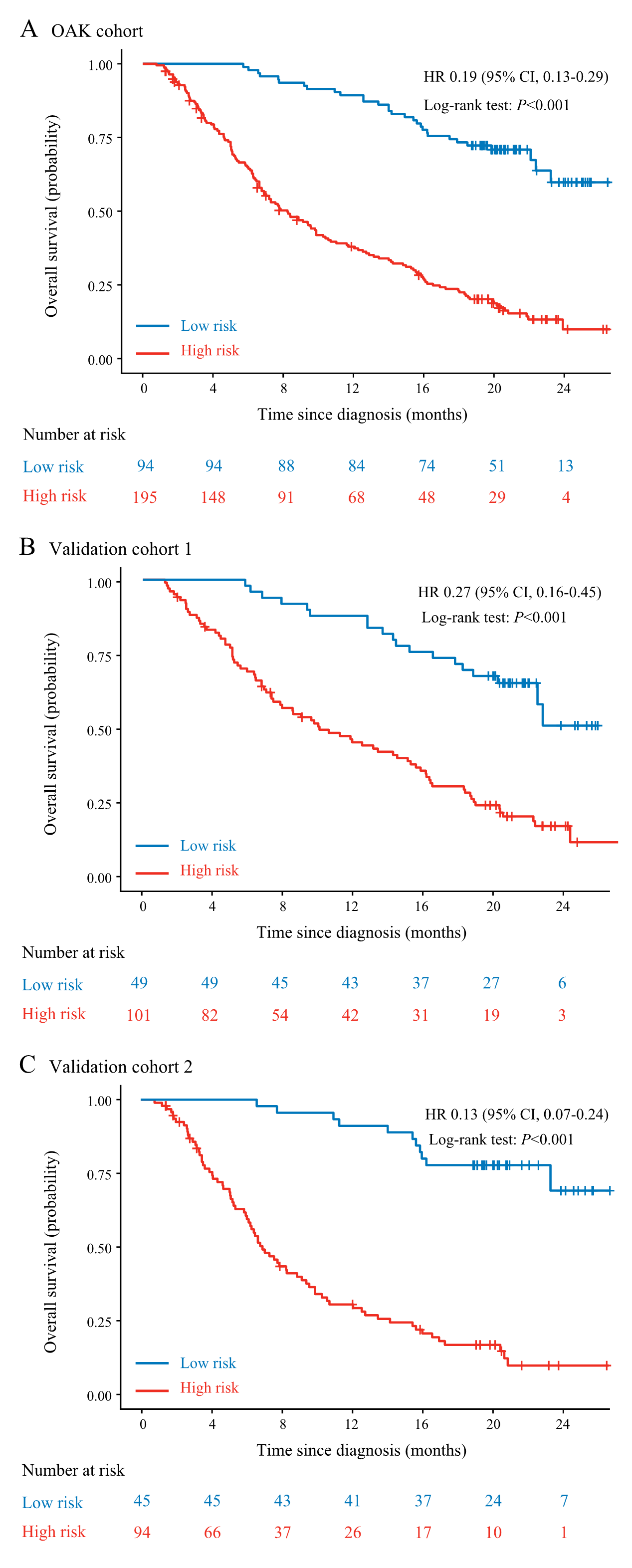


(A) OAK cohort. (B) Validation cohort 1. (C) Validation cohort 2. HR, hazard ratio; CI, confidence interval.

**Figure S35.** Overall survival analysis based on risk stratification using nomogram C in EGFR wild-type patients with a bTMB-MSAF algorithm <20 or ≥20.


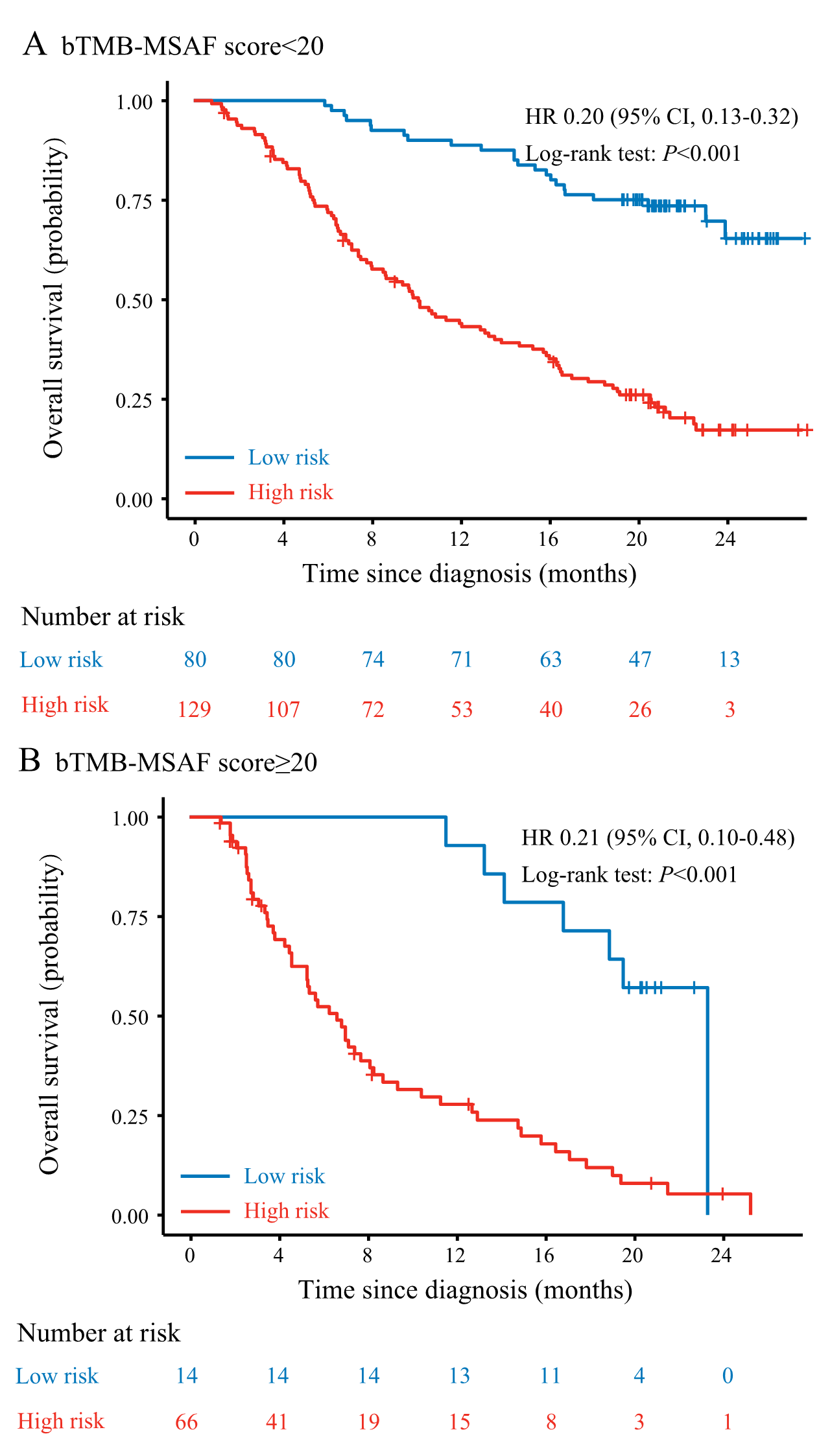


(A) and (B) Patients with a bTMB-MSAF algorithm<20 and ≥20, respectively. HR, hazard ratio; CI, confidence interval.

**Figure S36.** Calibration curves for the nomogram C to predict overall survival of atezolizumab-treated patients.


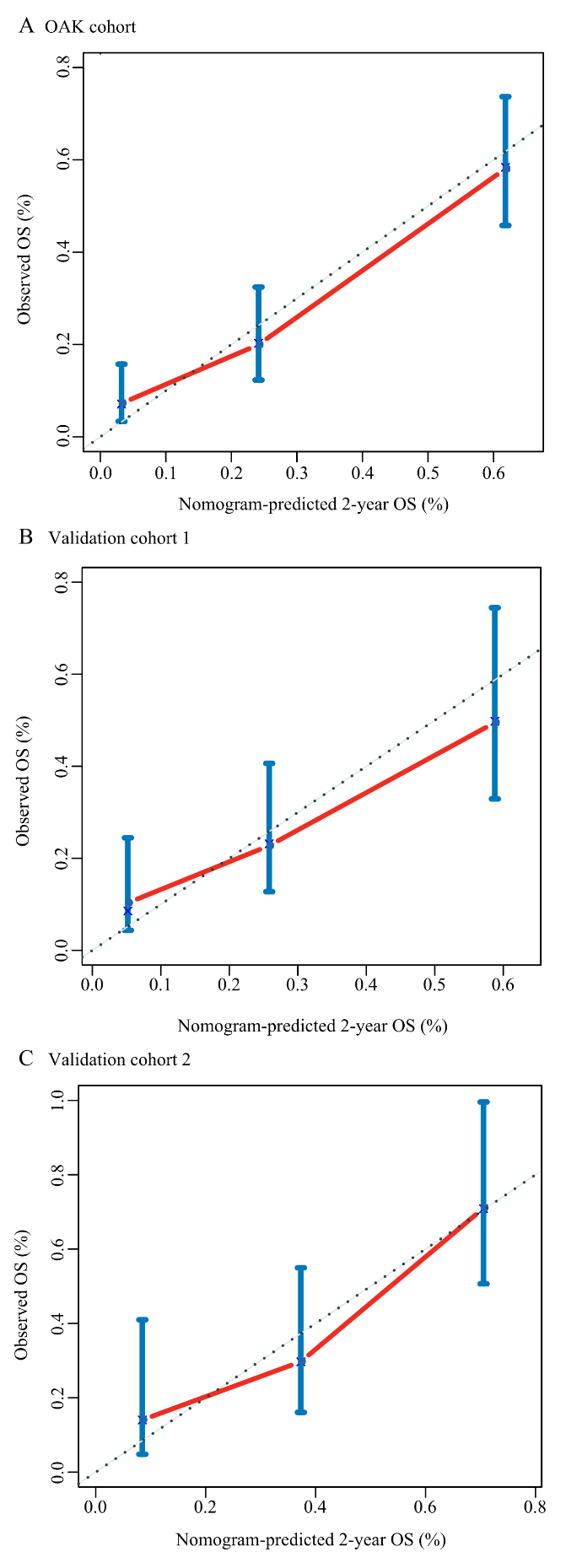


(A) OAK cohort. (B) Validation cohort 1. (C) Validation cohort 2. OS, overall survival.

**Table S1.** Clinical characteristics of EGFR wild-type patients in the OAK and POPLAR trials.

| **Characteristic** | | ***N* (%) ^a^** | | | | |  |  | ***N* (%) ^b^** | |  |
| --- | --- | --- | --- | --- | --- | --- | --- | --- | --- | --- | --- |
|  |  | **Atezolizumab** | | **Docetaxel** | ***P* value** | | |  | **Atezolizumab** | **Docetaxel** | ***P* value** |
| **No. (%)** | 357 (50.1) | | 356 (49.9) | | |  | |  | 109 (50.2) | 108 (49.8) |  |
| **Sex, *N* (%)** |  | |  | | | 0.854 | |  |  |  | 0.011 |
| Female | 129 (36.1) | | 132 (37.1) | | |  | |  | 33 (30.3) | 52 (48.1) |  |
| Male | 228 (63.9) | | 224 (62.9) | | |  | |  | 76 (69.7) | 56 (51.9) |  |
| **Age (years)** |  | |  | | |  | |  |  |  |  |
| Mean (95% CI) | 63.6 (62.6–64.6) | | 63.9 (62.9–64.9) | | | 0.698 | |  | 61.7 (60.0–63.5) | 62.5 (60.7–64.2) | 0.561 |
| <75 | 307 (86.0) | | 322 (90.4) | | | 0.084 | |  | 99 (90.8) | 98 (90.7) | 1.000 |
| ≥75 | 50 (14.0) | | 34 (9.6) | | |  | |  | 10 (9.2) | 10 (9.3) |  |
| **Mean follow-up**  **(months, 95% CI)** | 12.8 (12.0–13.6) | | 9.9 (9.1–10.7) | | |  | |  | 12.4 (10.9–14.0) | 9.8 (8.4–11.1) |  |
| **Race, *N* (%)** |  | |  | | | 0.921 | |  |  |  | 0.981 |
| White | 260 (72.8) | | 264 (74.2) | | |  | |  | 88 (80.7) | 88 (81.5) |  |
| Asian | 64 (17.9) | | 61 (17.1) | | |  | |  | 12 (11.0) | 11 (10.2) |  |
| Other | 33 (9.2) | | 31 (8.7) | | |  | |  | 9 (8.3) | 9 (8.3) |  |
| **ECOG-PS, *N* (%)** |  | |  | | | 0.854 | |  |  |  | 0.961 |
| 0 | 126 (35.3) | | 129 (36.2) | | |  | |  | 37 (33.9) | 38 (35.2) |  |
| 1 | 231 (64.7) | | 227 (63.8) | | |  | |  | 72 (66.1) | 70 (64.8) |  |
| **Histologic type, *N* (%)** |  | |  | | | 0.884 | |  |  |  | 1.000 |
| Non-LUSC | 259 (72.5) | | 261 (73.3) | | |  | |  | 71 (65.1) | 70 (64.8) |  |
| LUSC | 98 (27.5) | | 95 (26.7) | | |  | |  | 38 (34.9) | 38 (35.2) |  |
| **Smoking status, *N* (%)** |  | |  | | | 0.160 | |  |  |  | 0.340 |
| Current | 53 (14.8) | | 62 (17.4) | | |  | |  | 22 (20.2) | 19 (17.6) |  |
| Former | 245 (68.6) | | 252 (70.8) | | |  | |  | 72 (66.1) | 66 (61.1) |  |
| Never | 59 (16.5) | | 42 (11.8) | | |  | |  | 15 (13.8) | 23 (21.3) |  |
| **Prior courses of chemotherapy, *N* (%)** |  | |  | | | 0.492 | |  |  |  | 0.332 |
| 1 | 269 (75.4) | | 277 (77.8) | | |  | |  | 70 (64.2) | 77 (71.3) |  |
| 2 | 88 (24.6) | | 79 (22.2) | | |  | |  | 39 (35.8) | 31 (28.7) |  |
| **PD-L1 expression, *N* (%)** |  | |  | | | 0.680 | |  |  |  | NR |
| TC0 and IC0 | 151 (42.7) | | 157 (44.5) | | |  | |  | NR | NR |  |
| TC1/2/3 or IC1/2/3 | 203 (57.3) | | 196 (55.5) | | |  | |  | NR | NR |  |
| **PD-L1 expression, *N* (%)** |  | |  | | | 0.749 | |  |  |  | NR |
| TC0/1/2 and IC0/1/2 | 292 (82.9) | | 297 (83.9) | | |  | |  | NR | NR |  |
| TC3 or IC3 | 61 (17.1) | | 57 (16.1) | | |  | |  | NR | NR |  |
| **KRAS, *N* (%)** |  | |  | | | 0.510 | |  |  |  | 0.393 |
| Positive | 46 (15.2) | | 54 (17.5) | | |  | |  | 21 (21.2) | 15 (15.5) |  |
| Negative | 257 (84.8) | | 255 (82.5) | | |  | |  | 78 (78.8) | 82 (84.5) |  |
| **bTMB** |  | |  | | |  | |  |  |  |  |
| (mean, 95% CI) | 9.4 (8.4–10.4) | | 9.6 (8.6–10.7) | | | 0.590 | |  | 11.0 (9.0–13.0) | 11.6 (9.2–14.1) | 0.642 |
| <18 | 212 (75.4) | | 202 (73.2) | | | 0.608 | |  | 67 (74.4) | 59 (66.3) | 0.303 |
| ≥18 | 69 (24.6) | | 74 (26.8) | | |  | |  | 23 (25.6) | 30 (33.7) |  |
| **MSAF**  **(mean, 95% CI)** | 0.06 (0.05–0.07) | | 0.06 (0.05–0.07) | | | 1.000 | |  | 0.07 (0.06–0.08) | 0.06 (0.05–0.07) | 0.442 |
| **SLD, mm** |  | |  | | |  | |  |  |  |  |
| (mean, 95% CI) | 76.4 (71.3–81.5) | | 77.4 (72.4–82.4) | | | 0.840 | |  | 81.3 (71.7–90.8) | 84.4 (75.1–93.7) | 0.645 |
| <81 | 226 (63.2) | | 215 (60.4) | | | 0.488 | |  | 65 (59.6) | 61 (56.5) | 0.739 |
| ≥81 | 131 (36.8) | | 141 (39.6) | | |  | |  | 44 (40.4) | 47 (43.5) |  |
| **Metastasis sites** |  | |  | | | 0.673 | |  |  |  | 0.285 |
| 1 | 51 (14.3) | | 46 (12.9) | | |  | |  | 10 (9.2) | 16 (14.8) |  |
| >1 | 306 (85.7) | | 310 (87.1) | | |  | |  | 99 (90.8) | 92 (85.2) |  |
| **Best response, *N* (%)** |  | |  | | | <0.001 | |  |  |  | 0.805 |
| CR | 5 (1.5) | | 1 (0.3) | | |  | |  | 0 (0.0) | 0 (0.0) |  |
| PR | 47 (14.2) | | 41 (14.0) | | |  | |  | 19 (19.2) | 14 (15.6) |  |
| SD | 120 (36.1) | | 153 (52.4) | | |  | |  | 39 (39.4) | 37 (41.1) |  |
| PD | 160 (48.2) | | 97 (33.2) | | |  | |  | 41 (41.4) | 39(43.3) |  |

^a^Patients in OAK cohort.

^b^Patients in POPLAR cohort.

ECOG-PS, Eastern Cooperative Oncology Group Performance Status; LUSC, lung squamous cell carcinoma; SLD, sum of the longest diameter; PD-L1, programmed cell death ligand 1; CR, complete response; PR, partial response; SD, stable disease; PD, progressive disease; CI, confidence interval; NR, not reported; bTMB indicates blood-based tumor mutation burden; MSAF indicates ctDNA maximum somatic allele frequency. TC0 and IC0 indicates that less than 1% of tumor cells and less than 1% of tumor infiltrating immune cells expressed PD-L1; TC0/1/2 and IC0/1/2 indicates that less than 50% of tumor cells and less than 10% of tumor infiltrating immune cells expressed PD-L1; TC1/2/3 or IC1/2/3 indicates that over 1% of tumor cells or over 1% of tumor infiltrating immune cells expressed PD-L1; TC3 or IC3 indicates that over 50% of tumor cells or over 10% of tumor infiltrating immune cells expressed PD-L1.

**Table S2.** Clinical characteristics of EGFR mutant patients in the OAK and POPLAR trials.

| **Characteristic** | ***N* (%) ^a^** |  |  |  | ***N* (%) ^b^** |  |  |
| --- | --- | --- | --- | --- | --- | --- | --- |
|  | **Atezolizumab** | **Docetaxel** | ***P* value** |  | **Atezolizumab** | **Docetaxel** | ***P* value** |
| **No. (%)** | 54 (48.6) | 57 (51.4) |  |  | 19 (51.4) | 18 (48.6) |  |
| **Sex, *N* (%)** |  |  | 0.888 |  |  |  | 0.243 |
| Female | 32 (59.3) | 32 (56.1) |  |  | 11 (57.9) | 6 (33.3) |  |
| Male | 22 (40.7) | 25 (43.9) |  |  | 8 (42.1) | 12 (66.7) |  |
| **Age (years)** |  |  |  |  |  |  |  |
| Mean (95% CI) | 61.2 (58.8–63.7) | 59.7 (57.3–62.0) | 0.357 |  | 57.5 (53.4–61.6) | 59.7 (56.1–63.4) | 0.409 |
| <75 | 51 (94.4) | 55 (96.5) | 0.951 |  | 19 (100.0) | 17 (94.4) | 0.978 |
| ≥75 | 3 (5.6) | 2 (3.5) |  |  | 0 (0.0) | 1 (5.6) |  |
| **Mean follow-up**  **(months, 95% CI)** | 11.8 (9.6–13.9) | 11.3 (9.2–13.3) |  |  | 12.1 (8.5–15.7) | 11.7 (7.6–15.7) |  |
| **Race, *N* (%)** |  |  | 0.262 |  |  |  | 0.067 |
| White | 29 (53.7) | 23 (40.4) |  |  | 9 (47.4) | 15 (83.3) |  |
| Asian | 21 (38.9) | 31 (54.4) |  |  | 8 (42.1) | 2 (11.1) |  |
| Other | 4 (7.4) | 3 (5.3) |  |  | 2 (10.5) | 1 (5.6) |  |
| **ECOG-PS, *N* (%)** |  |  | 1.000 |  |  |  | 0.205 |
| 0 | 25 (46.3) | 26 (45.6) |  |  | 5 (26.3) | 1 (5.6) |  |
| 1 | 29 (53.7) | 31 (54.4) |  |  | 14 (73.7) | 17 (94.4) |  |
| **Histologic type, *N* (%)** |  |  | 0.588 |  |  |  | 1.000 |
| Non-LUSC | 50 (92.6) | 50 (87.7) |  |  | 17 (89.5) | 16 (88.9) |  |
| LUSC | 4 (7.4) | 7 (12.3) |  |  | 2 (10.5) | 2 (11.1) |  |
| **Smoking status, *N* (%)** |  |  | 0.697 |  |  |  | 0.072 |
| Current | 3 (5.6) | 4 (7.0) |  |  | 1 (5.3) | 2 (11.1) |  |
| Former | 28 (51.9) | 25 (43.9) |  |  | 8 (42.1) | 13 (72.2) |  |
| Never | 23 (42.6) | 28 (49.1) |  |  | 10 (52.6) | 3 (16.7) |  |
| **Prior courses of chemotherapy, *N* (%)** |  |  | 0.103 |  |  |  | 0.141 |
| 1 | 41 (75.9) | 34 (59.6) |  |  | 13 (68.4) | 7 (38.9) |  |
| 2 | 13 (24.1) | 23 (40.4) |  |  | 6 (31.6) | 11 (61.1) |  |
| **PD-L1 expression, *N* (%)** |  |  | 0.088 |  |  |  | NR |
| TC0 and IC0 | 25 (46.3) | 36 (64.3) |  |  | NR | NR |  |
| TC1/2/3 or IC1/2/3 | 29 (53.7) | 20 (35.7) |  |  | NR | NR |  |
| **PD-L1 expression, *N* (%)** | |  | 0.531 |  |  |  | NR |
| TC0/1/2 and IC0/1/2 | 46 (85.2) | 50 (90.9) |  |  | NR | NR |  |
| TC3 or IC3 | 8 (14.8) | 5 (9.1) |  |  | NR | NR |  |
| **KRAS, *N* (%)** |  |  | 0.358 |  |  |  | 1.000 |
| Positive | 1 (2.1) | 4 (8.5) |  |  | 1 (5.9) | 1 (6.7) |  |
| Negative | 46 (97.9) | 43 (91.5) |  |  | 16 (94.1) | 14 (93.3) |  |
| **bTMB** |  |  |  |  |  |  |  |
| (mean, 95% CI) | 9.2 (5.6–12.8) | 8.1 (5.3–10.9) | 0.669 |  | 6.4 (3.4–9.4) | 16.8 (8.5–25.0) | 0.011 |
| <18 | 33 (80.5) | 34 (81.0) | 1.000 |  | 14 (93.3) | 8 (53.3) | 0.039 |
| ≥18 | 8 (19.5) | 8 (19.0) |  |  | 1 (6.7) | 7 (46.7) |  |
| **MSAF**  **(mean, 95% CI)** | 0.06 (0.04–0.07) | 0.05 (0.04–0.07) | 0.814 |  | 0.07 (0.04–0.11) | 0.09 (0.05–0.13) | 0.362 |
| **SLD, mm** |  |  |  |  |  |  |  |
| (mean, 95% CI) | 69.2 (58.6–79.7) | 76.6 (64.0–89.2) | 0.370 |  | 82.1 (63.0–101.1) | 83.6 (66.3–100.8) | 0.625 |
| <81 | 37 (68.5) | 35 (61.4) | 0.558 |  | 8 (42.1) | 10 (55.6) | 0.625 |
| ≥81 | 17 (31.5) | 22 (38.6) |  |  | 11 (57.9) | 8 (44.4) |  |
| **Metastasis sites** |  |  | 0.172 |  |  |  | 1.000 |
| 1 | 8 (14.8) | 3 (5.3) |  |  | 1 (5.3) | 0 (0.0) |  |
| >1 | 46 (85.2) | 54 (94.7) |  |  | 18 (94.7) | 18 (100) |  |
| **Best response, *N* (%)** | |  | 0.068 |  |  |  | 0.165 |
| CR | 0 (0.0) | 0 (0.0) |  |  | 1 (5.3) | 0 (0.0) |  |
| PR | 5 (9.8) | 13 (27.1) |  |  | 0 (0.0) | 3 (18.8) |  |
| SD | 23 (45.1) | 20 (41.7) |  |  | 6 (31.6) | 6 (37.5) |  |
| PD | 23 (45.1) | 15 (31.2) |  |  | 12 (63.2) | 7 (43.8) |  |

^a^Patients in OAK cohort.

^b^Patients in POPLAR cohort.

ECOG-PS, Eastern Cooperative Oncology Group Performance Status; LUSC, lung squamous cell carcinoma; SLD, sum of the longest diameter; PD-L1, programmed cell death ligand 1; CR, complete response; PR, partial response; SD, stable disease; PD, progressive disease; CI, confidence interval; NR, not reported; bTMB indicates blood-based tumor mutation burden; MSAF indicates ctDNA maximum somatic allele frequency. TC0 and IC0 indicates that less than 1% of tumor cells and less than 1% of tumor infiltrating immune cells expressed PD-L1; TC0/1/2 and IC0/1/2 indicates that less than 50% of tumor cells and less than 10% of tumor infiltrating immune cells expressed PD-L1; TC1/2/3 or IC1/2/3 indicates that over 1% of tumor cells or over 1% of tumor infiltrating immune cells expressed PD-L1; TC3 or IC3 indicates that over 50% of tumor cells or over 10% of tumor infiltrating immune cells expressed PD-L1.

**Table S3.** Clinical characteristics of the EGFR wild-type and EGFR mutant subgroups across treatment arms.

| **Characteristic** | ***N* (%) ^a^** | | | |  | |  | ***N* (%) ^b^** | |  |
| --- | --- | --- | --- | --- | --- | --- | --- | --- | --- | --- |
|  | **EGFR wild-type** | | **EGFR mutant** | | ***P* value** | |  | **EGFR wild-type** | **EGFR mutant** | ***P* value** |
| **No. (%)** | | 713 (86.5) | | 111 (13.5) | |  |  | 217 (85.4) | 37 (14.6) |  |
| **Sex, *N* (%)** | |  | |  | | <0.001 |  |  |  | 0.551 |
| Female | | 261 (36.6) | | 64 (57.7) | |  |  | 85 (39.2) | 17 (45.9) |  |
| Male | | 452 (63.4) | | 47 (42.3) | |  |  | 132 (60.8) | 20 (54.1) |  |
| **Age (years)** | |  | |  | |  |  |  |  |  |
| Mean (95%CI) | | 63.7 (63.0–64.3) | | 60.4 (58.6–62.1) | | 0.002 |  | 62.3 (61.1–63.4) | 58.6 (56.0–61.2) | 0.021 |
| <75 | | 629 (88.2) | | 106 (95.5) | | 0.033 |  | 197 (90.8) | 36 (97.3) | 0.314 |
| ≥75 | | 84 (11.8) | | 5 (4.5) | |  |  | 20 (9.2) | 1 (2.7) |  |
| **Mean follow-up**  **(months, 95% CI)** | | 11.4 (10.8–11.9) | | 11.5 (10.1–13.0) | | 0.839 |  | 11.3 (10.3–12.4) | 11.9 (9.4–14.4) | 0.664 |
| **Race, *N* (%)** | |  | |  | | <0.001 |  |  |  | 0.022 |
| White | | 524 (73.5) | | 52 (46.8) | |  |  | 176 (81.1) | 24 (64.9) |  |
| Asian | | 125 (17.5) | | 52 (46.8) | |  |  | 23 (10.6) | 10 (27.0) |  |
| Other | | 64 (9.0) | | 7 (6.3) | |  |  | 18 (8.3) | 3 (8.1) |  |
| **ECOG-PS, *N* (%)** | |  | |  | | 0.050 |  |  |  | 0.151 |
| 0 | | 255 (35.8) | | 51 (45.9) | |  |  | 142 (65.4) | 31 (83.8) |  |
| 1 | | 458 (64.2) | | 60 (54.1) | |  |  | 70 (32.3) | 17 (45.9) |  |
| **Histologic type, *N* (%)** | | | |  | | <0.001 |  |  |  | 0.006 |
| Non-LUSC | | 502 (72.9) | | 100 (90.1) | |  |  | 141 (65.0) | 33 (89.2) |  |
| LUSC | | 193 (27.1) | | 11 (9.9) | |  |  | 76 (35.0) | 4 (10.8) |  |
| **Smoking status, *N* (%)** | | | |  | | <0.001 |  |  |  | 0.027 |
| Current | | 115 (16.1) | | 7 (6.3) | |  |  | 41 (18.9) | 3 (8.1) |  |
| Former | | 497 (69.7) | | 53 (47.7) | |  |  | 138 (63.6) | 21 (56.8) |  |
| Never | | 101 (14.2) | | 51 (45.9) | |  |  | 38 (17.5) | 13 (35.1) |  |
| **Prior courses of chemotherapy, *N* (%)** | | | | | | 0.053 |  |  |  | 0.151 |
| 1 | | 546 (76.6) | | 75 (67.6) | |  |  | 147 (67.7) | 20 (54.1) |  |
| 2 | | 167 (23.4) | | 36 (32.4) | |  |  | 70 (32.3) | 17 (45.9) |  |
| **PD-L1 expression, *N* (%)** | |  | |  | | 0.026 |  |  |  | NR |
| TC0 and IC0 | | 308 (43.6) | | 61 (55.5) | |  |  | NR | NR |  |
| TC1/2/3 or IC1/2/3 | | 399 (56.4) | | 49 (44.5) | |  |  | NR | NR |  |
| **PD-L1 expression, *N* (%)** | |  | |  | | 0.262 |  |  |  | NR |
| TC0/1/2 and IC0/1/2 | | 589 (83.3) | | 96 (88.1) | |  |  | NR | NR |  |
| TC3 or IC3 | | 118 (16.7) | | 13 (11.9) | |  |  | NR | NR |  |
| **KRAS, *N* (%)** | |  | |  | | 0.008 |  |  |  | 0.147 |
| Positive | | 100 (16.3) | | 5 (5.3) | |  |  | 36 (18.4) | 2 (6.2) |  |
| Negative | | 512 (83.7) | | 89 (94.7) | |  |  | 160 (81.6) | 30 (93.8) |  |
| **bTMB** | |  | |  | |  |  |  |  |  |
| (mean, 95% CI) | | 9.2 (8.5–9.9) | | 8.6 (6.3–10.8) | | 0.565 |  | 10.0 (8.5–11.4) | 11.4 (7.6–16.1) | 0.508 |
| <18 | | 414 (74.3) | | 67 (80.7) | | 0.262 |  | 126 (70.4) | 22 (73.3) |  |
| ≥18 | | 143 (25.7) | | 16 (19.3) | |  |  | 53 (29.6) | 8 (26.7) |  |
| **MSAF**  **(mean, 95% CI)** | | 0.06 (0.04–0.07) | | 0.06 (0.05–0.06) | | 0.722 |  | 0.06 (0.5–0.07) | 0.08 (0.6–0.10) | 0.061 |
| **SLD, mm** | |  | |  | |  |  |  |  |  |
| (mean, 95% CI) | | 77.1 (73.6–80.8) | | 73.0 (65.4–81.1) | | 0.358 |  | 81.5(75.6–87.6) | 82.8(71.1–94.7) | 0.845 |
| <81 | | 441 (61.8) | | 72 (64.9) | | 0.607 |  | 126 (58.1) | 18 (48.6) | 0.875 |
| ≥81 | | 272 (38.2) | | 39 (35.1) | |  |  | 91 (41.9) | 19 (51.4) |  |
| **Metastasis sites, *N* (%)** | | | |  | | 0.357 |  |  |  | 0.160 |
| 1 | | 97 (13.6) | | 11 (9.9) | |  |  | 26 (12.0) | 1 (2.7) |  |
| >1 | | 616 (86.4) | | 100 (90.1) | |  |  | 191 (88.0) | 36 (97.3) |  |
| **Best response, *N* (%)** | | | |  | | 0.554 |  |  |  | 0.045 |
| CR | | 6 (1.0) | | 0 (0.0) | |  |  | 0 (0.0) | 1 (2.9) |  |
| PR | | 88 (14.1) | | 18 (18.2) | |  |  | 33 (17.5) | 3 (8.6) |  |
| SD | | 273 (43.8) | | 43 (43.4) | |  |  | 76 (40.2) | 12 (34.3) |  |
| PD | | 257 (41.2) | | 38 (38.4) | |  |  | 80 (42.3) | 19 (54.3) |  |

^a^Patients in OAK cohort.

^b^Patients in POPLAR cohort.

Valid data for stratification of EGFR status were available of 1,078 patients without sensitizing ALK mutations; 86.3% of these patients’ tumors were EGFR wild-type (50.1% in the atezolizumab group and 49.9% in the docetaxel group), and EGFR mutations were observed in 13.7% of tumors (49.3% in the atezolizumab group and 50.7% in the docetaxel group). ECOG-PS, Eastern Cooperative Oncology Group Performance Status; LUSC, lung squamous cell carcinoma; SLD, sum of the longest diameter; PD-L1, programmed cell death ligand 1; CR, complete response; PR, partial response; SD, stable disease; PD, progressive disease; CI, confidence interval; NR, not reported; bTMB indicates blood-based tumor mutation burden; MSAF indicates ctDNA maximum somatic allele frequency. TC0 and IC0 indicates that less than 1% of tumor cells and less than 1% of tumor infiltrating immune cells expressed PD-L1; TC0/1/2 and IC0/1/2 indicates that less than 50% of tumor cells and less than 10% of tumor infiltrating immune cells expressed PD-L1; TC1/2/3 or IC1/2/3 indicates that over 1% of tumor cells or over 1% of tumor infiltrating immune cells expressed PD-L1; TC3 or IC3 indicates that over 50% of tumor cells or over 10% of tumor infiltrating immune cells expressed PD-L

**Table S4.** Clinical characteristics of EGFR wild-type patients below the bTMB-MSAF algorithm < 20 cut-point.

| **Characteristic** | | ***N* (%) ^a^** | | | | |  |  | ***N* (%) ^b^** | |  |
| --- | --- | --- | --- | --- | --- | --- | --- | --- | --- | --- | --- |
|  |  | **Atezolizumab** | | **Docetaxel** | | ***P* value** | |  | **Atezolizumab** | **Docetaxel** | ***P* value** |
| **No. (%)** | 199 (51.4) | | 188 (48.6) | |  | | |  | 61 (50.4) | 60 (49.6) |  |
| **Sex, *N* (%)** |  | |  | | 0.824 | | |  |  |  | 0.113 |
| Female | 73 (36.7) | | 72 (38.3) | |  | | |  | 30 (49.2) | 20 (33.3) |  |
| Male | 126 (63.3) | | 116 (61.7) | |  | | |  | 31 (50.8) | 40 (66.7) |  |
| **Age (years)** |  | |  | |  | | |  |  |  |  |
| Mean (95% CI) | 64.4 (63.1–65.7) | | 64.5 (63.1–65.8) | | 0.946 | | |  | 64.0 (61.8–66.2) | 61.7 (59.1–64.1) | 0.167 |
| <75 | 168 (84.4) | | 170 (90.4) | | 0.105 | | |  | 55 (90.2) | 53 (88.3) | 0.975 |
| ≥75 | 31 (15.6) | | 18 (9.6) | |  | | |  | 6 (9.8) | 7 (11.7) |  |
| **Mean follow-up**  **(months, 95% CI)** | 13.8 (12.6–14.8) | | 10.0 (9.0–11.1) | |  | | |  | 10.1 (8.3–11.8) | 13.7 (11.8–15.6) |  |
| **Race, *N* (%)** |  | |  | | 0.529 | | |  |  |  | 0.454 |
| White | 146 (73.4) | | 139 (73.9) | |  | | |  | 51 (83.6) | 47 (78.3) |  |
| Asian | 39 (19.6) | | 31 (16.5) | |  | | |  | 4 (6.6) | 8 (13.3) |  |
| Other | 14 (7.0) | | 18 (9.6) | |  | | |  | 6 (9.8) | 5 (8.3) |  |
| **ECOG-PS, *N* (%)** |  | |  | | 0.528 | | |  |  |  | 0.946 |
| 0 | 68 (34.2) | | 71 (37.8) | |  | | |  | 21 (34.4) | 22 (36.7) |  |
| 1 | 131 (65.8) | | 117 (62.2) | |  | | |  | 40 (65.6) | 38 (63.3) |  |
| **Histologic type, *N* (%)** | | |  | | 1.000 | | |  |  |  | 1.000 |
| Non-LUSC | 141 (70.9) | | 134 (71.3) | |  | | |  | 37 (60.7) | 37 (61.7) |  |
| LUSC | 58 (29.1) | | 54 (28.7) | |  | | |  | 24 (39.3) | 23 (38.3) |  |
| **Smoking status, *N* (%)** | | |  | | 0.464 | | |  |  |  | 0.305 |
| Current | 29 (14.6) | | 31 (16.5) | |  | | |  | 12 (19.7) | 15 (25.0) |  |
| Former | 142 (71.4) | | 138 (73.4) | |  | | |  | 37 (60.7) | 39 (65.0) |  |
| Never | 28 (14.1) | | 19 (10.1) | |  | | |  | 12 (19.7) | 6 (10.0) |  |
| **Prior courses of chemotherapy, *N* (%)** | | | | | 1.000 | | |  |  |  | 0.064 |
| 1 | 151 (75.9) | | 142 (75.5) | |  | | |  | 48 (78.7) | 37 (61.7) |  |
| 2 | 48 (24.1) | | 46 (24.5) | |  | | |  | 13 (21.3) | 23 (38.3) |  |
| **PD-L1 expression, *N* (%)** |  | |  | | 0.905 | | |  |  |  | NR |
| TC0 and IC0 | 84 (42.4) | | 81 (43.5) | |  | | |  | NR | NR |  |
| TC1/2/3 or IC1/2/3 | 114 (57.6) | | 105 (56.5) | |  | | |  | NR | NR |  |
| **PD-L1 expression, *N* (%)** |  | |  | | 0.709 | | |  |  |  | NR |
| TC0/1/2 and IC0/1/2 | 161 (80.9) | | 155 (82.9) | |  | | |  | NR | NR |  |
| TC3 or IC3 | 38 (19.1) | | 32 (17.1) | |  | | |  | NR | NR |  |
| **KRAS, *N* (%)** |  | |  | | 0.509 | | |  |  |  | 0.299 |
| Positive | 28 (14.1) | | 32 (17.0) | |  | | |  | 7 (11.5) | 12 (20.0) |  |
| Negative | 171 (85.9) | | 156 (83.0) | |  | | |  | 54 (88.5) | 48 (80.0) |  |
| **bTMB** |  | |  | |  | | |  |  |  |  |
| (mean, 95% CI) | 10.4 (9.2–11.7) | | 10.2 (9.1–11.4) | | 0.798 | | |  | 12.7 (9.7–15.8) | 12.3 (9.8–15.1) | 0.858 |
| <18 | 162 (81.4) | | 151 (80.3) | | 0.887 | | |  | 42 (68.9) | 47 (78.3) | 0.329 |
| ≥18 | 37 (18.6) | | 37 (19.7) | |  | | |  | 19 (31.1) | 13 (21.7) |  |
| **MSAF**  **(mean, 95% CI)** | 0.043  (0.039–0.046) | | 0.043  (0.039–0.046) | | 0.075 | | |  | 0.038  (0.031–0.046) | 0.042  (0.034–0.051) | 0.528 |
| **SLD, mm** |  | |  | |  | | |  |  |  |  |
| (mean, 95% CI) | 73.2 (67.3–80.0) | | 73.2 (67.3–80.0) | | 0.672 | | |  | 84.6 (72.8–98.1) | 82.5 (69.8–96.3) | 0.823 |
| <81 | 133 (67.2) | | 133 (67.2) | | 0.489 | | |  | 34 (55.7) | 35 (58.3) | 0.917 |
| ≥81 | 65 (32.8) | | 65 (32.8) | |  | | |  | 27 (44.3) | 25 (41.7) |  |
| **Metastasis sites, *N* (%)** | | |  | | 0.349 | | |  |  |  | 0.536 |
| 1 | 32 (16.1) | | 32 (16.1) | |  | | |  | 5 (8.2) | 8 (13.3) |  |
| >1 | 167 (83.9) | | 167 (83.9) | |  | | |  | 56 (91.8) | 52 (86.7) |  |
| **Best response, *N* (%)** | | |  | | 0.013 | | |  |  |  | 0.600 |
| CR | 4 (2.1) | | 4 (2.1) | |  | | |  | 0 (0.0) | 0 (0.0) |  |
| PR | 30 (16.0) | | 30 (16.0) | |  | | |  | 8 (14.3) | 12 (21.4) |  |
| SD | 74 (39.6) | | 74 (39.6) | |  | | |  | 24 (42.9) | 23 (41.1) |  |
| PD | 79 (42.2) | | 79 (42.2) | |  | | |  | 24 (42.9) | 21 (37.5) |  |

^a^Patients in OAK cohort.

^b^Patients in POPLAR cohort.

ECOG-PS, Eastern Cooperative Oncology Group Performance Status; LUSC, lung squamous cell carcinoma; SLD, sum of the longest diameter; PD-L1, programmed cell death ligand 1; CR, complete response; PR, partial response; SD, stable disease; PD, progressive disease; CI, confidence interval; NR, not reported; bTMB indicates blood-based tumor mutation burden; MSAF indicates ctDNA maximum somatic allele frequency. TC0 and IC0 indicates that less than 1% of tumor cells and less than 1% of tumor infiltrating immune cells expressed PD-L1; TC0/1/2 and IC0/1/2 indicates that less than 50% of tumor cells and less than 10% of tumor infiltrating immune cells expressed PD-L1; TC1/2/3 or IC1/2/3 indicates that over 1% of tumor cells or over 1% of tumor infiltrating immune cells expressed PD-L1; TC3 or IC3 indicates that over 50% of tumor cells or over 10% of tumor infiltrating immune cells expressed PD-L1.

**Supplementary Table S5.** Summary of overall survival analyses of overlapping subsets between bTMB-MSAF algorithm and PD-L1 expression stratified by EGFR mutation status.

| **Subset** | **Score<20** | | **Score≥20** | |
| --- | --- | --- | --- | --- |
|  | **HR (95% CI)** | ***P* value** | **HR (95% CI)** | ***P* value** |
| **EGFR wild–type** |  |  |  |  |
| TC0 and IC0 | 0.50 (0.35–0.73) | <0.001 | 1.06 (0.64–1.75) | 0.817 |
| TC1/2/3 or IC1/2/3 | 0.64 (0.46–0.89) | 0.007 | 0.82 (0.52–1.29) | 0.382 |
| TC0/1/2 and IC0/1/2 | 0.61 (0.47–0.79) | <0.001 | 1.11 (0.77–1.61) | 0.571 |
| TC3 or IC3 | 0.44 (0.23–0.81) | 0.007 | 0.39 (0.17–0.94) | 0.036 |
| **EGFR mutant** |  |  |  |  |
| TC0 and IC0 | 1.09 (0.49–2.41) | 0.830 | 0.94 (0.11–8.17) | 0.954 |
| TC1/2/3 or IC1/2/3 | 0.51 (0.16–1.58) | 0.242 | 2.04 (0.23–17.68) | 0.519 |
| TC0/1/2 and IC0/1/2 | 1.01 (0.51–1.98) | 0.978 | 2.68 (0.88–8.11) | 0.082 |
| TC3 or IC3 | 0.17 (0.01–1.92) | 0.151 | NA | NA |

HR, hazard ratio; CI, confidence interval; NA, not applicable. TC0 and IC0 indicates that less than 1% of tumor cells and less than 1% of tumor infiltrating immune cells expressed PD-L1; TC0/1/2 and IC0/1/2 indicates that less than 50% of tumor cells and less than 10% of tumor infiltrating immune cells expressed PD-L1; TC1/2/3 or IC1/2/3 indicates that over 1% of tumor cells and over 1% of tumor infiltrating immune cells expressed PD-L1; TC3 or IC3 indicates that over 50% of tumor cells or over 10% of tumor infiltrating immune cells expressed PD-L1.

**Supplementary Table S6.** Association of clinicopathological factors with survival of atezolizumab-treated patients.

| **Variable** | ***N*** | **Univariate analysis** | |
| --- | --- | --- | --- |
|  |  | **HR (95% CI)** | ***P* value** |
| Sex (female *vs.* male) | 214 *vs.*353 | 0.76 (0.62–0.95) | 0.015 |
| Race (Asian *vs.* other) | 108 *vs.*459 | 0.65 (0.48–0.87) | 0.004 |
| ECOG–PS (0 *vs.* 1) | 201 *vs.*366 | 0.59 (0.47–0.74) | 0.000 |
| Smoking status (never *vs.* current or previous) | 109 *vs.*458 | 0.76 (0.57–1.00) | 0.0517 |
| Histology (non–LUSC *vs.* LUSC) | 406 *vs.*161 | 0.71 (0.57–0.89) | 0.003 |
| Age (≥75 *vs.* <75) | 65 *vs.*502 | 0.99 (0.72–1.37) | 0.948 |
| PD–L1 (TC1/2/3 or IC1/2/3 *vs.* TC0 and IC0) | 239 *vs.*180 | 0.89 (0.70–1.14) | 0.352 |
| PD–L1 (TC3 or IC3 *vs.* TC0/1/2 and IC0/1/2) | 72 *vs.*346 | 0.61 (0.43–0.86) | 0.005 |
| MSAF (<0.04 *vs.* 0.04–0.08 *vs.* >0.08) | 251 *vs.*109 *vs.*162 | 0.78 (0.72–0.85) | 0.000 |
| bTMB (<7 *vs.* ≥7) | 155 *vs.*272 | 0.63 (0.49–0.81) | 0.000 |
| Response (CR *vs.* PR *vs.* SD *vs.* PD) | 7 *vs.*73 *vs.*201 *vs.*246 | 0.38 (0.32–0.45) | 0.000 |
| Metastasis sites (1 *vs.* 2–3 *vs.* >3) | 87 *vs.*316 *vs.*164 | 0.63 (0.53–0.74) | 0.000 |
| SLD (<100 mm *vs.* 100–150 mm *vs.* >150 mm) | 420 *vs.*101 *vs.*45 | 0.60 (0.52–0.70) | 0.000 |
| Prior chemotherapy (1 *vs.* 2) | 412 *vs.*155 | 0.92 (0.73–1.15) | 0.470 |

ECOG-PS, Eastern Cooperative Oncology Group Performance Status; LUSC, lung squamous cell carcinoma; SLD, sum of the longest diameter; PD-L1, programmed cell death ligand 1; CR, complete response; PR, partial response; SD, stable disease; PD, progressive disease; HR, hazard ratio; CI, confidence interval. bTMB indicates blood-based mutation burden. MSAF indicates ctDNA maximum somatic allele frequency. TC0 and IC0 indicates that less than 1% of tumor cells and less than 1% of tumor infiltrating immune cells expressed PD-L1; TC0/1/2 and IC0/1/2 indicates that less than 50% of tumor cells and less than 10% of tumor infiltrating immune cells expressed PD-L1; TC1/2/3 or IC1/2/3 indicates that over 1% of tumor cells or over 1% of tumor infiltrating immune cells expressed PD-L1; TC3 or IC3 indicates that over 50% of tumor cells or over 10% of tumor infiltrating immune cells expressed PD-L1.

**Supplementary Table S7.** Summary of overall survival analysis of atezolizumab-treated patients with oncogenic driver mutation versus patients with corresponding wild-type tumor.

| **Oncogene** | **Univariate analysis** | |
| --- | --- | --- |
|  | **HR (95% CI)** | ***P* value** |
| TP53 | 1.44 (1.14–1.82) | 0.002 |
| LRP1B | 1.10 (0.85–1.42) | 0.476 |
| DNMT3A | 0.79 (0.59–1.05) | 0.106 |
| FAT3 | 0.93 (0.67–1.30) | 0.681 |
| SPTA1 | 1.02 (0.74–1.40) | 0.907 |
| NF1 | 1.10 (0.76–1.58) | 0.611 |
| STAG2 | 0.77 (0.53–1.12) | 0.170 |
| KEAP1 | 1.86 (1.37–2.53) | <0.001 |
| MLL2 | 1.38 (0.99–1.93) | 0.056 |
| FAT1 | 1.18 (0.85–1.65) | 0.321 |
| TSC1 | 0.73 (0.48–1.12) | 0.146 |
| MLL3 | 1.35 (0.95–1.91) | 0.096 |
| PTPRD | 0.83 (0.55–1.25) | 0.380 |
| ATM | 0.65 (0.43–0.98) | 0.038 |
| EPHA6 | 1.11 (0.74–1.68) | 0.618 |
| SMARCA4 | 1.28 (0.84–1.97) | 0.252 |
| TET2 | 0.91 (0.57–1.45) | 0.682 |
| PREX2 | 1.31 (0.87–1.98) | 0.198 |
| KRAS | 1.19 (0.80–1.77) | 0.400 |

HR, hazard ratio; CI, confidence interval.

**Supplementary Table S8.** Summary of concordance-index of the predictors.

| **Variable** | **OAK** | **POPLAR** | **Two cohorts combined** |
| --- | --- | --- | --- |
| Sex | 0.538 | 0.512 | 0.531 |
| Race | 0.531 | 0.546 | 0.535 |
| ECOG-PS | 0.572 | 0.559 | 0.558 |
| SLD | 0.559 | 0.586 | 0.566 |
| Histology | 0.538 | 0.535 | 0.538 |
| PD-L1 | 0.511 | NA | NA |
| Metastasis sites | 0.565 | 0.594 | 0.573 |
| TP53 | 0.555 | 0.545 | 0.553 |
| KEAP1 | 0.534 | 0.536 | 0.535 |
| ATM | 0.524 | 0.548 | 0.529 |
| Response | 0.681 | 0.715 | 0.691 |
| Nomogram A | 0.650 | 0.669 | 0.646 |
| Nomogram B | 0.651 | NA | NA |
| Nomogram C | 0.749 | NA | NA |

ECOG-PS, Eastern Cooperative Oncology Group Performance Status; SLD, sum of the longest diameter; PD-L1, programmed cell death ligand 1; NA, not applicable.

**Supplementary Table S9.** Summary of receiver operator characteristics analyses in the nomograms.

| **Nomogram** | **OAK**  **(AUC)** | **POPLAR (AUC)** | **Two cohorts combined (AUC)** |
| --- | --- | --- | --- |
| **Nomogram A** |  |  |  |
| 1-year OS | 0.694 | 0.693 | 0.684 |
| 2-year OS | 0.721 | 0.726 | 0.696 |
| 3-year OS | 0.733 | 0.711 | 0.714 |
| **Nomogram B** |  |  |  |
| 1-year OS | 0.701 | NA | NA |
| 2-year OS | 0.738 | NA | NA |
| 3-year OS | 0.756 | NA | NA |
| **Nomogram C** |  |  |  |
| 1-year OS | 0.855 | NA | NA |
| 2-year OS | 0.813 | NA | NA |
| 3-year OS | 0.860 | NA | NA |

OS, overall survival; NA, not applicable. AUC indicates area under the receiver operating characteristic curve.

| **Nomogram** | **OAK** | **Validation cohort 1** | **Validation cohort 2** |
| --- | --- | --- | --- |
| Nomogram B | 0.651 | 0.670 | 0.630 |
| Nomogram C | 0.749 | 0.724 | 0.771 |

**Supplementary Table S10**. Concordance-index for the internal validation of nomograms B and C.

**Table S11.** Summary of receiver operator characteristics analyses in the internal validation of nomograms B and C.

| **Nomogram** | **OAK**  **(AUC)** | **Validation cohort 1 (AUC)** | | **Validation cohort 2**  **(AUC)** |
| --- | --- | --- | --- | --- |
| **Nomogram B** |  |  |  | |
| 1-year OS | 0.701 | 0.736 | 0.668 | |
| 2-year OS | 0.738 | 0.728 | 0.746 | |
| 3-year OS | 0.756 | 0.778 | 0.746 | |
| **Nomogram C** |  |  |  | |
| 1-year OS | 0.855 | 0.835 | 0.882 | |
| 2-year OS | 0.813 | 0.757 | 0.874 | |
| 3-year OS | 0.860 | 0.869 | 0.874 | |

OS, overall survival; AUC indicates area under the receiver operating characteristic curve.
